# A systematic review exploring the diversity and food security potential of wild edible plants in Ethiopia

**DOI:** 10.1038/s41598-024-67421-y

**Published:** 2024-08-01

**Authors:** Daniel Tadesse, Getinet Masresha, Ermias Lulekal, Melaku Wondafrash

**Affiliations:** 1 Department of Plant Sciences, University of Gondar, Gondar, Ethiopia; 2 Department of Biology, University of Gondar, Gondar, Ethiopia; 3 Department of Plant Biology and Biodiversity Management, Addis Ababa University, Addis Ababa, Ethiopia

**Keywords:** Plant diversity, Food security, Indigenous knowledge, Wild edible plants, Ethiopia, Ecology, Plant sciences, Ecology, Environmental sciences

## Abstract

Wild edible plants (WEPs) are important food sources globally due to their accessibility and affordability. In Ethiopia, where diverse cultural groups consume WEPs, this systematic review explores their diversity, edible parts, and role in supporting food security. The review examined 38 original studies on the ethnobotany of WEPs in Ethiopia from 2000 to 2022. It identified a total of 651 WEP species from 343 genera and 94 families, with the Fabaceae family having the most species (51). Herbs and shrubs were the predominant growth habits, and fruits were the most consumed plant parts. The review prioritized nine WEP species for cultivation and promotion. However, threats such as overgrazing, agricultural expansion, and the use of woody species for construction, firewood, and charcoal have depleted WEP resources and eroded traditional knowledge about their use. The review suggests that WEPs have the potential to contribute to food and nutritional security in Ethiopia if these threats are effectively managed. However, the limited coverage of ethnobotanical studies on WEPs requires further investigation. The study recommends integrating the prioritized WEPs into the national food system for promotion, cultivation, and nutrient analysis to evaluate their nutritional bioavailability.

## Introduction

Ethiopia boasts a diverse range of biodiversity, with two of the world’s 34 biodiversity hotspots—the Eastern Afromontane and the Horn of Africa hotspots—located within its borders^1,2^. The country is home to a wide array of wild edible plants (WEPs). The Food and Agriculture Organization (FAO) defines WEPs as “plants that grow spontaneously in self-maintaining populations in natural or seminatural ecosystems and can exist independently of direct human action”^3^. These edible plants can be found thriving on farmland, fallow land, and uncultivated areas^4,5^.

WEPs offer numerous advantages, including a diverse variety, convenient accessibility, consistent availability, proven reliability, and minimal management requirements^6,7^. Ensuring food security at all levels—individual, household, national, regional, and global—is a paramount concern^8^, and diversifying food sources is crucial for achieving this goal, particularly in Africa^9^. In developing countries like Ethiopia, many individuals rely heavily on WEPs as their primary food source due to inadequate access to sufficient food resources^10,11^. This underscores the vital role that WEPs play in local food systems, contributing significantly to the food and nutrition security of impoverished populations^12–14^.

Furthermore, the harvesting and trading of WEPs have the potential to create employment opportunities and generate income in rural areas^14–16^. WEPs also offer promising alternatives for nutrient supplementation in various cultivated fruits^17^, as they are rich sources of carbohydrates, minerals^18^, proteins, fats^19^, phenols, carotenoids, and essential vitamins like E and C^20^. This makes them instrumental in addressing the issue of micronutrient deficiency, commonly referred to as "hidden hunger", which impacts a significant portion of the global population^21,22^.

There is a growing awareness of the medicinal and nutritional benefits associated with WEPs^23,24^. Many WEPs contain valuable plant secondary metabolites, which can help prevent deficiency diseases, and protect against chronic illnesses like cancer^25,26^. The presence of these secondary metabolites positions WEPs as promising candidates for the development of nutraceuticals with potentially health-promoting properties^26^.

Numerous studies have indicated that WEPs often possess superior nutritional profiles compared to traditional cultivated crops^27–31^. In Ethiopia, recent assessments have revealed that WEPs can provide significant amounts of essential nutrients to fulfill recommended daily intakes^32^. However, the utilization of WEPs is hindered by the presence of anti-nutritional and toxic compounds such as nitrites, phytates, oxalates, and saponins^33^. Additionally, some WEPs have been found to accumulate toxic metals beyond safe limits, rendering them unsuitable for human consumption^34,35^.

Various reports have presented conflicting figures regarding the number of WEPs consumed globally. Kunkel^36^ claimed there were 12,500 edible species, while Rapoport and Drausal^37^ reviewed 27,000 edible species, and Wilson^38^ reported 75,000 edible species. A more conservative estimate was provided by the Royal Botanic Gardens, Kew^39^, which identified 5538 plant species that are consumed by humans, without specifying whether they are domesticated or wild. This lack of consensus highlights the uncertainty surrounding the total number of WEPs. In Ethiopia, it is estimated that 30–40% of the population regularly consumes WEPs, with this number reaching up to 56–67% in certain regions^40^. This indicates the significant role that WEPs play in the diets of many people in Ethiopia.

Globally, WEPs face threats to their natural habitats due to various human activities^41–43^. In Africa, these threats present challenges for the approximately 80% of rural populations who rely on wild sources for food^44^. The lack of comprehensive conservation assessments hinders effective action to address these threats. It is crucial to identify common threats and management practices specific to Ethiopia to ensure the sustainable use of WEPs.

Despite their widespread consumption across different cultural groups in Ethiopia, there is a lack of comprehensive ethnobotanical studies focusing on WEPs. The only thorough review conducted was nearly 12 years ago, which documented the consumption of 413 WEPs^45^. According to this review, the scope of ethnobotanical studies on Ethiopian WEPs is limited, covering only about 5 percent of the 494 districts in the country. This percentage is notably small considering Ethiopia’s vast geographic expanse, ethnic diversity, and cultural richness. Further research and documentation are necessary to fully understand the significance and utilization of WEPs in Ethiopia.

The existing limited research fails to fully capture the diversity, utilization, and contribution of WEPs to food and nutritional security. It is imperative to conduct a thorough review of ethnobotanical studies on WEPs in Ethiopia to address the following key questions: (I) What is the current taxonomic diversity of WEPs in Ethiopia? (II) What is the geographical distribution of WEPs in Ethiopia based on studies conducted so far? (III) What are the major threats to WEPs? (IV) Which WEPs should be prioritized for cultivation and promotion to enhance food security? By answering these questions, we can gain a deeper understanding of the importance of WEPs in Ethiopia and prioritize their cultivation and promotion at the national level.

### Geological history, landscape, and climatic features of Ethiopia

The geological history of Ethiopia is marked by periods of highland uplift and rift formation. The Great Rift Valley, which started to uplift due to volcanic forces 75 million years ago, divides the highlands into northwestern and southeastern regions^46^. This geological barrier has restricted the immigration of many taxa, including plants, birds, amphibians, reptiles, and insects, while also creating novel habitats like the Rift Valley lakes that provide homes for diverse taxa^47,48^.

Ethiopia is a topographically complex region, with elevations ranging from 125 m below sea level in the east to about 4533 m above sea level in the north^49^. This large elevational range has led to varied topography and climate, resulting in a heterogeneous landscape with high habitat diversity, species diversity, and centers of endemism. While plant diversity is lower in the Ethiopian highlands compared to the lowlands, the highlands are nonetheless centers of endemism due to their geographical isolation and unique climatic conditions^50–52^.

Ethiopia’s climate is characterized by a rainy season from June to September, and a dry season from October to April. Rainfall generally increases from north to south and east to west, with an average annual rainfall of 600 mm in the northeast and 2000 mm in the southwest^53^. This significant climate variability is responsible for the wide range of vegetation types across the country. Ethiopia's proximity to the equator and the complexity of its topography also play a role in regulating its temperature^54^.

### Vegetation and flora of Ethiopia

Ethiopia is home to a diverse array of vegetation types, classified into 12 major categories based on elevation and rainfall patterns^55^. This range of vegetation spans from high-altitude Afroalpine ecosystems in the central highlands to the arid lowlands in the east and the rain forests in the west. These diverse habitats harbor rare and endangered species and exhibit high levels of endemism in their floral compositions^55^. However, studies have confirmed that all of Ethiopia’s natural vegetation types are under severe threat^56,57^. The rapid depletion of forest resources has led to a significant decline in biodiversity, with some species facing the risk of local extinction^58^.

Ethiopia boasts the fifth-largest floral composition in tropical Africa^59^. The country’s flora comprises 6027 vascular plant species^60^, of which 476 species are endemic, belonging to 69 families and 224 genera^61–68^. The high number of endemic and near-endemic plant species in Ethiopia can be attributed to the environmental heterogeneity created by the country’s complex topography, which provides diverse local habitats that serve as micro-refugia for species during periods of extreme environmental change^51,69^.

### Cultures, peoples, and socio-economic activity of Ethiopia

Ethiopia’s unique geographical position, complex topography, environmental heterogeneity, and ecological conditions have provided a suitable environment for a wide range of life forms to thrive^70,71^. As a result, Ethiopia is one of the world’s most ethnically and culturally diverse countries, with over 70 different languages spoken and more than 80 distinct ethnic groups^72,73^. This remarkable cultural diversity has contributed to the high diversity of traditional knowledge and practices among the people, including the use of WEPs^74^.

Ethiopia is an ancient agrarian country located between 3° and 15° N and 33° and 48° E, covering an area of 1.13 million square kilometers. In 2007, the population of Ethiopia was 74.9 million, with an annual growth rate of 2.6%^75^. This makes Ethiopia the second-most populous nation in Sub-Saharan Africa. According to the same report, 84% of the population lives in rural areas and is engaged in subsistence agriculture. The economy of Ethiopia is predominantly based on traditional subsistence agriculture, which is vulnerable to frequent droughts. Consequently, food security and natural resource degradation are among the major challenges that Ethiopia has faced, leading many people to really on WEPs grown in their local environments.

## Methodology

### Study plan and data sources

To conduct a comprehensive review of the literature on WEPs in Ethiopia, a systematic search was performed across various reputable databases, including Scopus, MEDLINE, Cochrane, EMBASE, Web of Science, and Google Scholar. Additionally, further studies on WEPs in Ethiopia were identified by searching the library databases of Addis Ababa University and University of Bonn. The search terms used to identify relevant studies on WEPs in Ethiopia included phrases such as ‘wild food’, ‘wild edible plants of Ethiopia’, ‘uncultivated food’, ‘underutilized fruits’, and ‘vegetables’, among others. This review encompasses all studies on WEPs published from 2000 to 2022. It is worth noting that no ethnobotanical investigations of WEPs in Ethiopia were accessible through search engines before the year 2000.

### Data extraction

**Figure 1:**
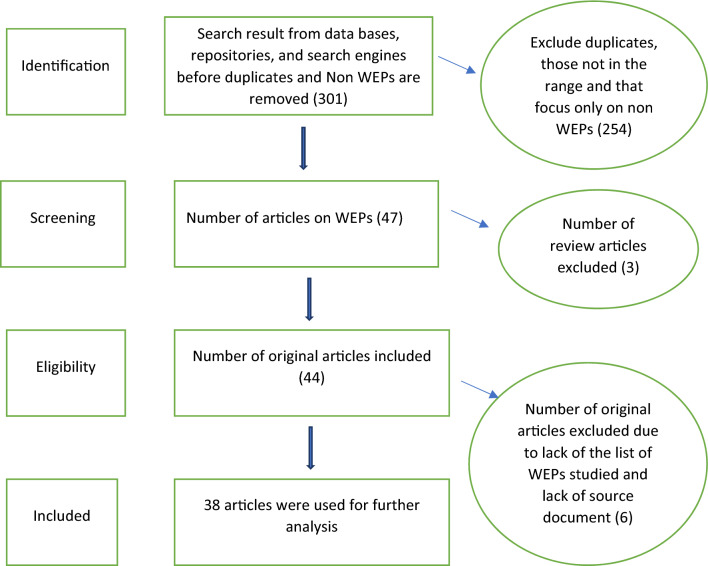
Database search of publications for review.

To determine the relevance of the articles in providing information about WEPs in Ethiopia, data fields were extracted from the identified studies. A total of 38 original studies focusing on WEPs in Ethiopia were thoroughly reviewed (Fig. 1). To ensure the accurate inclusion of WEPs that are truly present in the Ethiopian landscape, the verification of WEP species mentioned in the reviewed articles was done using reputable sources, such as the World Flora Online, the POWO database managed by the Royal Botanical Gardens-Kew, and the Flora of Ethiopia, and Eritrea (volumes 1 to 8). By utilizing these authoritative references, the research team was able to confirm the valid identification of the WEP species reported in the literature.

### Taxonomic diversity of WEPs

To assess the diversity of WEPs in Ethiopia, the distribution of species across families was analyzed. This was done by plotting the species' presence against the different plant families. Additionally, the total species (%) was plotted against the number of families to understand the contribution of families to the total species pool.

### Edible plant parts of WEPs

The comprehensive analysis of the total WEP species pool was categorized into six distinct groups based on the edible parts documented: seeds or grains, leaves and leafy shoots, flowers, fleshy fruits, underground parts, and other parts^7^. The establishment of a sixth heterogeneous group, referred to as "other parts," encompassed plant components beyond the initial five categories, such as the aril, bark, cambium, peduncle, sap, bulbil, fruit body, petiole, pith, and more. This categorization allowed for the determination of the utilization of different plant parts.

### Use frequency of WEPs

The relative frequency of citations (RFC) for each recorded WEP species was determined using the established methods^76^. To evaluate the popularity of a species across the study areas, a modified RFC was calculated for each species^77,78^. The RFC is calculated as RFC = FC/N, where FC represents the number of literature sources mentioning the species, and N is the total number of literature sources consulted (N = 38 in this study). This index ranges from 0 to 1, with 0 indicating that no study mentioned the plant as useful, and 1 indicating that all studies are likely to mention the use of the species.

### Relative use value of WEPs

To assess the significance of the edible parts of selected WEP species, the relative use value (RUV) was calculated. The RUV was determined for species that had three or more reported edible parts. The RUV index was computed by dividing the total number of useful parts (P) of a species by the total types of plant parts (T = six in this study). Mathematically, the RUV is calculated as RUV = P/T.

### Prioritization of WEPs

To shortlist and prioritize the WEP species identified in the literature, the research team utilized two key indices: the RFC, and RUV^77^. However, the team acknowledged that ethnobotanical indices have faced criticism from scholars for not being developed by statisticians^79^. To address this limitation and ensure a more comprehensive prioritization of WEPs, the team decided to consider additional criteria beyond just edibility. These additional factors include:

1. Proximate and mineral composition: The nutritional value of the WEPs, including their macronutrient and micronutrient profiles, will be considered.
2. Nutraceutical uses: The potential health benefits and medicinal properties of the WEPs will be evaluated.
3. Multipurpose applications: The versatility of the WEPs, in terms of their various uses (e.g. food, medicine, materials), will be considered.

By combining the information captured in the RFC and RUV indices with these additional criteria, the research team has been able to scientifically prioritize a diverse range of food plants that hold promise for sustainable cultivation and promotion. This holistic approach will provide a sustainable and nutritious food source for the large and diverse population of Ethiopia.

## Results and discussion

### Regional disparities in WEP research in Ethiopia

**Figure 2:**
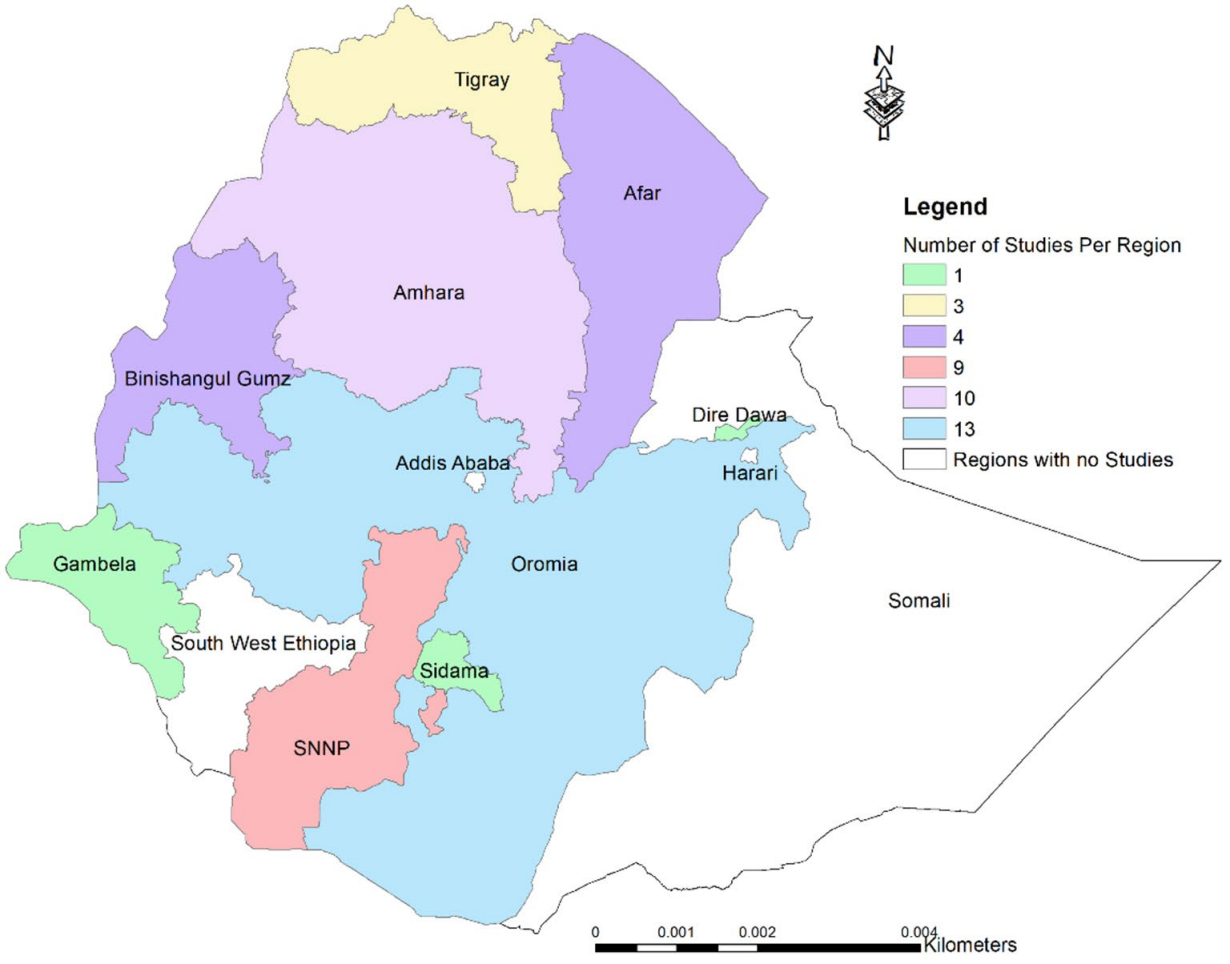
Map showing Ethiopian regions explored for ethnobotany of WEPs.

Ethiopia, with its 11 regional states and 2 chartered cities, has seen significant regional disparities in the study of WEPs. Certain areas have received far more attention than others in ethnobotanical research, while others have been largely overlooked. The Oromia, Amhara, and Southern Nations and Nationalities People's regional states have been the primary focus with 13, 10, and 9 ethnobotanical studies on WEPs, respectively (Fig. 2). In contrast, no such investigations were identified in the Harari, Somali, Southwest Ethiopia, and Addis Ababa city regions. The lack of studies in Addis Ababa and Harari can be attributed to the limited plant diversity in these urbanized areas. Despite the Somali region being the second largest, much of it consists of barren land unsuitable for plant growth. Conversely, Southwest Ethiopia is renowned for its rich ethnic, cultural, and plant diversity, underscoring the need for more ethnobotanical research in this underexplored region.

The research conducted so far has only covered a small fraction of Ethiopia’s landscape – just 65 out of the approximately 770 rural and urban areas, representing only 8.4% coverage. While an improvement from the 5% coverage reported earlier^45^, this figure still represents a tiny proportion given the country’s significant agroclimatic, ethnic, and cultural diversity. Thus, while progress has been made, the coverage of WEP research in Ethiopia remains limited, with significant regional disparities that need further studies.

### Regional trends in WEP studies

Analyzing the distribution of WEP studies across different regions reveals some interesting patterns. The southern, northwest, and central regions have received the most attention, with 12, 8, and 7 studies, respectively (Table 1). In contrast, only one study has been conducted in the southwest, and two each in the west, northeast, and eastern regions. The southern region not only had the highest number of studies but also the greatest average number of WEP species (59.75), likely due to its rich ethnic, cultural, and plant diversity^80,81^. The northwest (8 studies, 38.88 WEPs on average) and central regions (7 studies, 42.86 WEPs on average), while having fewer studies, exhibited higher average WEP species, likely reflecting the diverse plant life in these areas. The significant regional disparities in the WEP studies showed the need for further exploration, particularly in underrepresented areas like Southwest Ethiopia to gain a more comprehensive understanding of the country’s WEP resources.

**Table 1:** Broad geographic regions, regional states, and districts of the reviewed articles.

Broad geographic region in Ethiopia	Regional states	Districts	References
North‒West	Amhara	Sedie Muja	^82^
Central	Oromia	Bereket	^42^
North‒East	Amhara	Guba Lafto, Habru, Gedan and Dalanta Dawent	^83^
North‒West	Amhara	Yilmana Densa and Quarit	^84^
North; North‒West; West; South‒East; North; South	Amhara, Benishangul Gumuz, Gambella, SNNP, and Tigray	Kobo, Bambasi, Debate, Homosha, Mandura, Gog, Lare, Delo menna, Hammer and Raya Azebo	^85^
South	Oromia	Adola	^86^
North‒West	Amhara	Baso Liben and Debre Elias	^87^
North	Tigray	Hintalo-Wejrat, Alaje & Raya-Azebo	^88^
West	Oromia	Nole kaba	^89^
North‒West	Amhara	Quara	^90^
North‒West	Benishangul Gumuz	Bullen	^91^
South‒West	Oromia	Yayu, Doreni, Chora, Hurumu, Alge Sachi and Nopha	^92^
North‒West	Benishangul Gumuz	Bullen	^93^
South	SNNP & Oromia	Amaro and Gelana	^94^
West	Benishangul Gumuz	Kamash	^95^
East	Dire Dawa	Dire Dawa	^96^
North	Tigray	Raya Azebo, Raya Alamata, Ofla, Hawzien, Kolla Tembien, Laelay Maichew and Medebay Zana	^97^
East	Afar	Yalo	^98^
Central	Oromia	Jibat, Chelia and Dendi	^99^
South	Oromia	Dugda Dawa	^100^
South	SNNP	Burji	^101^
South	Oromia	Bule hora	^102^
Central	Oromia and Afar	Awash Fantale and Fantale	^103^
North-East	Afar	Aba'ala	^104^
South	SNNP	Amaro Special District and Arba Minch Zuria	^105^
Central	Oromia	Guna, Tiyo and Sire	^106^
South	Sidama	Hula	^107^
Central	Amhara	Berehet	^108^
North	Amhara	Delanta	^109^
South	SNNP	Maale and Debub Ari	^110^
North-West	Amhara	Chilga	^111^
Central	Afar and Oromia	Awash Fentale and Fentale	^112^
South	SNNP	Hamer	^113^
South	SNNP	Konso	^114^
South	SNNP	Konso	^115^
South	SNNP	Benna Tsemay	^116^
Central	Oromia	Fantalle and Boosat	^117^
North-West	Amhara	Libo Kemkem	^118^

*SNNPs* Southern Nations, Nationalities and Peoples.

### Taxonomic diversity of WEPs

This review has identified a total of 520 WEP species belonging to 94 families and 343 genera at the species and subspecies levels (Supplementary File 1). The plant families with the highest number of WEP species were Fabaceae (51 species, 9.81%), Rubiaceae (25 species, 4.81%), Anacardiaceae (21 species, 4.04%), Lamiaceae (20 species, 3.85%), and Moraceae (19 species, 3.66%). Families with more than twelve species are represented (Table 2).

**Table 2:** Distribution of species across families.

Families	Number of species	Families	Number of species	Families	Number of species	Families	Number of species
Fabaceae	51	Araceae	6	Zingiberaceae	3	Clusiaceae	1
Rubiaceae	25	Boraginaceae	6	Asphodelaceae	2	Costaceae	1
Anacardiaceae	21	Dioscoreaceae	6	Balanitaceae	2	Dennstaedtiaceae	1
Lamiaceae	20	Cyperaceae	5	Campanulaceae	2	Erythroxylaceae	1
Moraceae	19	Ebenaceae	5	Cannabaceae	2	Lauraceae	1
Cucurbitaceae	17	Olacaceae	5	Crassulaceae	2	Loranthaceae	1
Apocynaceae	16	Polygonaceae	5	Icacinaceae	2	Melastomataceae	1
Amaranthaceae	15	Sapotaceae	5	Iridaceae	2	Menispermaceae	1
Malvaceae	15	Annonaceae	4	Liliaceae	2	Molluginaceae	1
Acanthaceae	14	Asparagaceae	4	Loganiaceae	2	Myricaceae	1
Tiliaceae	13	Celastraceae	4	Moringaceae	2	Nyctaginaceae	1
Asteraceae	12	Combretaceae	4	Passifloraceae	2	Nymphaeaceae	1
Brassicaceae	12	Meliaceae	4	Podocarpaceae	2	Ochnaceae	1
Burseraceae	12	Myrtaceae	4	Portulacaceae	2	Oliniaceae	1
Capparidaceae	12	Oxalidaceae	4	Salicaceae	2	Papaveraceae	1
Poaceae	12	Phyllanthaceae	4	Salvadoraceae	2	Piperaceae	1
Rutaceae	12	Sapindaceae	4	Typhaceae	2	Pittosporaceae	1
Solanaceae	12	Urticaceae	4	Amaryllidaceae	1	Plumbaginaceae	1
Rhamnaceae	8	Verbenaceae	4	Aquifoliaceae	1	Polygalaceae	1
Vitaceae	8	Apiaceae	3	Aristolochiaceae	1	Resedaceae	1
Commelinaceae	7	Arecaceae	3	Bignoniaceae	1	Santalaceae	1
Convolvulaceae	7	Flacourtiaceae	3	Cactaceae	1	Simaroubaceae	1
Euphorbiaceae	7	Musaceae	3	Cleomaceae	1	Thymelaeaceae	1
Rosaceae	7	Myrsinaceae	3				

A previous review of WEPs in Ethiopia highlighted the extensive distribution of the Fabaceae family, which holds the highest number of species in the Ethiopian Flora^45^. This abundance may account for the widespread use of WEPs within this family. The dominance of Fabaceae as a source of edible plants has been documented in other countries as well. In a global review, Fabaceae is one of the most diverse families, with 625 edible species^119^. Studies in Indonesia^120^, Myanmar^121^, and India^77^ have also reported Fabaceae as a prevalent family of edible plants. The prevalence of Fabaceae-derived WEPs in Ethiopia underscores the significance of this plant family as an important food resource.

### Comprehensive inventory of WEPs

The current review, combined with the previous study^45^, has documented a comprehensive inventory of WEPs utilized in Ethiopia. Accordingly, a combined total of 281 WEPs were documented in both this review and the previous study^45^. Additionally, this review exclusively identified 239 more WEPs. Meanwhile, the previous study had solely reported 131 WEPs^45^. In total, the combined review has documented 651 WEPs utilized in Ethiopia (as detailed in Supplementary Files 1 and 2). The 651 WEPs documented in Ethiopia surpass the 615 WEPs identified for food consumption across five countries (Botswana, Kenya, Mali, South Africa, and Mexico)^122^. Similarly, a review in Morocco compiled a list of 246 WEPs utilized as food^123^. These comparisons highlight the remarkable wealth of WEP biodiversity in Ethiopia, as well as the growing body of ethnobotanical research in the country documenting traditional knowledge and uses of these valuable plant resources.

### The concentration of diversity of WEP species among families

**Figure 3:**
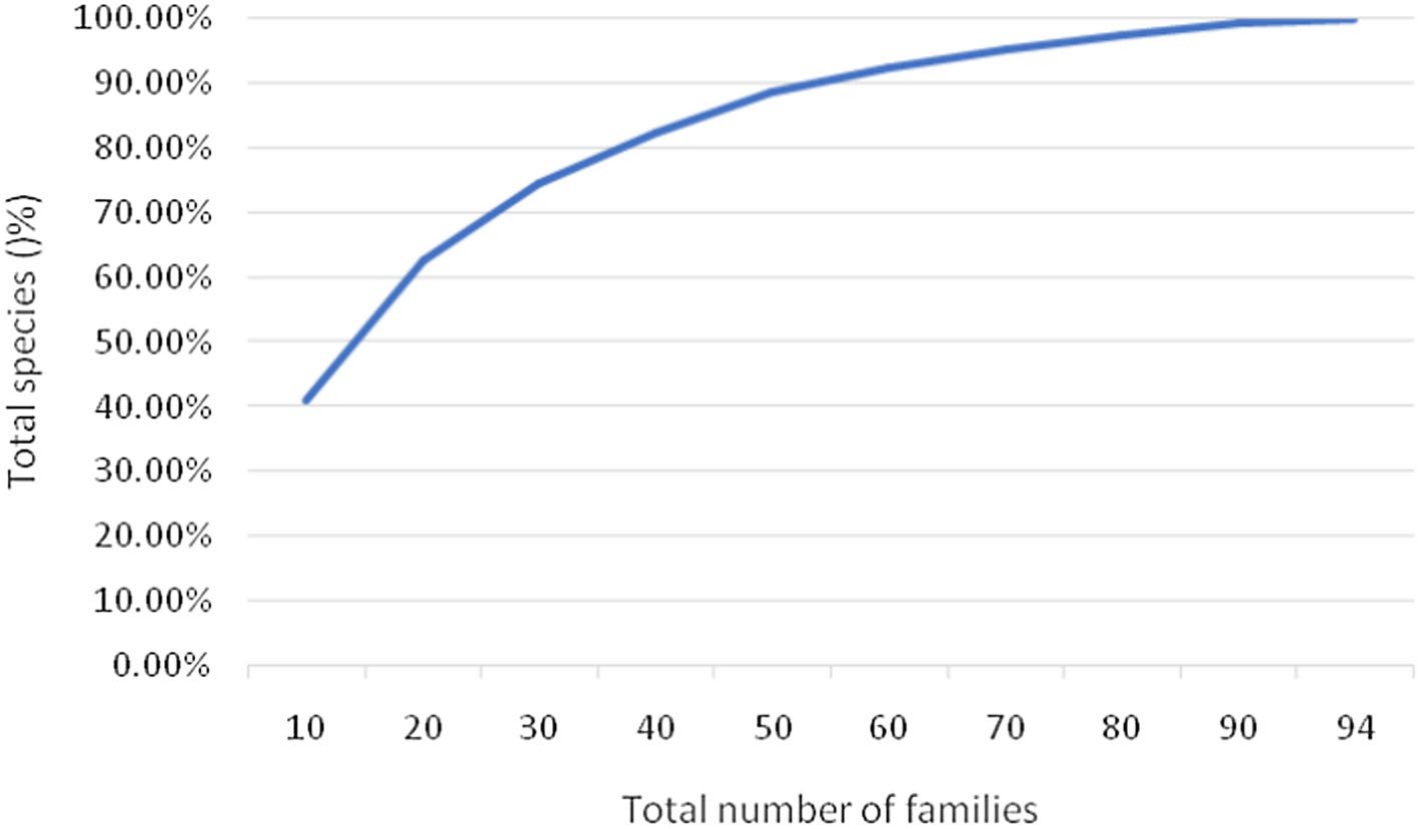
Contribution of families to the total species pool (%).

The top ten families, accounting for 10.64% of all families, were responsible for 40% of the total WEP species. While the top 10 families dominate, the diversity is skewed, with the first 50 families (53.2% of the total) contributing to 88% of the total WEP species (Fig. 3 and Supplementary File 1). This indicates that a relatively small proportion of plant families host the majority of the documented WEP diversity in Ethiopia. The uneven distribution of WEP diversity across plant families has important implications for conservation prioritization. Targeted efforts to preserve the top contributing families could help safeguard a significant proportion of the overall WEP diversity.

### Habits of WEPs

**Figure 4:**
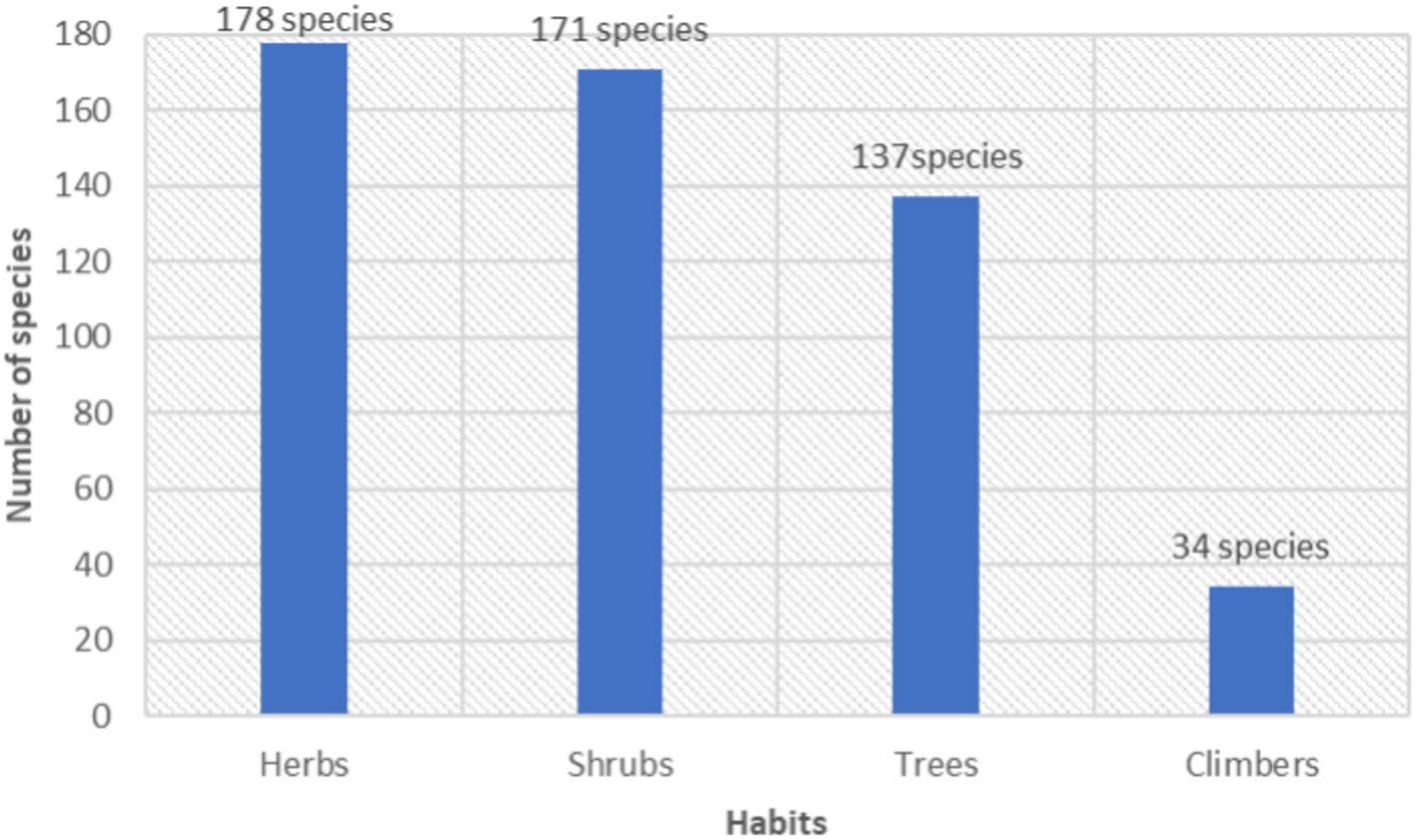
Habits of WEPs.

An analysis of the growth habits of the documented WEPs in Ethiopia reveals that the majority of WEPs were herbs, comprising 178 species (34.23%). Shrubs were the second most common, with 171 species (32.88%) (Fig. 4). This contrasts with a previous country-wide review^45^, which had identified shrubs as the most common, followed by trees, herbs, and climbers. The increase in the utilization of herbs in the current study may be attributed to the expanded scope of ethnobotanical investigations among diverse cultural and ethnic groups in Ethiopia. These groups appear to rely more heavily on herbs as a source of edible foods. This suggests that the consumption habits of WEPs may vary depending on cultural practices and traditions.

### Edible parts of WEPs

**Figure 5:**
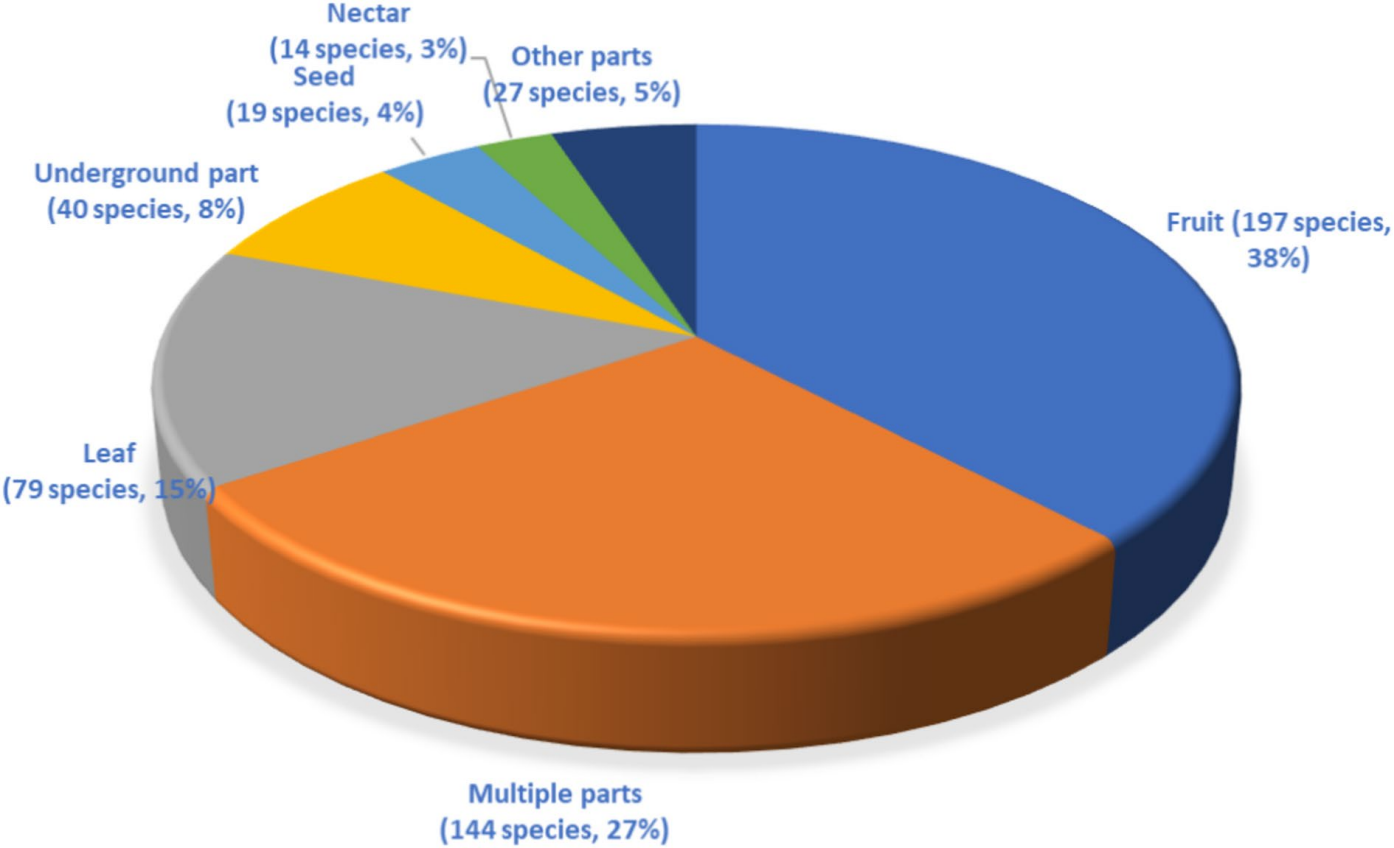
Edible plant parts.

The review of WEPs in Ethiopia reveals that various parts of these plants are consumed, including fruits, leaves, underground parts, seeds, nectars, stems, gums, bulbs, and bark. Among these, the less common parts such as bark, bulb, gum, and stem were categorized as ‘other parts’ due to their minimal consumption. The analysis shows that fruits were the most widely consumed edible part, making up 38% (197 species), followed by multiple parts (144 species, 27%), and leaves (79 species, 15%) (Fig. 5). This order of usable part consumption aligns with previous studies^45,124^, which also reported fruits to be the most widely consumed edible parts of WEPs. However, a review of WEPs in Italy^125^ reported that leaves were the most consumed plant parts. Additionally, the study^45^ noted that consuming multiple parts of WEPs was the least common in their review, whereas it was the second most common in the current review. The increasing inclusion of studies on various ethnic groups utilizing different plant components as food sources may explain the rise in the consumption of multiple parts of WEPs.

### Family-wise utilization of WEPs

**Figure 6:**
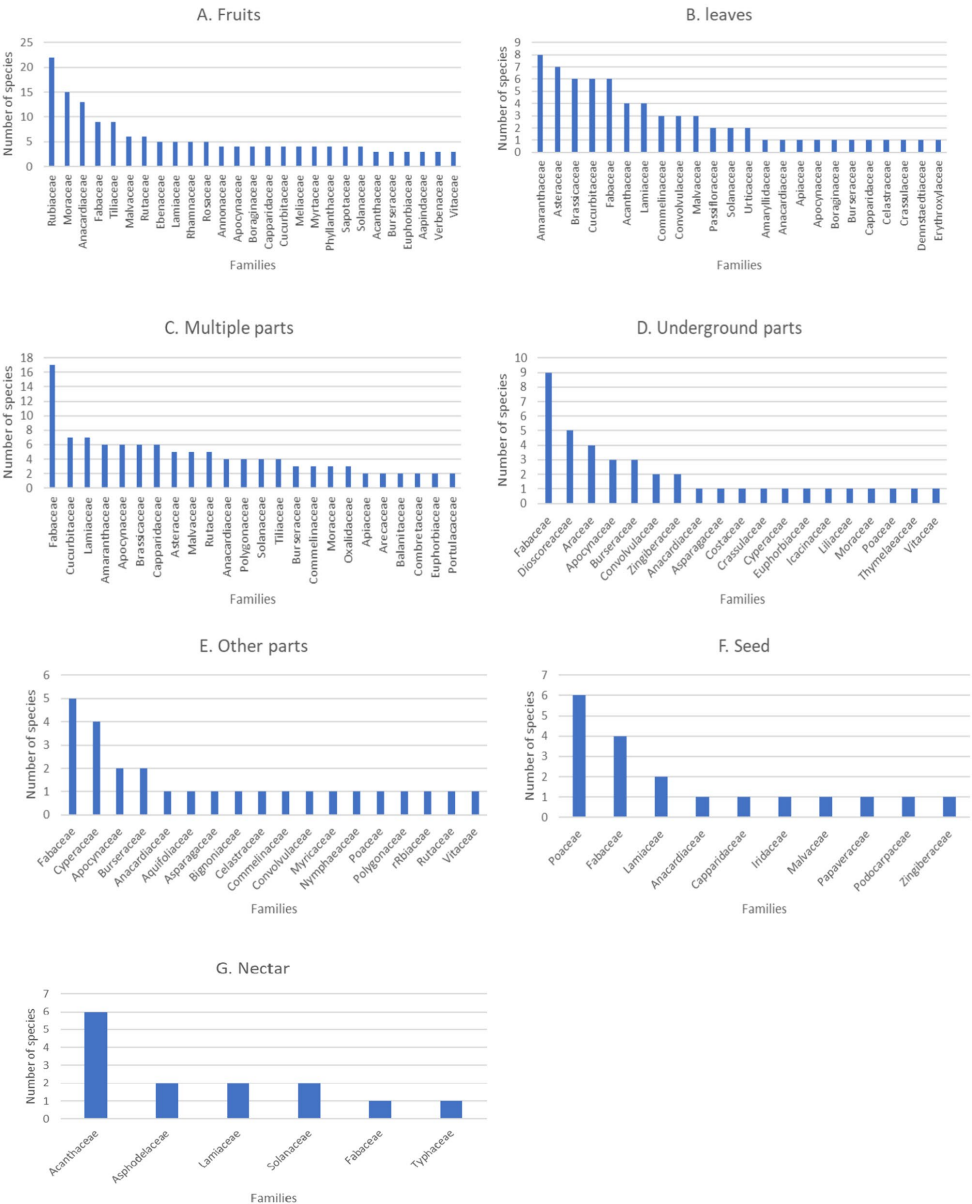
Distribution of species across families based on individual edible plant parts: (**A**) fruits, (**B**) leaves, (**C**) multiple parts, (**D**) underground parts, (**E**) other parts, (**F**) seeds, and (**G**) nectar.

The examination of WEP usage at the family level in Ethiopia reveals some notable trends. Fabaceae emerged as the predominant food plant family, ranking at the top in the categories of multiple parts (17 species), underground parts (9 species), and other parts (5 species). Additionally, this family ranked second in seed (4 species), fourth in fruit (9 species), fifth in leaf (6 species), and nectar (1 species). Other notable families that led in various edible categories include Rubiaceae (22 species in fruit), Amaranthaceae (8 species in leaf), Acanthaceae (6 species in nectar), and Poaceae (6 species in seed) (Fig. 6A–G). The predominance of Fabaceae members in plant usage has been a consistent finding in various reviews conducted in Asian countries as well^6,77^. The high species diversity of Fabaceae in tropical regions, such as Ethiopia, could be a key factor in its dominance^77^.

### WEPs sold in local markets

The present review highlighted that 20 of the reviewed studies on WEPs in Ethiopia included a market survey to evaluate the WEPs sold in local markets in their studies. The findings indicated that 82 WEPs were sold in local and surrounding district markets, providing a supplementary source of income for households. The most frequently cited WEPs sold in local markets were *Balanites aegyptiaca, Ximenia americana*, *Balanites rotundifolia*, *Tamarindus indica*, and *Ziziphus spina-christi* (Supplementary File 3).

In agreement with this report, studies from various regions around the world have shown that WFPs are not only harvested for subsistence but are also gathered in surplus quantities to generate income^126^. These WEPs sold in local markets underline the importance of WEPs in contributing to local economies and supporting the livelihoods of individuals who rely on these plants for income generation. Further research in this area can provide valuable insights into the market dynamics of WEPs and their potential for sustainable economic development in Ethiopia.

### Prioritization of WEPs using ethnobotanical indices

#### Use frequency of WEPs

The Relative Frequency of Citations (RFCs) values were used to categorize WEP species based on their citations in the reviewed ethnobotanical studies. The top five most common WEP species were *Carissa spinarum* (RFC: 0.89), *Cordia africana* (RFC: 0.76), *Ficus sur* (RFC: 0.74), *Balanites aegyptiaca* (RFC: 0.71), and *Ximenia americana* (RFC: 0.68) (Table 3). Out of the 520 WEP species documented in the 38 studies analyzed, over half (51.92%, 270 species) were recognized as edible in at least two studies (Supplementary File 1). This illustrates shared indigenous and local knowledge regarding the utilization of WEPs among the diverse cultural groups in the country. Moreover, it highlights that the diverse cultural traditions within these communities did not hinder the exchange of knowledge concerning the use of similar WEPs. The high citation frequencies of certain WEP species, such as *Carissa spinarum*, *Cordia africana*, and *Ficus sur*, suggest their widespread use across different regions and communities in the country. This information can be valuable for further research, conservation efforts, and promoting the sustainable use of these important wild food resources.

**Table 3:** Prioritized WEPs based on their RFC, and RUV (n = 38).

Species	FC	RFC	NoEPs	RUV	Species	FC	RFC	NoEPs	RUV
*Carissa spinarum*	34	0.89	2	0.33	*Nasturtium officinale*	1	0.03	5	0.83
*Cordia africana*	29	0.76	1	0.17	*Oxalis latifolia*	1	0.03	5	0.83
*Ficus sur*	28	0.74	1	0.17	*Thymus serrulatus*	1	0.03	5	0.83
*Balanites aegyptiaca*	27	0.71	4	0.67	*Brassica rapa*	2	0.05	4	0.67
*Ximenia americana*	26	0.68	1	0.17	*Embelia schimperi*	11	0.29	4	0.67
*Ficus sycomorus*	25	0.66	2	0.33	*Lannea rivae*	3	0.08	4	0.67
*Ficus vasta*	24	0.63	3	0.5	*Oxalis stricta*	1	0.03	4	0.67
*Opuntia ficus-indica*	23	0.61	2	0.33	*Pachycymbium laticoronum*	2	0.05	4	0.67
*Tamarindus indica*	21	0.55	3	0.5	*Piliostigma thonningii*	7	0.18	4	0.67
*Ziziphus spina-christi*	20	0.53	1	0.17	*Rhamnus prinoides*	4	0.11	4	0.67
*Syzygium guineense*	15	0.39	1	0.17	*Rumex nervosus*	16	0.42	4	0.67

*FC* frequency of citation, *RFC* relative frequency of citations, *NoEPs* number of edible parts, *RUV* relative use value.

#### Use value of WEPs

The relative use values (RUVs) of the WEP species examined in this study showed significant variation, ranging from 0.17 to 0.83. Notably, only three species, *Nasturtium officinale*, *Oxalis latifolia*, and *Thymus serrulatus* exhibited RUV values of 0.83 each (Table 3). Following closely behind were nine species, including *Balanites aegyptiaca*, *Brassica rapa*, *Embelia schimperi*, *Lannea rivae*, and *Oxalis stricta*, each with RUV values of 0.67. Furthermore, there were 34 species, such as *Ficus vasta, Tamarindus indica*, *Acacia tortilis*, *Amaranthus hybridus*, *Asparagus africanus*, and *Balanites rotundifolia*, with RUV of 0.5. Additionally, 98 species were found to have an RUV of 0.33. The majority of the remaining 376 species (72.31%) were identified as having only one edible part, resulting in an RUV of 0.17 (Supplementary File 1). Overall, these findings shed light on the varying degrees of importance and utilization of different plant species within the country.

### Prioritizing WEPs through multiple criteria

The study used a multi-criteria approach to prioritize the most important WEP species in Ethiopia. The prioritization was based on evaluating the WEPs against several criteria, including the ethnobotanical indices calculated in the review. Through this process, nine WEP species were selected as top priorities for potential cultivation and promotion. These prioritized WEPs were *Balanites aegyptiaca, Ximenia americana, Tamarindus indica, Ziziphus spina-christi, Carissa spinarum, Cordia africana, Ficus sycomorus, Ficus sur,* and *Syzygium guineense* (Table 3). These species emerged as the most important based on a combination of factors besides ethnobotanical indices (Table 4).

**Table 4:** Prioritization of wild edible plants through multiple criteria.

WEP	Nutraceutical	Proximate composition	Mineral content	Sold in local markets	Multipurpose uses
*Carissa spinarum*	Gonorrhea^105^	Energy^127^	Ca, Fe, Zn^127^	^90,97^	Medicine, construction, furniture, agricultural tools fuel wood, fodder, fence^105,128^
*Cordia africana*	Diarrhea^105^	Carbohydrate, crude ash, energy^129^	Ca, K, Fe^129^	^97^	Medicine, construction, furniture, agricultural tools fuel wood, fodder, fence^105,130,131^
*Ficus sur* Forssk	Toothache^132^	Carbohydrate, energy, ash^133^	Fe, Zn, K, Ca^133^	^90^	Medicinal, wild edible, fodder, fuel wood^134^
*Balanites aegyptiaca*	Abdominal pain and Snake bite^105^	Ash, crude protein, crude fiber, carbohydrate^135^	Ca, Fe, Mg^135^	^97^	Medicine, construction, furniture, agricultural tools fuel wood, fodder, fence^105,136^
*Ximenia americana*	Abdominal pain^105,137,138^	Crude fat, crude protein, fiber^139^	Ca, Mg^139^	^90,97,112^	Medicine, construction, furniture, agricultural tools fuel wood, fodder, fence^105^
*Tamarindus indica*	Diarrhea^105,140^	Ash, crude lipid, crude protein^141^	K, Mg, Fe^141^	^90,97,112^	Medicine, construction, furniture, agricultural tools fuel wood, fodder, fence^105^
*Ficus sycomorus*	Hepatitis^105^	Carbohydrate, energy^142^	Zn, Fe^142^	^112^	Medicine, construction, furniture, agricultural tools fuel wood, fodder, fence^105^
*Ziziphus spina-christi*	Dandruff^105^	Ash, carbohydrate, energy^143^	Ca, Fe, K, P^143^	^90,97,112^	Medicine, construction, furniture, agricultural tools fuel wood, fodder, fence^105,144^
*Syzygium guineense*	Diarrhea^132^	Carbohydrate, energy^142^	Zn, Fe^142^	^90,97,110^	Medicine, construction, furniture, agricultural tools fuel wood, fodder, fence^105^

Prioritizing these nine WEP species provides a strategic framework to focus research, conservation, and development efforts on the wild food resources that are most valued and utilized by local communities in Ethiopia. Promoting the cultivation and sustainable use of these priority WEPs can help enhance food security, support livelihoods, and preserve indigenous knowledge and practices related to wild edible plants in the country.

### Threats to WEPs and traditional management practices

WEPs have the potential to enhance food security and support poverty reduction strategies globally. However, recent global reports have highlighted the rapid disappearance of this valuable plant diversity due to various threats, including land use changes, habitat destruction, deforestation, overharvesting, agricultural change, and loss of traditional management practices^39,40,119,145^.

**Figure 7:**
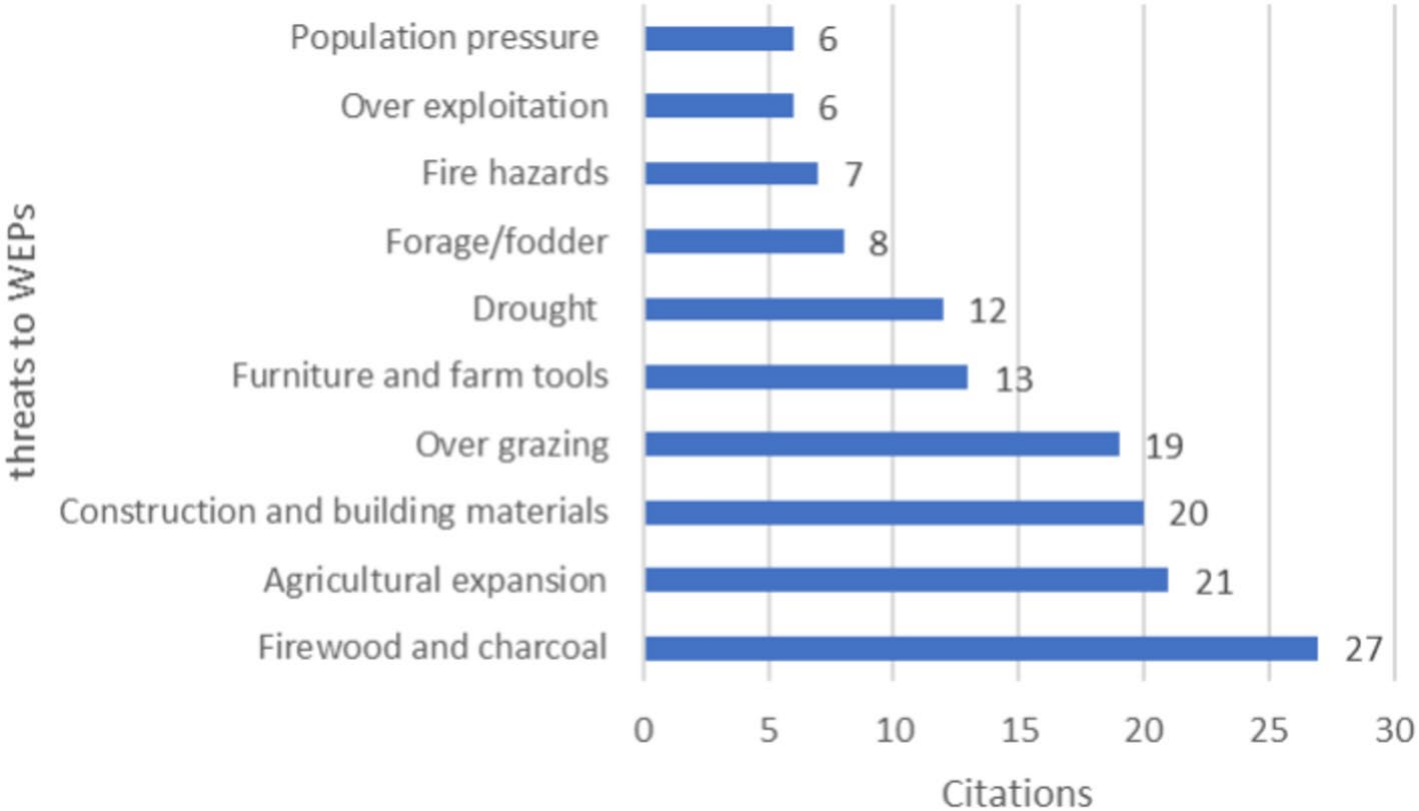
Major threats to WEPs.

In Ethiopia, the challenges impacting the distribution of WEPs vary depending on factors such as agroecology, culture, norms, and population pressure. Despite these regional variations, a review of several studies^84,90,93,94,112,116^ have identified common threats to WEPs across the country. The findings indicate that certain threats consistently impact WEPs, including collection for firewood and charcoal (27), agricultural expansion (21), use for construction and building materials (20), and overgrazing (19) as the most frequently cited threats to the WEPs in Ethiopia (Fig. 7).

The threats facing WEPs are closely linked to increasing population pressures, as highlighted in the World's Report on Biodiversity for Food and Agriculture^40^. This has led to a significant rise in population size, resulting in the expansion and intensification of land use, overexploitation of biological resources, utilization of marginal lands, and the breakdown of traditional resource management systems. These threats emphasize the urgent need for the conservation of WEPs before they are lost.

Conserving WEPs is essential, whether done in their natural habitat (in situ) or in controlled environments (ex-situ). The preservation of WEPs is just as important as that of any other plant species. It is vital to have collaborative efforts among various institutions, including universities, research centers, biodiversity conservation institutes, and other stakeholders^124^. Community participation is also key in effectively addressing the challenges associated with WEP conservation.

**Figure 8:**
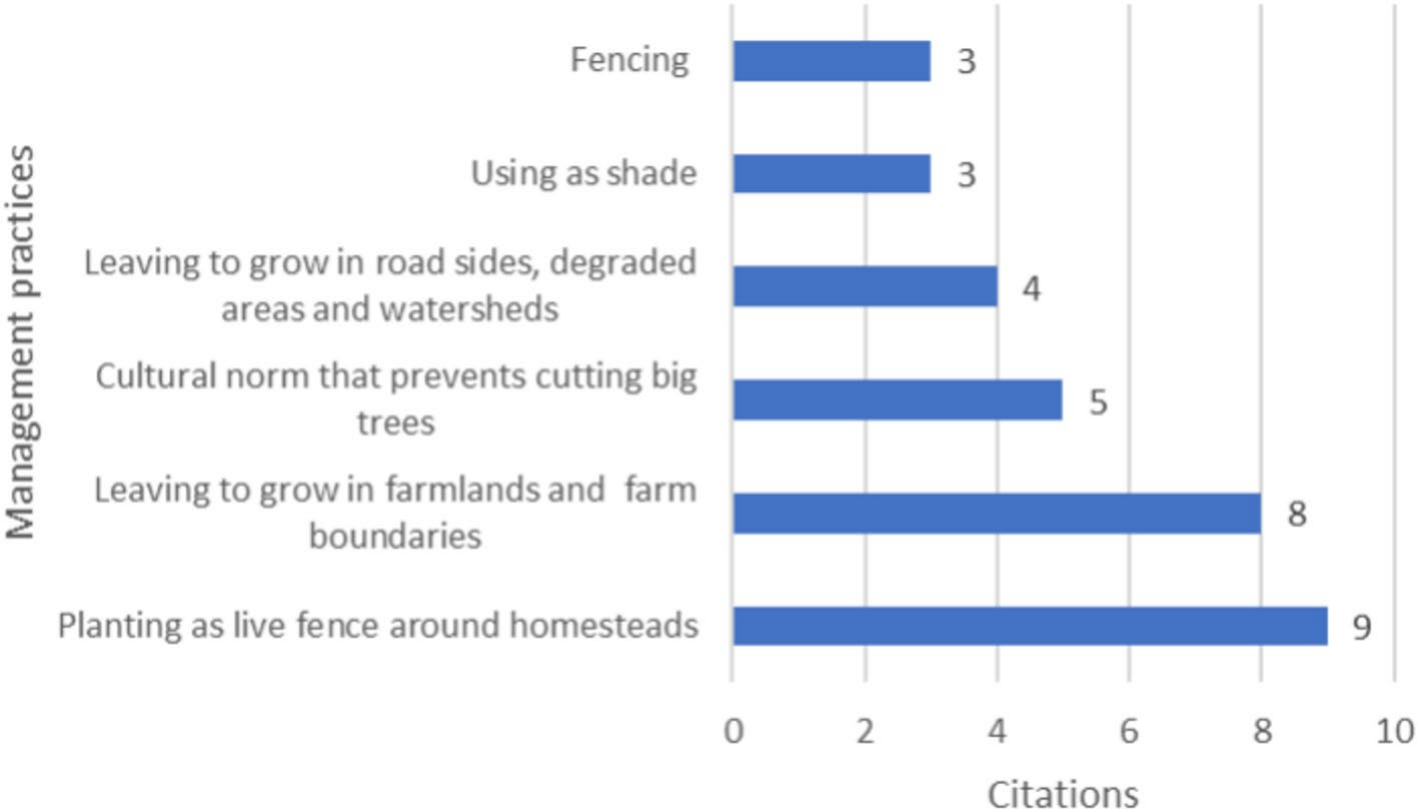
Traditional management practices of WEPs.

Despite the threats facing WEPs in the country, recent studies have identified various traditional management practices that are being utilized by communities^42,89,101,103,109^. The most prevalent traditional management practices include planting seedlings of WEPs as live fences around homesteads, allowing existing WEPs to thrive in farmlands and farm boundaries, and fostering cultural norms that discourage the cutting of large trees (Fig. 8). Not only do these practices contribute to the preservation of WEPs, but they also promote biodiversity and ecosystem health in the country.

A potential strategy for conserving WEPs could involve revitalizing cultural practices through the promotion of informal cultivation or moderate management of homesteads, fringes, pastures, or fallow lands^146^. By implementing these methods, it is possible to safeguard key WEPs from the threats posed by population growth and the increasing demands on grazing areas, farmlands, and forests. This approach not only helps to preserve biodiversity but also ensures the sustainable use of natural resources for future generations.

## Conclusion

This systematic review comprehensively examined the diversity, utilization, and potential contribution of WEPs to food security in Ethiopia. The study identified 651 WEP species from 94 families across the country, although this number is likely an underestimate due to limited research coverage. This underscores the importance of conducting further ethnobotanical studies to fully explore and understand the extent of WEP diversity and utilization throughout Ethiopia.

The diversity of WEPs in Ethiopia, including trees, shrubs, herbs, and climbers highlighting their significant role in supporting food systems. Different parts of these WEPs, such as leaves, fruits, flowers, seeds, underground organs, and other components are consumed. However, these resources face various threats, and current management practices are inadequate, necessitating enhanced conservation efforts.

Nine prioritized WEPs with high potential for cultivation and promotion have been identified, demanding the attention of researchers, policymakers, and local peoples to leverage their capacity for improving food and nutritional security. Future research should further explore the economic value of WEPs, including income generation from their sales, as well as integrate multidisciplinary perspectives including taxonomic, phylogenetic, biogeographic, and ethnobotanical information to provide a more holistic understanding of these important resources.

### Supplementary Information

Supplementary Information.

## Data Availability

The datasets generated during and/or analyzed during the current study are available from the corresponding author on reasonable request.
