# Supplementary material for: A systematic review exploring the diversity and food security potential of wild edible plants in Ethiopia
Source: Sci Rep. 2024 Aug 1;14:17821. doi: 10.1038/s41598-024-67421-y (PMC11294628; doi:10.1038/s41598-024-67421-y)
Supplement: Supplementary file 1 — Supplementary Information. [file 41598_2024_67421_MOESM1_ESM.docx]

**A Systematic Review Exploring the Diversity and Food Security Potential of Wild Edible Plants in Ethiopia**

**Supplementary files**

**Supplementary file 1** List of reviewed wild edible plants of Ethiopia between 2011 and 2022

Abbreviations and Symbols **Local names**: A-Amharic, Af-Afar, Ar-Ari, B-Burji, G-Gumuz, K-Konso, Ko-Koorete, M-Maale, O-Affan Oromo, So-Somali, S-Sidama, Sh-Shinasha, T-Tigrigna. **FC**: Frequency of citation; **RFC**: Relative frequency of citation; **RUV**: Relative use value

* Refer to those WEPs reviewed in the current study

** Refer to those WEPs reviewed by both the current study and the previous study by Lulekal et al. (2011)

| **No** | **Scientific name** | **Family name** | **Local name** | **Habit** | **Parts used** | **FC** | **RFC** | **RUV** | **References** |
| --- | --- | --- | --- | --- | --- | --- | --- | --- | --- |
| 1 | *Abelmoschus esculentus* (L.) Moench. (Syn: *Hibiscus esculentus* L.) * | Malvaceae | Andedha (G), Bamia/Wayika (A) | Herb | Fruit | 4 | 0.11 | 0.17 | 94, 95, 123, 133 |
| 2 | *Abelmoschus ficulneus* (L.) weight & Arn* | Malvaceae | Andeha (G), Andhayiza (Sh) | Shrub/  Herb | Leaf, Fruit | 2 | 0.05 | 0.33 | 94, 123 |
| 3 | *Acacia abyssinica* Hochst. ex Benth. ** | Fabaceae | Laftoo/Wachoadii (O), Grar/Bazragirar (A) | Tree | Other parts | 6 | 0.16 | 0.17 | 42, 93, 96, 115, 126, 138 |
| 4 | *Acacia albida* Del.** | Fabaceae | Bate girare (Ko) | Tree | Other parts | 1 | 0.03 | 0.17 | 93 |
| 5 | *Acacia brevispica* Harms* | Fabaceae | Hamaresa (O) | Shrub | Leaf | 1 | 0.03 | 0.17 | 92 |
| 6 | *Acacia etbaica* Schweinf. ** | Fabaceae | Kessele (A), Ajoo (O/Af), Wangayo (O), Sebunu (Ko) | Tree | Fruit, Other parts | 4 | 0.11 | 0.33 | 93, 99, 124, 132 |
| 7 | *Acacia hockii* De Wild. (Syn: *Vachellia hockii* (De Wild.) Seigler&Ebinger) ** | Fabaceae | Checheha/Chaqenti/Chehchehta (K), Lanqey (B), | Tree | Other parts | 4 | 0.11 | 0.17 | 98, 134, 135, 136 |
| 8 | *Acacia melanoxylon* R.Br. (Syn: *Racosperma melanoxylon* (R.Br.) Pedley) * | Fabaceae |  | Tree | Fruit | 1 | 0.03 | 0.17 | 117 |
| 9 | *Acacia mellifera* (Vahl) Benth. * | Fabaceae | Maka’arto/Ma’egherto (Af), SepeneGuro (O), Tsurura (Ko) | Shrub | Fruit, Other parts | 1 | 0.03 | 0.33 | 92, 93 |
| 10 | *Acacia negrii* Pic. Semi. ** | Fabaceae | Adachaa (O/Af), Garbi (O) | Tree | Other parts | 2 | 0.05 | 0.17 | 99, 126 |
| 11 | *Acacia nilotica* (L.) Willd. ex Del.** | Fabaceae | Kesel-e(-to) (Af); Burkuke (O) | Tree | Other parts | 1 | 0.03 | 0.17 | 92 |
| 12 | *Acacia nilotica* (L.) Willd. ex. Del. *subsp. indica* (Benth.) Brenan* | Fabaceae | keselto (Af) | Tree | Other parts | 1 | 0.03 | 0.17 | 129 |
| 13 | *Acacia oerfota* (Forssk.) Schweinf. * | Fabaceae | Gomerto (Af); Ajo (O), Wangayoo (O/Af) | Shrub | Fruit | 2 | 0.05 | 0.17 | 92, 99 |
| 14 | *Acacia polyacantha* Willd. ** | Fabaceae |  | Tree | Fruit | 1 | 0.03 | 0.17 | 102 |
| 15 | *Acacia prasinata* Hunde (Syn: *Vachellia prasinata* (Hunde) Kyal. &Boatwr.) * | Fabaceae | Dodoti (O) | Tree | Fruit | 1 | 0.03 | 0.17 | 137 |
| **No** | **Scientific name** | **Family name** | **Local name** | **Habit** | **Parts used** | **FC** | **RFC** | **RUV** | **References** |
| 16 | *Acacia senegal* (L.) Willd. ** | Fabaceae | Saphansaadi (O), Pohitata (K), Adado (Af); Sepensa Dima/(O), Dere (A), Chiewe (Ko) | Tree | Seed, Other parts | 7 | 0.18 | 0.33 | 92, 93, 99, 132, 134, 135, 137 |
| 17 | *Acacia seyal* Delile** | Fabaceae | Adigento/Makani (Af); Wachu/Waaccuadii/shema (O), Wacho/Nechgirar (A) | Tree | Other parts | 9 | 0.24 | 0.17 | 92, 93, 99, 115, 127, 128, 132, 134, 137 |
| 18 | *Acacia sieberiana var. woodii* (Burtt-Davy) Keay and Brenan** | Fabaceae | Nechgirar/Tedecha (A) | Tree | Other parts | 1 | 0.03 | 0.17 | 132 |
| 19 | *Acacia tortilis* (Forssk.) Hayne** | Fabaceae | Xadacha/Mutes/Dhadachaa (O), E’IBITO/A'abto/BEHBEY (Af), Tedecha/Dere (A) | Tree | Fruit, Seed, other parts | 6 | 0.16 | 0.5 | 92, 93, 127, 129, 132, 137 |
| 20 | *Acanthus eminens* C.B.Clarke* | Acanthaceae | Hoxoxa (B), Goorissa/ Goordhisa/Sakarroo (O) | Shrub | Flower | 3 | 0.08 | 0.17 | 98, 118, 127 |
| 21 | *Acanthus polystachius* Delile* | Acanthaceae | kosoruu (O), kusheshlia | Shrub | Flower | 2 | 0.05 | 0.17 | 115, 126 |
| 22 | *Acanthus pubescens* (Oliv.) Engl.* | Acanthaceae | Boha (G), Kosoru (O), Kosheshila (A) | Shrub | Flower | 3 | 0.08 | 0.17 | 100, 119, 123 |
| 23 | *Acanthus sennii* Chiov.** | Acanthaceae | Kushashile (A), Kosoruu/Sokorruu (O), Ankula (Ko), Koshosha (Sh) | Shrub | Flower, Fruit | 10 | 0.26 | 0.33 | 42, 93, 94, 96, 115, 119, 126, 132, 133, 138 |
| 24 | *Acokanthera schimperi* (A. DC.) Schweinf. ** | Apocynaceae | Lawa (K), Qaraarruu (O), Merenz (A), Qaraaro (S), Wato (Ko) | Shrub | Fruit | 7 | 0.18 | 0.17 | 93, 126, 127, 130, 131, 132, 135 |
| 25 | *Adansonia digitata* L.** | Malvaceae | Diza (A) | Tree | Fruit, Other parts | 2 | 0.05 | 0.33 | 95, 117 |
| 26 | *Adenia ellenbeckii* Harms** | Passifloraceae | Qaqula (K) | Herb | Leaf | 3 | 0.08 | 0.17 | 134, 135, 136 |
| 27 | *Adenia venenata* Forssk. ** | Passifloraceae | Nama (K) | Climber | Leaf | 1 | 0.03 | 0.17 | 135 |
| 28 | *Aeonium leucoblepharum* A. Rich. * | Crassulaceae | Yetota-kita (A) | Herb | Leaf | 1 | 0.03 | 0.17 | 97 |
| 29 | *Aframomum alboviolaceum* (Ridl.) K. schum** | Zingiberaceae | Ola (G) | Herb | Underground part | 1 | 0.03 | 0.17 | 123 |
| 30 | *Aframomum corrorima* (Braun) Jansen* | Zingiberaceae | Ogiyo (O) | Herb | Seed | 1 | 0.03 | 0.17 | 100 |
| 31 | *Afrocarpus falcatus* (Thunb.) | Podocarpaceae | Birbirsa (O), Zigba (A) | Tree | Seed | 1 | 0.03 | 0.17 | 130 |
| 32 | *Albizia schimperiana* Oliv. ** | Fabaceae | Garbii (O), Sessa (A) | Tree | Other parts | 2 | 0.05 | 0.17 | 115, 127 |
| 33 | *Allophylus abyssinicus* (Hochst) Radlk. ** | Sapindaceae | Xonxolooma (S), Irekemu (O), Saarajii/Imbis (A) | Tree | Fruit, Seed | 4 | 0.11 | 0.33 | 116, 127, 130, 131 |
| 34 | *Aloe camperi* Schweinf. * | Asphodelaceae | Wende-eret (A) | Shrub | Flower | 1 | 0.03 | 0.17 | 97 |
| 35 | *Alysicarpus glumaceus* (Vahl.) DC.* | Fabaceae | Singo (O) | Herb | Underground part | 1 | 0.03 | 0.17 | 127 |
| 36 | *Alysicarpus rugosus* (Wild.) DC.* | Fabaceae | Kiliccu (O) | Herb | Underground part | 1 | 0.03 | 0.17 | 127 |
| 37 | *Amaranthus angustifolius* Lam.* | Amaranthaceae | Rasuta (K) | Herb | Leaf | 1 | 0.03 | 0.17 | 135 |
| **No** | **Scientific name** | **Family name** | **Local name** | **Habit** | **Parts used** | **FC** | **RFC** | **RUV** | **References** |
| 38 | *Amaranthus caudatus* L.** | Amaranthaceae | Eyasu/Raafuu (O), Raso (B), Akechechile/Darka (G) | Herb | Seed, Leaf | 8 | 0.21 | 0.33 | 93, 94, 98, 102, 118, 123, 126, 128 |
| 40 | *Amaranthus dubius* Mart. ex Thell. ** | Amaranthaceae | Rasuta (K), Alema (A), Raaffu (O) | Herb | Leaf, Seed | 4 | 0.11 | 0.33 | 93, 127, 132, 136 |
| 39 | *Amaranthus cruentus* Thell* | Amaranthaceae | Lama (Sh) | Herb | Leaf, Seed | 2 | 0.05 | 0.33 | 94, 102 |
| 41 | *Amaranthus graecizans* L.** | Amaranthaceae | Rasuta (K) | Herb | Leaf | 3 | 0.08 | 0.17 | 122, 134, 136 |
| 42 | *Amaranthus hybridus* L.** | Amaranthaceae | Pasa (K), Aluma (A), Hamlitilian (T), Ekicanqila/Dahka (G) | Herb | Leaf, Seed, Other parts | 8 | 0.21 | 0.5 | 94, 97, 102, 104, 116, 123, 135, 136 |
| 43 | *Amaranthus spinosus* L.** | Amaranthaceae | Chichariya (G), Raaffu (O), Ferenjaluma (A) | Herb | Leaf, Fruit | 3 | 0.08 | 0.33 | 93, 116, 123 |
| 44 | *Amaranthus viridis* L.** | Amaranthaceae | Aluma (A) | Herb | Leaf | 1 | 0.03 | 0.17 | 116 |
| 45 | *Amorphophallus gallaensis* (Engl.) N. E. Br.** | Araceae | Qicu (O) | Herb | Fruit | 1 | 0.03 | 0.17 | 100 |
| 46 | *Amorphophallus gomboczianus* Pichi. Serm. ** | Araceae | Pakanna/Qachama (K), Laya (B) | Herb | Underground part | 3 | 0.08 | 0.17 | 98, 135, 136 |
| 47 | *Ampelocissus schiperiana* (Hochst. Ex A.Rich.) Planch. ** | Vitaceae | Antsiqina (G) | Shrub | Other parts | 1 | 0.03 | 0.17 | 123 |
| 48 | *Anethum graveolens* L.* | Apiaceae | Bishela/Lubicha (G) | Herb | Flower, Leaf | 2 | 0.05 | 0.33 | 94, 123 |
| 49 | *Annona cherimola* Mill. * | Annonaceae | Gishita (Sh) | Tree | Fruit | 1 | 0.03 | 0.17 | 94 |
| 50 | *Annona senegalensis* Pers.** | Annonaceae | Dangarasho (M), Bambuta (G/Sh) | Tree | Fruit | 3 | 0.08 | 0.17 | 94, 114, 123 |
| 51 | *Apodytes dimidiata* E.Mey. ex-Arn. * | Icacinaceae | Doongiicho (S), Manqeroo (O/Af) | Tree | Fruit, Other parts | 2 | 0.05 | 0.33 | 99, 131 |
| 52 | *Argemone mexicana* L.** | Papaveraceae |  | Herb | Seed | 1 | 0.03 | 0.17 | 135 |
| 53 | *Arisaema flavum* (Forssk.) Schott** | Araceae | Liltota/Litota (K), Qolcooma (S) | Herb | Leaf, Underground part | 3 | 0.08 | 0.33 | 131, 135, 136 |
| 54 | *Arisaema schimperianum* Schott** | Araceae | Hidha (B), Sise (Ko), Amoch (A) | Herb | Underground part | 3 | 0.08 | 0.17 | 93, 98, 119 |
| 55 | *Artemisia abyssinica* Sch. Bip. ex A. Rich. * | Asteraceae | Kodoo (O) | Herb | Leaf | 1 | 0.03 | 0.17 | 126 |
| 56 | *Arundinaria alpina* K.Schum. (Syn: *Yushania alpina* (K. Schum.) W.C.Lin)** | Poaceae | Shimala (O), Gerqeha (A), Leemmicho (S) | Tree | Leaf | 3 | 0.08 | 0.17 | 119, 126, 131 |
| 57 | *Asparagus africanus* Lam.** | Asparagaceae | Hinkarta/Hingarta (K), Hide sere/Seriti (O), Yesetkesit (A), Serity (Ko), Gaha (Sh) | Shrub | Leaf, Fruit, Underground part | 5 | 0.13 | 0.5 | 92, 93, 121, 132, 135 |
| 58 | *Asparagus scaberulus* A. Rich. ** | Asparagaceae | Erkakta (K) | Shrub | Fruit | 1 | 0.03 | 0.17 | 135 |
| 59 | *Asystasia gangetica* (L.) T. Anderson** | Acanthaceae | Atolleta (K), Darguwa (G) | Herb | Leaf | 3 | 0.08 | 0.17 | 123, 134, 135 |
| **No** | **Scientific name** | **Family name** | **Local name** | **Habit** | **Parts used** | **FC** | **RFC** | **RUV** | **References** |
| 60 | *Balanites aegyptiaca* (L.) Delile** | Balanitaceae | Baddanno/Badanaa/Baddanii (O), Hangalta/Hankalta (K), Bedeno (T, Ko) Udayito/Uda/Ala’ito (Af), Donkey (M), Angalda (B), Kudekuda/Jemo/Lalo/Asa masgeria (A), Qota (Sh) | Tree | Fruit, Leaf, Flower, Other parts | 27 | 0.71 | 0.67 | 91, 92, 93, 94, 95, 98, 99, 102, 104, 114, 116, 117, 118, 119, 120, 124, 125, 127, 128, 129, 130, 132, 133, 134, 135, 136, 137 |
| 61 | *Balanites rotundifolia* (Tiegh.) Blatt. ** | Balanitaceae | Kutata/Kuteta/Patana (K), Kuze (M), Alayaito (Af), Badanaokolee/Bedena (O) | Shrub | Fruit, Seed, Leaf | 10 | 0.26 | 0.5 | 91, 114, 117, 124, 125, 127, 129, 134, 135, 136 |
| 62 | *Barleria eranthemoides* R.Br.exC.B.Clark.** | Acanthaceae |  | Shrub | Flower | 1 | 0.03 | 0.17 | 132 |
| 63 | *Barleria longissima* Lindau** | Acanthaceae | Bichbichat (K) | Shrub | Flower | 1 | 0.03 | 0.17 | 135 |
| 64 | *Barleria quadrispina* Lindau* | Acanthaceae | Tsetselko (Ko) | Shrub | Fruit | 1 | 0.03 | 0.17 | 93 |
| 65 | *Becium grandiflorum* (Lam.) Pic. Serm. ** | Lamiaceae | Muatis/Metete (A), Tebeb (T) | Shrub | Flower | 2 | 0.05 | 0.17 | 97, 104 |
| 66 | *Berchemia discolor* (Klotzsch) Hemsl. ** | Rhamnaceae | Qananta (K), Jajjaba (O), Yyeybito/Katoya (Af); | Tree | Leaf, Fruit | 8 | 0.21 | 0.33 | 92, 93, 99, 129, 134, 135, 136, 137 |
| 67 | *Bidens macroptera* (Sch. Bip. ex Chiov.) Mesfin* | Asteraceae | Kello (O) | Herb | Leaf | 1 | 0.03 | 0.17 | 120 |
| 68 | *Bidens pachyloma* (Oliv. &Hiern.) Cufod. ** | Asteraceae | Kello (O) | Herb | Leaf | 1 | 0.03 | 0.17 | 126 |
| 69 | *Bidens pilosa* L.** | Asteraceae | Tsetsega (Sh) | Herb | Leaf | 2 | 0.05 | 0.17 | 94, 102 |
| 70 | *Blepharispermum villosum* O. Hoffm. * | Asteraceae | Boniya (O) | Shrub | Leaf, fruit | 1 | 0.03 | 0.33 | 118 |
| 71 | *Blyttia fruticulosa* (Decne.) D.V. Field** | Apocynaceae | Lamtta (K) | Shrub | Fruit | 2 | 0.05 | 0.17 | 135, 136 |
| 72 | *Borassus aethiopum* Mart.** | Arecaceae | Gosha (Sh) | Tree | Fruit, Leaf | 3 | 0.08 | 0.33 | 91, 94, 117 |
| 73 | *Boscia angustifolia* A. Rich. * | Capparidaceae |  | Shrub | Seed | 1 | 0.03 | 0.17 | 91 |
| 74 | *Boscia coriacea* Pax** | Capparidaceae | Karkarota/Qarqarota/Hurhurota (K), Homura/Aytneba (Af) | Tree | Fruit | 2 | 0.05 | 0.17 | 125, 135 |
| 75 | *Boscia mossambicensis* Klotzsch* | Capparidaceae |  | Shrub | Fruit | 1 | 0.03 | 0.17 | 117 |
| 76 | *Boscia salicifolia* Oliv. ** | Capparidaceae |  | Shrub | Leaf, Fruit | 2 | 0.05 | 0.33 | 92, 134 |
| 77 | *Boswellia microphylla* Chiov. * | Burseraceae | Leeddi (O/Af) | Shrub/  Tree | Fruit | 1 | 0.03 | 0.17 | 99 |
| 78 | *Boswellia neglecta* S.Moore* | Burseraceae |  | Tree | Fruit | 1 | 0.03 | 0.17 | 102 |
| 79 | *Boswellia papyrifera* (Del.) Hochst. ** | Burseraceae | Lubaten (Af); MukeItana (O) | Tree | Other parts | 1 | 0.03 | 0.17 | 92 |
| **No** | **Scientific name** | **Family name** | **Local name** | **Habit** | **Parts used** | **FC** | **RFC** | **RUV** | **References** |
| 80 | *Brassica carinata* A.Braun* | Brassicaceae | Baga (G), Raafuudaggalaa (O) | Shrub/  Herb | Leaf, Fruit | 3 | 0.08 | 0.33 | 42, 98, 123 |
| 81 | *Brassica napus* L.* | Brassicaceae | Teyla (K) | Herb | Leaf | 1 | 0.03 | 0.17 | 136 |
| 82 | *Brassica nigra* (L.) Koch* | Brassicaceae | Senafich (A) | Herb | Leaf | 1 | 0.03 | 0.17 | 97 |
| 83 | *Brassica rapa* L.* | Brassicaceae | Adri (T), Tenjilo (B) | Herb | Leaf, Seed, Fruit, Other parts | 2 | 0.05 | 0.67 | 98, 104 |
| 84 | *Bridelia micrantha* (Hochst.) Baill. ** | Phyllanthaceae | Aericho (M), Rigaaarba (O), yejega (G) | Shrub/  Tree | Fruit | 5 | 0.13 | 0.17 | 94, 100, 114, 118, 126 |
| 85 | *Bridelia scleroneura* Müll. Arg.** | Phyllanthaceae | Dayita-arba (K), BuneGalday (B), Ajega (G) | Shrub/  Tree | Fruit | 3 | 0.08 | 0.17 | 94, 98, 135 |
| 86 | *Brucea ferruginea* L’Herit. * | Simaroubaceae | Hadhawwaa (O) | Tree/  Shrub | Fruit | 1 | 0.03 | 0.17 | 118 |
| 87 | *Buckollia volubilis* (Schltr.) Venter & R.L. Verh. * | Apocynaceae | Lomba (K) | Climber | Underground part | 2 | 0.05 | 0.17 | 134, 135 |
| 88 | *Cadaba farinosa* Forssk. ** | Capparidaceae | Luqatasigmama (K), Fura (-Yito)/Numhele (Af), Keliknationha (O) | Shrub | Fruit, Leaf | 4 | 0.11 | 0.33 | 92, 102, 134, 136 |
| 89 | *Cajanus cajan* (L.) Millsp. * | Fabaceae | Ceate/Yemakkuatara (S) | Shrub | Fruit, Leaf | 2 | 0.05 | 0.33 | 129, 130 |
| 90 | *Canarina abyssinica* Engl.* | Campanulaceae | Xuxoo (O) | Herb | Fruit | 1 | 0.03 | 0.17 | 126 |
| 91 | *Canthium lactescens* Hiern* | Rubiaceae | Bolocket (B), korboo (O) | Shrub | Fruit | 2 | 0.05 | 0.17 | 98, 127 |
| 92 | *Canthium oligocarpum* Hiern *subsp. Oligocarpum** | Rubiaceae | Kincho (S) | Shrub | Other parts | 1 | 0.03 | 0.17 | 131 |
| 93 | *Canthium pseudosetiflorum* Bridson (Syn: Bullockia pseudosetiflora (Bridson) Razafim., Lantz &B.Bremer)** | Rubiaceae | Mayeta (K), Meddale (M) | Shrub | Fruit | 4 | 0.11 | 0.17 | 91, 114, 134, 135 |
| 94 | *Capparis cartilaginea* Decne. * | Capparidaceae | Delensisa (O) | Shrub | Fruit, Other parts | 1 | 0.03 | 0.33 | 92 |
| 95 | *Capparis tomentosa* Lam.** | Capparidaceae | Harenigema/Gora (O), Gumero (A), Kufo (Ko), Chiqua (G) | Shrub | Fruit, Other parts | 6 | 0.16 | 0.33 | 92, 93, 95, 128, 132, 138 |
| 96 | *Capsella bursa-pastoris* (L.) Medic. * | Brassicaceae | Yebeglat (A), Hamlieif (T) | Herb | Leaf, Seed, Other parts | 2 | 0.05 | 0.5 | 97, 104 |
| 97 | *Capsicum annuum* L. | Solanaceae |  | Shrub | Fruit | 1 | 0.03 | 0.17 | 102 |
| 98 | *Caralluma edulis* (Edgew.) Benth. & Hook.f* | Apocynaceae | Gumudo (A) | Herb | Flower, Leaf Other parts | 1 | 0.03 | 0.5 | 116 |
| 99 | *Cardamine trichocarpa* A. Rich. ** | Brassicaceae |  | Herb | Leaf | 1 | 0.03 | 0.17 | 93 |
| **No** | **Scientific name** | **Family name** | **Local name** | **Habit** | **Parts used** | **FC** | **RFC** | **RUV** | **References** |
| 100 | *Carissa spinarum L. (Syn: Carissa edulis (Forssk.) Vahl)*** | Apocynaceae | Agamsa/Aganssaa(O), Agamta/Akamitta (K), Agam (A), Ambelto (M), Almi (Ar), Hagalcho (S), Agama (B), Titita (Af), Siwa (G), Agamsa (Ko), Egam (T), Soha (G) | Shrub | Fruit, Leaf | 34 | 0.89 | 0.33 | 42, 91, 93, 94, 95, 96, 97, 98, 100, 102, 114, 115, 116, 117, 118, 119, 120, 122, 123, 124, 125, 126, 127, 128, 129, 130, 131, 132, 133, 134, 135, 136, 137, 138 |
| 101 | *Casimiroa edulis* La Llave* | Rutaceae | Kazamora (A) | Tree | Fruit, Flower | 2 | 0.05 | 0.33 | 116, 117 |
| 102 | *Catha edulis* (Vahl) Forssk. ex Endl* | Celastraceae | Jimaa (O) | Tree | Leaf | 1 | 0.03 | 0.17 | 42 |
| 103 | *Caylusea abyssinica* (Fresen.) Fisch. &Mey. ** | Resedaceae | Ilaancoo (O) | Herb | Leaf | 1 | 0.03 | 0.17 | 120 |
| 104 | *Ceiba pentandra* (L.) Gaertn* | Malvaceae | FerenjiTuti (Af) | Tree | Fruit | 1 | 0.03 | 0.17 | 92 |
| 105 | *Celosia anthelminthica* Asch. ** | Amaranthaceae |  | Herb | Fruit | 1 | 0.03 | 0.17 | 134 |
| 106 | *Celosia argentea* L.** | Amaranthaceae | Torchata/Torcha (K) | Herb | Leaf | 3 | 0.08 | 0.17 | 116, 135, 136 |
| 107 | *Celosia trigyna* L.** | Amaranthaceae | Torchata/Torqeta (K), Ambershuwa (G) | Herb | Leaf | 3 | 0.08 | 0.17 | 123, 135, 136 |
| 108 | *Celtis africana* Burm.f ** | Cannabaceae | Motoqommaa (O/Af), Qawo (G), Qewt (A) | Tree | Fruit | 7 | 0.18 | 0.17 | 94, 99, 102, 117, 119, 127, 132 |
| 109 | *Celtis toka* (Forssk.) Hepper & J.R.I.Wood** | Cannabaceae | Matoqoma (O), Gudibi’ato (Af); | Tree | Fruit | 2 | 0.05 | 0.17 | 92, 137 |
| 110 | *Chasmanthera dependens* Hochst. ** | Menispermaceae | Sorta-Arba (K) | Climber | Fruit | 2 | 0.05 | 0.17 | 134, 135 |
| 111 | *Chenopodium album* L.* | Amaranthaceae | Raafo (S) | Herb | Leaf, Other parts | 1 | 0.03 | 0.33 | 131 |
| 112 | *Chionanthus mildbraedii* (Gilg & Schellenb.) Stearn* | Oleaceae | KarraWayyuu (O) | Shrub | Fruit | 1 | 0.03 | 0.17 | 126 |
| 113 | *Chlorophytum laxum* R.Br.* | Liliaceae | Munna (Sh) | Herb | Underground part | 1 | 0.03 | 0.17 | 121 |
| 114 | *Chlorophytum tetraphyllum* (L.f.) Baker* | Asparagaceae | Sindelit (A) | Herb | Underground part | 1 | 0.03 | 0.17 | 97 |
| 115 | *Cissus populnea* Guill. &Perr. ** | Vitaceae | Ewa (G) | Shrub | Leaf | 1 | 0.03 | 0.17 | 123 |
| 116 | *Cissus quadrangularis* L.* | Vitaceae | AliI’e (Af); Chophi (O) | Climber | Fruit, Leaf | 1 | 0.03 | 0.33 | 92 |
| 117 | *Cissus rotundifolia* (Forssk.) Vahl* | Vitaceae | Buri (O) | Climber | Underground part | 1 | 0.03 | 0.17 | 92 |
| 118 | *Citrullus lanatus* (Thunb.) Matsum. &Nakai** | Cucurbitaceae | Blass (K) | Herb | Fruit | 1 | 0.03 | 0.17 | 135 |
| **No** | **Scientific name** | **Family name** | **Local name** | **Habit** | **Parts used** | **FC** | **RFC** | **RUV** | **References** |
| 119 | *Citrus aurantifolia* (Christm.) Swingle* | Rutaceae |  | Shrub | Fruit | 1 | 0.03 | 0.17 | 102 |
| 120 | *Citrus sinensis* (L.) osbeck* | Rutaceae | Burtukaana (O) | Tree | Fruit | 1 | 0.03 | 0.17 | 42 |
| 121 | *Clausena anisata* (Willd.) Benth. ** | Rutaceae | Xirdhoo/Ulumayaa (O), Limich (A) | Shrub | Fruit, Leaf | 3 | 0.08 | 0.33 | 100, 119 |
| 122 | *Cleome gynandra* L.** | Cleomaceae | Ketota/Kornia (K), Mikkidhi/Shanachaa (O), Shemuda (Ko) | Herb | Leaf, Other parts | 4 | 0.11 | 0.33 | 93, 104, 127, 135 |
| 123 | *Cleome monophylla* L.** | Capparidaceae | Doran (So) | Tree | Leaf | 2 | 0.05 | 0.17 | 91, 124 |
| 124 | *Clerodendrum myricoides* (Hochst.) Vatke* | Lamiaceae | Shochegufe (Ko) | Shrub | Fruit | 1 | 0.03 | 0.17 | 93 |
| 125 | *Clutia lanceolata* Forssk. * | Euphorbiaceae | Doguha (Sh) | Shrub | Fruit | 1 | 0.03 | 0.17 | 94 |
| 126 | *Coccinia abyssinica* (Lam.) Cogn. ** | Cucurbitaceae | Aamola/Hamola/Poteta-Karata (K), Tikurduba (Ko), Anchote/Wushish (A) | Climber | Leaf, Fruit, Underground part | 4 | 0.11 | 0.5 | 93, 116, 135, 138 |
| 127 | *Coccinia grandis* (L.) Voigt. ** | Cucurbitaceae | Lacheta (K), Werkbemeda (A) | Climber | Leaf, Fruit | 3 | 0.08 | 0.33 | 116, 134, 135 |
| 128 | *Coffea arabica* L.* | Rubiaceae | Buna (O) | Shrub | Seed, Fruit | 1 | 0.03 | 0.33 | 100 |
| 129 | *Colocasia esculenta* (L.) Schott* | Araceae | kompha (G,Sh), Sheta (Sh), Goodarree (O) | Herb | Underground part | 5 | 0.13 | 0.17 | 94, 102, 118, 121, 123 |
| 130 | *Combretum aculeatum* Vent. ** | Combretaceae | Qignfirta/Kignfirda (K) | Shrub | Fruit, Seed | 2 | 0.05 | 0.33 | 135, 136 |
| 131 | *Combretum collinum* Fresen. * | Combretaceae | Hafa (G), Yekolaavalo (A) | Tree | Seed, Other parts | 2 | 0.05 | 0.33 | 115, 123 |
| 132 | *Combretum molle* R.Br. ex G. Don** | Combretaceae | Birecha (O), Shawoch (A) | Tree | Fruit | 2 | 0.05 | 0.17 | 119, 137 |
| 133 | *Commelina africana* L.* | Commelinaceae | Holagabis (O), Echaya (G) | Herb | Leaf, Other parts | 2 | 0.05 | 0.33 | 94, 126 |
| 134 | *Commelina benghalensis* L.** | Commelinaceae | Holagabis (O), Yeberekolet (A) | Herb | Leaf, Underground part | 3 | 0.08 | 0.33 | 115, 126, 134 |
| 135 | *Commelina diffusa* Burm. f.** | Commelinaceae | Sindelit (A) | Herb | Leaf | 1 | 0.03 | 0.17 | 116 |
| 136 | *Commelina foliacea* Chiov. ** | Commelinaceae |  | Herb | Leaf | 1 | 0.03 | 0.17 | 134 |
| 137 | *Commelina imberbis* Ehrenb. Ex Hassk. ** | Commelinaceae |  | Herb | Leaf | 1 | 0.03 | 0.17 | 134 |
| 138 | *Commelina latifolia* Hochst.ex A. Rich. * | Commelinaceae | Wofeankure (A) | Herb | Leaf, Underground part | 2 | 0.05 | 0.33 | 93, 132 |
| 139 | *Commicarpus grandiflorus* (A.Rich.) Standl. * | Nyctaginaceae | Engurbaba (A) | Herb | Leaf | 1 | 0.03 | 0.17 | 97 |
| 140 | *Commiphora africana* Engl.** | Burseraceae | Hameessa (O), Anqwa (T) | Shrub/  Tree | Fruit, Underground part, Other parts | 5 | 0.13 | 0.5 | 104, 124, 127, 128, 137 |
| 141 | *Commiphora baluensis* Engl.** | Burseraceae | Hagarmadow (So) | Tree | Fruit | 1 | 0.03 | 0.17 | 123 |
| **No** | **Scientific name** | **Family name** | **Local name** | **Habit** | **Parts used** | **FC** | **RFC** | **RUV** | **References** |
| 142 | *Commiphora confuse* Vollesen** | Burseraceae |  | Shrub/  Tree | Underground part | 1 | 0.03 | 0.17 | 134 |
| 143 | *Commiphor aerythraea* (Ehrenb.) Engl.* | Burseraceae | Hagarssu (O) | Tree | Other parts | 1 | 0.03 | 0.17 | 127 |
| 144 | *Commiphora habessinica* (Berg) Engl.** | Burseraceae | Hedayito (Af); Hamesa (O) | Tree/Shrub | Fruit, Underground part, other parts | 2 | 0.05 | 0.5 | 92, 135 |
| 145 | *Commiphora kataf* (Forssk.) Engl.** | Burseraceae | Kahatta-ata (K) | Tree | Leaf | 2 | 0.05 | 0.17 | 135, 136 |
| 146 | *Commiphora rostrata* Engl.** | Burseraceae | Dabiisa (O) | Herb | Underground part | 1 | 0.03 | 0.17 | 127 |
| 147 | *Commiphora schimperi* (Berg.) Engl.** | Burseraceae | Challnaka/Hameessadaalachaa (O), Anka (A) | Tree/  Shrub | Underground part | 4 | 0.11 | 0.17 | 97, 117, 127, 137 |
| 148 | *Commiphora terebinthina* Vollesen** | Burseraceae | kahatta-timma (K), Cim'aa (O) | Tree/  Shrub | Underground part, other parts | 3 | 0.08 | 0.33 | 127, 135, 136 |
| 149 | *Convolvulus arvensis* L.* | Convolvulaceae | Kuent (T) | Herb | Other parts | 1 | 0.03 | 0.17 | 104 |
| 150 | *Corallocarpus schimperi* Hook.f.** | Cucurbitaceae |  | Climber | Leaf | 1 | 0.03 | 0.17 | 134 |
| 151 | *Corbichonia decumbens* (Forssk.) Exell** | Molluginaceae | Mocholo (K) | Herb | Leaf | 1 | 0.03 | 0.17 | 135 |
| 152 | *Corchorus olitorius* L.** | Malvaceae | Mulugaya (K), Kudra (A), Lalqa (G), Laliaq (Sh), Lalqa (G) | Herb | Leaf, Fruit | 5 | 0.13 | 0.33 | 94, 95, 123, 133, 135 |
| 153 | *Corchorus tridens* L.** | Malvaceae | Oloqloqota/Hachota (k) | Herb | Leaf | 2 | 0.05 | 0.17 | 134, 135 |
| 154 | *Corchorus trilocularis* L.** | Malvaceae | Oloqiloqota (K), Ged mide (A) | Herb | Leaf | 3 | 0.08 | 0.17 | 116, 134, 136 |
| 155 | *Cordeuxia edulis* Hemsl. ** | Fabaceae | Agam (T) | Tree | Fruit | 1 | 0.03 | 0.17 | 104 |
| 156 | *Cordia africana* Lam.** | Boraginaceae | Wdecha/Woddessa (O), Ottayta (K), Wanza (A/T), galmi (M), Waaddiicho (S), Mearera (B), Banja (G, Sh), Bola (Ko) | Tree | Fruit | 29 | 0.76 | 0.17 | 3, 42, 93, 94, 96, 97, 98, 100, 102, 104, 114, 115, 117, 118, 119, 123, 124, 126, 127, 128, 129, 130, 131, 132, 133, 135, 136, 137, 138 |
| 157 | *Cordia ellenbeckii* Gurke* | Boraginaceae | Dela’a (B), Madheedha (O) | Shrub/  Tree | Fruit | 2 | 0.05 | 0.17 | 98, 127 |
| **No** | **Scientific name** | **Family name** | **Local name** | **Habit** | **Parts used** | **FC** | **RFC** | **RUV** | **References** |
| 158 | *Cordia monoica* Roxb.** | Boraginaceae | Mendhero (O), Toloqota (K), Mine gure/Subula/Laem-mederto (Af), Chew wanza (A), Madeda (Ko) | Tree/  Shrub | Fruit | 10 | 0.26 | 0.17 | 92, 93, 102, 117, 129, 132, 134, 135, 136, 137 |
| 159 | *Cordia sinensis* Lam.** | Boraginaceae | Leedii (O), Madertta (K), Shengolochi (M), Mederto/Madera/Ledo (Af) | Tree/  Shrub | Fruit | 8 | 0.21 | 0.17 | 114, 117, 125, 129, 134, 135, 136, 137 |
| 160 | *Costus spectabilis* (Fenzl) K. Schum. * | Costaceae | Yampedema (G) | Shrub | Underground part | 1 | 0.03 | 0.17 | 123 |
| 161 | *Crateva adansonii* DC. ** | Capparidaceae |  | Shrub | Fruit, Underground part | 2 | 0.05 | 0.33 | 91, 117 |
| 162 | *Crossandra mucronata* Lindau* | Acanthaceae | Hiwak (O/Af) | Shrub/  Herb | Fruit | 1 | 0.03 | 0.17 | 99 |
| 163 | *Crotalaria incana* L.** | Fabaceae | Entartashilako (K) | Herb | Leaf | 1 | 0.03 | 0.17 | 135 |
| 164 | *Crotalaria polysperma* Kotschy** | Fabaceae |  | Herb | Leaf | 1 | 0.03 | 0.17 | 134 |
| 165 | *Croton macrostachyus* Del. | Euphorbiaceae | Shekeshek (Sh) | Tree | Leaf | 1 | 0.03 | 0.17 | 94 |
| 166 | *Cucumis dipsaceus* Ehrenb. ex Spach** | Cucurbitaceae | Dhahanata/Hashupata (K), yamoramisa (A) | Climber | Leaf | 4 | 0.11 | 0.17 | 116, 134, 135, 136 |
| 167 | *Cucumis ficifolius* A. Rich. * | Cucurbitaceae | hiddihooloo (O), Yebed (G)- Ye GurnaMatebiya (A) | Herb | Leaf, Underground part | 2 | 0.05 | 0.33 | 95, 126 |
| 168 | *Cucumis kirkbridei* Ghebretinsae & Thulin* | Cucurbitaceae |  | Climber | Leaf | 1 | 0.03 | 0.17 | 134 |
| 169 | *Cucurbita pepo* L.* | Cucurbitaceae | duba (T), maximara (Sh), dabaaqula (O) | Climber | Fruit, Leaf | 3 | 0.08 | 0.33 | 42, 94, 104 |
| 170 | *Cyanotis barbata* D.Don* | Commelinaceae |  | Herb | Other parts | 1 | 0.03 | 0.17 | 126 |
| 171 | *Cyathula uncinulata* (Schrad.) Schinz* | Amaranthaceae | maxannee (O) | Herb | Leaf | 1 | 0.03 | 0.17 | 126 |
| 172 | *Cynodon dactylon* (L.) Pers.* | Poaceae | serdo (A) | Herb | Other parts | 2 | 0.05 | 0.17 | 96, 138 |
| 173 | *Cyperus bulbosus* Vahl** | Cyperaceae | Hinkicha/Heqeyata (K), chachate (A), engicha/gichigla (A) | Herb | Other parts | 5 | 0.13 | 0.17 | 97, 119, 132, 135, 136 |
| 174 | *Cyperus esculentus* L.** | Cyperaceae | kurutatae (T) | Herb | Other parts | 1 | 0.03 | 0.17 | 104 |
| 175 | *Cyperus rigidifolius* Steud. * | Cyperaceae | angicha (A) | Herb | Other parts | 1 | 0.03 | 0.17 | 96 |
| 176 | *Cyperus rotundus* L.** | Cyperaceae | bambiya (Sh) | Herb | Underground part | 1 | 0.03 | 0.17 | 121 |
| 177 | *Cyperus usitatus* Burch. ** | Cyperaceae | Allaado (S) | Herb | Other parts | 1 | 0.03 | 0.17 | 131 |
| 178 | *Cyphia glandulifera* Hochst. ex A. Rich. * | Campanulaceae | Kurtee (O) | Herb | Leaf, Underground part | 1 | 0.03 | 0.33 | 118 |
| **No** | **Scientific name** | **Family name** | **Local name** | **Habit** | **Parts used** | **FC** | **RFC** | **RUV** | **References** |
| 179 | *Cyphostemma adenocaule* (Steud. ex A. Rich.) Des. ex-Wild & Drum. ** | Vitaceae | hiddaboffa | Herb | Leaf, Underground part | 1 | 0.03 | 0.33 | 126 |
| 180 | *Cyphostemma rivae* (Gilg) Desc. * | Vitaceae | coophiidabassitti (O) | Climber | Fruit | 1 | 0.03 | 0.17 | 127 |
| 181 | *Cyphostemma serpens* (Hochst. ex A.Rich.) Desc. * | Vitaceae | Coophii (O) | Climber | Fruit | 1 | 0.03 | 0.17 | 126 |
| 182 | *Datura stramonium* L.** | Solanaceae | astenager (A), Baanje (S), kobo (Ko), qobochuruko (O) | Herb/  Shrub | Flower | 7 | 0.18 | 0.17 | 93, 96, 102, 115, 119, 131, 132 |
| 183 | *Delonix regia* (Bojer ex Hook.) Raf. ** | Fabaceae |  | Tree | Seed | 1 | 0.03 | 0.17 | 134 |
| 184 | *Dichrostachys cinerea* (L.) Wight & Arn. * | Fabaceae | andera (A) | Tree/  Shrub | Fruit | 2 | 0.05 | 0.17 | 95, 133 |
| 185 | *Digera muricata* (L.) Mart.** | Amaranthaceae | Torchata/Torcha/Torqeta (K) | Herb | Leaf | 2 | 0.05 | 0.17 | 135, 136 |
| 186 | *Dioscorea abyssinica* Hochst.exKunth. ** | Dioscoreaceae | Bohe (S), yechaka buna (A) | Climber | Underground part | 2 | 0.05 | 0.17 | 119, 131 |
| 187 | *Dioscorea alata* L.* | Dioscoreaceae |  | Herb | Underground part | 1 | 0.03 | 0.17 | 122 |
| 188 | *Dioscorea bulbifera* L.** | Dioscoreaceae | Barodaa (O), yechiwa (G), Shawatmatakeya (Sh) | Climber | Fruit, Underground part | 4 | 0.11 | 0.33 | 118, 121, 123, 128 |
| 189 | *Dioscorea cayenensis* Lam.* | Dioscoreaceae | egera (G) | Climber | Underground part | 2 | 0.05 | 0.17 | 94, 122 |
| 190 | *Dioscorea praehensilis* Benth. ** | Dioscoreaceae | Sinsa (A), eca/anga/amejko (G), anga (Sh), buri (O) | Climber | Underground part | 7 | 0.18 | 0.17 | 94, 95, 100, 121, 122, 123, 133 |
| 191 | *Dioscorea schimperiana* Kunth** | Dioscoreaceae | baroodaa/barodii (O) | Climber | Underground part | 2 | 0.05 | 0.17 | 100, 127 |
| 192 | *Diospyros abyssinica* (Hiern) F. White** | Ebenaceae | Serkin (A) | Tree | Fruit | 4 | 0.11 | 0.17 | 95, 102, 133, 134 |
| 193 | *Diospyros mespiliformis* Hochst. ex A. DC. ** | Ebenaceae | Gurmacha.ayeh (A), goroki (M), ayeh (T), maranta (Sh) | Shrub/  Tree | Fruit | 8 | 0.21 | 0.17 | 3, 91, 94, 95, 104, 114, 117, 133 |
| 194 | *Discopodium penninervium* Hochst. * | Solanaceae | Bamlat (G)-Ameraro/Bolenta (A), goficho (O) | Tree | Leaf, Fruit | 2 | 0.05 | 0.33 | 95, 100 |
| 195 | *Dobera glabra* (Forssk.) Juss. ex Poir. ** | Salvadoraceae | Ade (O), Karssata (K), Ghersa/Gersayto/Gasera/Mudua (Af), Bekee (M) | Tree/  Shrub | Fruit, Seed | 8 | 0.21 | 0.33 | 92, 114, 125, 129, 134, 135, 136, 137 |
| 196 | *Dorstenia barnimiana* Schweinf. ** | Moraceae | Kuritata (K) | Herb | Underground part | 2 | 0.05 | 0.17 | 135, 136 |
| **No** | **Scientific name** | **Family name** | **Local name** | **Habit** | **Parts used** | **FC** | **RFC** | **RUV** | **References** |
| 197 | *Dovyalis abyssinica* (A. Rich.) Warb. ** | Salicaceae | Koshim (A), koshimii/akoku/dhugoo (O), Korqiicho (S), longo (B), koshm (Kei apple)/mongolhats(T) | Tree/  Shrub | Fruit | 19 | 0.50 | 0.17 | 3, 42, 93, 96, 97, 98, 102, 104, 115, 117, 118, 119, 120, 126, 130, 131, 132, 133, 138 |
| 198 | *Dovyalis caffra* (Hook.f. & Harv.) Hook.f.* | Salicaceae | koshim (A) | Shrub | Fruit | 1 | 0.03 | 0.17 | 119 |
| 199 | *Dovyalis verrucosa* (Hochst.) Warb. * | Flacourtiaceae | Akuukuu/Fatafullasu (O), teumtegna (T) | Shrub | Fruit | 3 | 0.08 | 0.17 | 120, 126, 130 |
| 200 | *Echium plantagineum* L.* | Boraginaceae | Shimbra-gomen (A) | Herb | Leaf | 1 | 0.03 | 0.17 | 97 |
| 201 | *Ehretia cymosa* Thonn. ** | Boraginaceae | Porporissa/Borborrissa (K), Mine Gure (Af), Ulaagaa (O), Game/Olaga (A), Gidiincho (S), Kolisha (B), Ulaga (Ko) | Tree/  Shrub | Fruit, Other parts | 10 | 0.26 | 0.33 | 7, 92, 93, 98, 100, 124, 131, 132, 135, 138 |
| 202 | *Ekebergia capensis* Sparrm. ** | Meliaceae | Somboo (O), Olooncho (S), Kudekuda/Lol (A) | Tree | Fruit | 4 | 0.11 | 0.17 | 95, 119, 126, 131 |
| 203 | *Embelia schimperi* Vatke** | Myrsinaceae | Hanquu/Enkoko (O), Enkoko (A), Qaanqo (S), Aserko (Ko) | Shrub/  Climber | Fruit, Seed, Leaf, Other parts | 11 | 0.29 | 0.67 | 93, 96, 100, 115, 118, 119, 126, 130, 131, 132, 138 |
| 204 | *Englerina woodfordioides* (Schweinf.) * | Loranthaceae | Telkokamo (Ko) | Shrub | Fruit | 1 | 0.03 | 0.17 | 93 |
| 205 | *Ensete ventricosum* (Welw.) Cheesman* | Musaceae | Warqee/Qocoo (O), Echecha (Sh), Koba/Enset (A) | Herb/  Tree | Fruit, Other parts | 6 | 0.16 | 0.33 | 42, 94, 96, 100, 119, 126 |
| 206 | *Eragrostis barrelieri* Dav. * | Poaceae | TikurTifafulet (A) | Herb | Seed | 1 | 0.03 | 0.17 | 97 |
| 207 | *Eragrostis cilianensis* (All.) Vign. ex Janche** | Poaceae | NechTifafulet (A) | Herb | Seed | 1 | 0.03 | 0.17 | 97 |
| 208 | *Eriosema cordifolium* Hochst. ex A. Rich. ** | Fabaceae | Silinga (O), Shilingo/Silinga (S) | Herb | Underground part | 2 | 0.05 | 0.17 | 118, 131 |
| 209 | *Eriosema nutans* Schinz* | Fabaceae | Kurte (B), Kurtee (O) | Herb | Underground part | 2 | 0.05 | 0.17 | 98, 127 |
| 210 | *Eriosema shirense* Bak.f*. | Fabaceae | Qamura (B) | Herb | Underground part | 1 | 0.03 | 0.17 | 98 |
| 211 | *Eriosema verdickii* De wild. * | Fabaceae | Qociqoo’male (S) | Herb | Underground part | 1 | 0.03 | 0.17 | 131 |
| 212 | *Erucastrum abysinicum* (A. Rich.) R.E. Fries** | Brassicaceae | Wefe (A) | Herb | Leaf | 1 | 0.03 | 0.17 | 97 |
| 213 | *Erucastrum pachypodum* (Chiov.) Jonsell* | Brassicaceae | Hakala koyra (K) | Herb | Leaf | 1 | 0.03 | 0.17 | 136 |
| **No** | **Scientific name** | **Family name** | **Local name** | **Habit** | **Parts used** | **FC** | **RFC** | **RUV** | **References** |
| 214 | *Erythrococca abyssinica* Pax** | Euphorbiaceae | caakoo (O) | Shrub | Fruit, Leaf | 2 | 0.05 | 0.33 | 100, 134 |
| 215 | *Erythroxylum fischeri* Engl.* | Erythroxylaceae | tiriga (G) | Herb | Leaf | 1 | 0.03 | 0.17 | 94 |
| 216 | *Euclea divinorum* Hiern. ** | Ebenaceae | Maqayita (K), ounsi (M), Mi'eessaa (O), measka (B) | Shrub/  Tree | Fruit | 7 | 0.18 | 0.17 | 91, 98, 114, 127, 128, 135, 136 |
| 217 | *Euclea racemose* Murr. ** | Ebenaceae | dedeho (A) | Shrub | Fruit | 1 | 0.03 | 0.17 | 115 |
| 218 | *Euclea racemosa subsp. Schimperi* (A.DC.) F.White* | Ebenaceae | Maqayta (K), Miessa (O), Dedeho (A) | Shrub/  Tree | Fruit | 6 | 0.16 | 0.17 | 35, 92, 93, 119, 132, 137 |
| 219 | *Eugenia uniflora* L.* | Musaceae | Badirbonga (Sh) | Shrub | Fruit | 1 | 0.03 | 0.17 | 94 |
| 220 | *Fagaropsis angolensis* (Engl.) Dale* | Rutaceae | Sissaa (O) | Tree | Other parts | 1 | 0.03 | 0.17 | 127 |
| 221 | *Ferula communis* L.** | Apiaceae | Dog/inslal (A), Nugusa (S), Shlian (T) | Herb | Leaf, Other parts | 6 | 0.16 | 0.33 | 96, 97, 104, 115,131, 138 |
| 222 | *Festuca abyssinica* Hochst. ex A. Rich. * | Poaceae | Garbudadde (O) | Herb | Fruit | 1 | 0.03 | 0.17 | 126 |
| 223 | *Ficus abutilifolia* (Miq.) Miq. ** | Moraceae | Hobanhobata (K) | Tree | Fruit | 1 | 0.03 | 0.17 | 135 |
| 224 | *Ficus capreaefolia* Del.** | Moraceae |  | Tree | Fruit | 1 | 0.03 | 0.17 | 116 |
| 225 | *Ficus carica* L.* | Moraceae | beles (A) | Tree | Fruit | 1 | 0.03 | 0.17 | 116 |
| 226 | *Ficus glumosa* Delile** | Moraceae | Halota/Tinaita (K), Kuntsee (M), Qilxuu (O), Sholla.woda (A) | Tree | Fruit | 5 | 0.13 | 0.17 | 114, 119, 128, 134, 135 |
| 227 | *Ficus ingens* (Miq.) Miq. ** | Moraceae | Tinaita (K), Laze (M), Qilinxo (O) | Tree | Fruit | 6 | 0.16 | 0.17 | 93, 114, 116, 126, 135, 136 |
| 228 | *Ficus ovata* Vahl** | Moraceae | Warka fere (O), Warka (A) | Tree | Fruit | 3 | 0.08 | 0.17 | 116, 124, 130 |
| 229 | *Ficus palmata* Forssk. ** | Moraceae | Beles/Qollabeles (A), Belese (S), Guma (B) | Shrub/  Tree | Fruit | 5 | 0.13 | 0.17 | 97, 98, 116, 119, 131 |
| 230 | *Ficus platyphylla* Del.** | Moraceae | Leiya/Rerumma (K), Titee (M) | Tree | Fruit | 3 | 0.08 | 0.17 | 114, 135, 136 |
| 231 | *Ficus sur* Forssk. ** | Moraceae | Heleta (K), Shola (A), semo (M), sema (Ar), harbuu/harruu/arbu (O), Odakko (S), Sublaito (Af), elisho (B), echa (G), bobo (Ko), essa (Sh), shamfa (T) | Tree | Fruit | 28 | 0.74 | 0.17 | 42, 91, 93, 94, 95, 96, 97, 98, 100, 102, 114, 115, 116, 117, 118, 119, 120, 123, 126, 127, 128, 129, 131, 132, 133, 135, 136, 138 |
| 232 | *Ficus sycomorus* L.** | Moraceae | Oda/harbu/luugoo (O), Hillteta (K), SUBULA (Af), Bamba/wodda (A), bobi (M), shafa (Ar), sagla (T), oda (Ko), fuqa (G) | Tree | Fruit, Other parts | 25 | 0.66 | 0.33 | 115, 42, 116, 117, 118, 119, 120, 95, 122, 94,93, 126, 127, 99, 102, 130, 132, 114, 133, 92, 134, 135, 136, 137, 138 |
| **No** | **Scientific name** | **Family name** | **Local name** | **Habit** | **Parts used** | **FC** | **RFC** | **RUV** | **References** |
| 233 | *Ficus sycomorus subsp. gnaphalocarpa* (Miq.) C.C.Berg* | Moraceae | dawitchi (M), tomiri (Ar), | Tree | Fruit | 2 | 0.05 | 0.17 | 104, 114 |
| 234 | *Ficus thonningii* Blume** | Moraceae | Dambi (O), Tinaita/Chaqerta (K), chibeha(A) | Tree | Fruit, Other parts | 5 | 0.13 | 0.33 | 95, 126 |
| 235 | *Ficus vallis-choudae* Del.** | Moraceae | Bambula (A), obori (M) | Tree | Fruit | 2 | 0.05 | 0.17 | 114, 133 |
| 236 | *Ficus vasta* Forssk. ** | Moraceae | Qilxu/qilxaa (O), Leiya (K), MARA’ITO/Sublaito (Af), Warka (A), shabi (M), wompa (Ar), Qilxo (S), heca/qilxu/bowa (G), gahee (Ko) | Tree | Fruit, Leaf, Other parts | 24 | 0.63 | 0.5 | 91, 92, 93, 94, 96, 97, 100, 102, 104, 114, 115, 117, 119, 123, 126, 127, 129, 131, 132, 133, 134, 135, 137, 138 |
| 237 | *Flacourtia indica* (Burm.f.) Merr. ** | Flacourtiaceae | gurchinchi (M), hudhaa/hagallaa (O), daero (T), dunadunise (B), akuk (A) | Tree/  Shrub | Fruit | 10 | 0.26 | 0.17 | 91, 92, 93, 98, 114, 118, 119, 126, 127, 128, |
| 238 | *Flueggea leucopyrus* Willd. ** | Phyllanthaceae | Chalanchalota/Hepata (K) | Shrub | Fruit | 2 | 0.05 | 0.17 | 135, 136 |
| 239 | *Flueggea virosa* (Roxb. ex Willd.) Royle** | Phyllanthaceae | Kecacule (O), Qarchechelo (B), Shasha/loge (A) | Shrub | Fruit | 6 | 0.16 | 0.17 | 98, 102, 117, 132, 133, 137 |
| 240 | *Foeniculum vulgare* Miller* | Apiaceae | Qushuwa (SH) | Herb | Leaf | 1 | 0.03 | 0.17 | 94 |
| 241 | *Fuerstia africana* T.C. E.Fr.* | Lamiaceae | Sinaqayish (B) | Herb | Leaf | 1 | 0.03 | 0.17 | 98 |
| 242 | Galinieracoffeoides Del.* | Rubiaceae | Kudhumi (O) | Tree | Fruit | 1 | 0.03 | 0.17 | 118 |
| 243 | *Galiniera saxifraga* (Hochst.) Bridson* | Rubiaceae | Daanshiicho (S) | Tree | Fruit | 1 | 0.03 | 0.17 | 131 |
| 244 | *Garcinia livingstonei* T. Anderson** | Clusiaceae | chedi (Ar), tulla (O) | Tree | Fruit | 3 | 0.08 | 0.17 | 114, 130, 134 |
| 245 | *Gardenia ternifolia* Schumach. & Thonn. ** | Rubiaceae | Brmaiyta (K), Gambillo (A), Kota/gaaba (G) | Tree/  Shrub | Fruit | 7 | 0.18 | 0.17 | 94, 95, 117, 123, 133, 135, 138 |
| 246 | *Gardenia volkensii* K. Schun. * | Rubiaceae |  | Tree | Fruit | 1 | 0.03 | 0.17 | 132 |
| 247 | *Girardinia bullosa* (Steudel) Wedd. * | Urticaceae | dobii (O) | Herb | Leaf | 1 | 0.03 | 0.17 | 126 |
| 248 | *Gladiolus candidus* (Rendle) Goldblatt* | Iridaceae | Silinqqaa (O), Engula (Sh) | Herb | Underground part, Leaf | 2 | 0.05 | 0.33 | 122, 126 |
| 249 | *Gloriosa superba* L.* | Liliaceae | Yemariamtwa (A) | Herb/  Shrub | Fruit | 1 | 0.03 | 0.17 | 133 |
| 250 | *Gnidia somalensis* (Franch.) Gilg. * | Thymelaeaceae | anbura (B) | Herb | Underground part | 1 | 0.03 | 0.17 | 98 |
| 251 | *Gossypium hirsutum* L.* | Malvaceae | tit (A) | Shrub | Seed | 1 | 0.03 | 0.17 | 97 |
| 252 | *Grewia arborea* (Forssk.) Lam.** | Tiliaceae |  | Shrub | Fruit | 1 | 0.03 | 0.17 | 134 |
| **No** | **Scientific name** | **Family name** | **Local name** | **Habit** | **Parts used** | **FC** | **RFC** | **RUV** | **References** |
| 253 | *Grewia balensis* M.G. Gilbert & Sebsebe** | Tiliaceae | Dawaita (K) | Shrub | Fruit | 1 | 0.03 | 0.17 | 135 |
| 254 | *Grewia bicolor* Juss. ** | Tiliaceae | Harorecha/haroressa (O), Dhahayta/Daiyta/Dahita/Dawaita/Qochata (K), ADIBI’ATO/hebele (Af), benine (Ko), somoya (Sh), somaya (A) | Shrub/  Tree | Fruit, Seed | 13 | 0.34 | 0.33 | 92, 93, 94, 102, 116, 117, 124, 125, 127, 132, 135, 136, 137 |
| 255 | *Grewia erythraea* Schweinf. ** | Tiliaceae | Chaqalesa/Chaqlessa (K), hidayto (Af), benine (Ko) | Shrub | Fruit | 5 | 0.13 | 0.17 | 93, 125, 134, 135, 136 |
| 256 | *Grewia ferruginea* Hochst. ex A. Rich. ** | Tiliaceae | Chaqalesa/Qocheta/Daieta-Damale (K), Adibi’ato/Fo (Af); HARORESA/laanqessaa/dhoqonuu (O), lenquata (A), benine (Ko), galqoriya (Sh) | Shrub/  Tree | Flower, Fruit | 16 | 0.42 | 0.33 | 42, 92, 93, 94, 97, 102, 115, 116, 117, 119, 126, 127, 132, 135, 136, 138 |
| 257 | *Grewia flavescens* Juss. ** | Tiliaceae | Amurjii (O), Daiyta-arba (K) | Shrub | Fruit | 2 | 0.05 | 0.17 | 135, 137 |
| 258 | *Grewia lilacina* K. Schum. ** | Tiliaceae | Kocheta (K) | Shrub | Fruit | 2 | 0.05 | 0.17 | 134, 135 |
| 259 | *Grewia mollis* Juss. ** | Tiliaceae | Daiyta (K), Harroresssa (O), gediya/qoriya (G), Betere Mussie (A), rewey (T) | Tree/  Shrub | Fruit, 0ther parts | 9 | 0.24 | 0.33 | 93, 94, 95, 102, 120, 123, 130, 134, 135 |
| 260 | *Grewia schweinfurthii* Burret** | Tiliaceae | Mudhegurre (O), Adibi’ato/Ditayito (Af), damage (M), badiriya (G) | Shrub | Fruit | 5 | 0.13 | 0.17 | 92, 94, 114, 129, 137 |
| 261 | *Grewia tenax* (Forssk.)Fiorii** | Tiliaceae | Eka fila/Deka Tuntana/Chemerda (O), Chaqlessa/Daiyta/Horma-Daiyta/Daieta-Konso (K), Hedayito/Huda/Mine Gure/Hidayto (Af), hoba (A) | Tree/  Shrub | Fruit | 8 | 0.21 | 0.17 | 91, 92, 116, 124, 129, 134, 135, 137 |
| 262 | *Grewia trichocarpa* Hochst. Ex A. Rich. ** | Tiliaceae | Dawaita/Daiyta/Ahawteta-Daiyta (K), alenkuato (O) | Shrub | Fruit | 2 | 0.05 | 0.17 | 130, 135 |
| 263 | *Grewia velutina* (Forssk.) Lam.** | Tiliaceae | Dhahayta/Dayita/Ahawteta-Daiyta/Dayita/Horma-Daeyta/Dawaita (K), Adibi’Ato(Af); Harorreessa (O) | Tree/  Shrub | Fruit | 7 | 0.18 | 0.17 | 91, 92, 117, 127, 134, 135, 136 |
| 264 | *Grewia villosa* Willd. ** | Tiliaceae | Ogomdi (O), Ogomteta/Qopissa/Poqsa/Qoffissa/Offissa/Hoppissa/Ogomteta (K), GARIWA/habeleyta (Af), bonkako (M), agubite (A), andereko (Ko) | Tree/  Shrub | Seed, Fruit, Leaf | 13 | 0.34 | 0.5 | 91, 92, 93, 99, 102, 114, 117, 125, 132, 134, 135, 136, 137 |
| 265 | *Guizotia scabra* (Vis.) Chiov. ** | Asteraceae | hadaa/tuufoo (O) | Herb | Leaf | 1 | 0.03 | 0.17 | 126 |
| 266 | *Hagenia abyssinica* (Bruce) J.F. Gmel. * | Rosaceae | heto/heexoo (O), koso (A) | Tree | Seed, Fruit, Leaf | 3 | 0.08 | 0.5 | 42, 96, 130 |
| 267 | *Haplocarpha schimperi* (Sch. Bip.) Beauv. * | Asteraceae | getin (A), Nechilo (A) | Herb | Leaf, Fruit, Other parts | 2 | 0.05 | 0.5 | 97, 132 |
| **No** | **Scientific name** | **Family name** | **Local name** | **Habit** | **Parts used** | **FC** | **RFC** | **RUV** | **References** |
| 268 | *Haplocoelum foliolosum* (Hiern) Bullock* | Sapindaceae | cannaa (O) | Tree | Fruit | 2 | 0.05 | 0.17 | 118, 127 |
| 269 | *Hibiscus cannabinus* L.** | Malvaceae | YeberhaWayika (A), tisha (G) | Herb | Fruit | 4 | 0.11 | 0.17 | 94, 95, 102, 133 |
| 270 | *Hibiscus micranthus* L. f.* | Malvaceae | AKILEHENA (Af) | Herb | Fruit | 1 | 0.03 | 0.17 | 92 |
| 271 | *Hibiscus ovalifolius* (Forssk.) Vahl* | Malvaceae | Dheekaa (O) | Shrub | Fruit | 1 | 0.03 | 0.17 | 126 |
| 272 | *Hibiscus sabdariffa* L.* | Malvaceae | ejega (G) | Shrub | Fruit, Seed | 1 | 0.03 | 0.33 | 123 |
| 273 | *Hoslundia opposita* Vahl** | Lamiaceae | imwarwarsha/Timwarwarsha/Segenata (K), lisho (M) | Shrub | Fruit | 3 |  | 0.17 | 114, 134, 135 |
| 274 | *Huernia engleri* Terracc. * | Apocynaceae | Yesew-qolqollo (A) | Herb | Other parts | 1 | 0.03 | 0.17 | 97 |
| 275 | *Huernia macrocarpa* (A.Rich.) Sprenger *subsp. Macrocarpa** | Apocynaceae | Yeahya-qolqollo (A) | Herb | Other parts | 1 | 0.03 | 0.17 | 97 |
| 276 | *Hydnora abyssinica* A.Br. Ex Decne. * | Aristolochiaceae |  | Herb | Fruit | 1 | 0.03 | 0.17 | 134 |
| 277 | *Hyphaene thebaica* (L.) Mart.** | Arecaceae | Kunchula (K), UNGA/GARA’ITO (Af); METI (O) | Tree | Fruit | 3 | 0.08 | 0.17 | 92, 117, 135 |
| 278 | *Hypoestes forskaolii* (Vahil.) R.Br.** | Anacardiaceae | Gichigela (Ko) | Herb | Leaf | 1 | 0.03 | 0.17 | 93 |
| 279 | *Ilex mitis* (L.) Radlk. * | Aquifoliaceae | mi’eesa (O) | Tree | Other parts | 1 | 0.03 | 0.17 | 126 |
| 280 | *Indigofera arrecta* Hochst. ex A. Rich. * | Fabaceae | Herchumen (Or) | Herb | Underground part | 1 | 0.03 | 0.17 | 92 |
| 281 | *Indigofera coerulea* Roxb. * | Fabaceae | Adulala (Or) | Herb | Fruit | 1 | 0.03 | 0.17 | 92 |
| 282 | *Ipomoea batatas* (L.) Lam.* | Convolvulaceae | Bambeya (G) | Herb/  Climber | Underground part | 1 | 0.03 | 0.17 | 123 |
| 283 | *Ipomoea biflora* (L.) Pers.* | Convolvulaceae |  | Herb/  Shrub | Leaf | 1 | 0.03 | 0.17 | 134 |
| 284 | *Ipomoea coscinosperma* Hochst. ex Choisy** | Convolvulaceae | Songoderderta (K) | Herb | Leaf | 1 | 0.03 | 0.17 | 135 |
| 285 | *Ipomoea hildebrandtii* Vatke* | Convolvulaceae | Amborokke (O) | Shrub | Fruit | 1 | 0.03 | 0.17 | 118 |
| 286 | *Ipomoea marmorata* Britten & Rendle** | Convolvulaceae |  | Shrub | Underground part | 1 | 0.03 | 0.17 | 134 |
| 287 | *Ipomoea sinensis* (Desr.) Choisy** | Convolvulaceae | Horbaia/Hossohorbaia (K) | Herb | Leaf | 1 | 0.03 | 0.17 | 135 |
| 288 | *Justicia calyculata* Deflers** | Acanthaceae | Kurkuncha/Randolla (K) | Herb | Leaf | 2 | 0.05 | 0.17 | 134, 135 |
| 289 | *Justicia flava* (Forssk) Vahl** | Acanthaceae | Honnona (K) | Herb | Leaf | 2 | 0.05 | 0.17 | 135, 136 |
| 290 | *Justicia ladanoides* Lam.** | Acanthaceae | Qira/Qirqira (K), elangiya/kakim (G) | Herb | Leaf | 3 | 0.08 | 0.17 | 94, 123, 135 |
| 291 | *Justicia schimperiana* (Hochst. ex Nees) T. Anders. ** | Acanthaceae | dhuumuga (O), Ciikkicho (S), muga (G), dumuga (Sh), smitha (A) | Shrub | Flower | 5 | 0.13 | 0.17 | 94, 119, 123, 126, 131 |
| 292 | *Kedrostis foetidissima* Cogn.** | Cucurbitaceae |  | Climber | Leaf | 1 | 0.03 | 0.17 | 134 |
| 293 | *Kedrostis leloja* (Forssk. ex J.F.Gmel.) C. Jeffrey** | Cucurbitaceae |  | Climber | Leaf | 1 | 0.03 | 0.17 | 134 |
| 294 | *Kedrostis pseudogijef* (Gilg) C. Jeffrey** | Cucurbitaceae | Essatta (K) | Climber | Leaf, Fruit | 3 | 0.08 | 0.33 | 134, 135, 136 |
| 295 | *Kniphofia isoetifolia* Steud. ex Hochst* | Asphodelaceae | Galade (S) | Herb | Flower | 1 | 0.03 | 0.17 | 131 |
| **No** | **Scientific name** | **Family name** | **Local name** | **Habit** | **Parts used** | **FC** | **RFC** | **RUV** | **References** |
| 296 | *Lablab purpureus* (L.) Sweet* | Fabaceae | Kugnarata (K) | Herb | Leaf | 1 | 0.03 | 0.17 | 136 |
| 297 | *Lagenaria siceraria* (Molina) Standl. ** | Cucurbitaceae | Heriya (K), Surupha (S) | Climber | Seed, Fruit | 2 | 0.05 | 0.33 | 131, 136 |
| 298 | *Landolphia buchananii* (Hallier f.) Stapf** | Apocynaceae | Hidagebo (O) | Shrub/  Tree | Fruit | 1 | 0.03 | 0.17 | 125 |
| 299 | *Lannea humilis* (Oliv.) Engl.** | Anacardiaceae |  | Tree | Underground part | 1 | 0.03 | 0.17 | 117 |
| 300 | *Lannea rivae* (Chiov.) Sacleux* | Anacardiaceae | Orittatta (K), handarakku (O) | Tree/  Shrub | Leaf, Fruit, Underground part, other parts | 3 | 0.08 | 0.67 | 127, 128, 135 |
| 301 | *Lannea schimperi* (Hochst. ex A. Rich.) ** | Anacardiaceae | Oraiyta (K), Anderaku (B), handarakubadda (O) | Tree/ Shrub | Fruit | 4 | 0.11 | 0.17 | 91, 98, 127, 135 |
| 302 | *Lannea triphylla* Engl.** | Anacardiaceae | Orritta/Orittata (K) | Tree | Fruit, Underground part | 2 | 0.05 | 0.33 | 134, 135 |
| 303 | *Lantana camara* L.** | Verbenaceae | Midhaandubara/hamarreessa (O), BADUWA HARA/Dat'hara (Af), yewofqolo (A) | Shrub/  Herb | Fruit | 7 | 0.18 | 0.17 | 92, 93, 102, 116, 128, 129, 137 |
| 304 | *Lantana trifolia* L.* | Verbenaceae | Punita purkaya/Bunitaburayo/Punita burayou (K), Midhandurbaa (O/Af), hantite (O), kefakosa (Ko), Argagifo (A) | Shrub/  Herb | Fruit | 7 | 0.18 | 0.17 | 93, 99, 132, 134, 135, 136, 138 |
| 305 | *Lantana viburnoides* (Forssk.) Vahl* | Verbenaceae | Qarqando (B), dubaroroo (O) | Shrub | Fruit | 2 | 0.05 | 0.17 | 98, 127 |
| 306 | *Launaea intybacea* (Jacq.) Beauverd** | Asteraceae | Hankolayta (K) | Herb | Leaf | 3 | 0.08 | 0.17 | 134, 135, 136 |
| 307 | *Launaea taraxacifolia* (Willd.) Amin ex C. Jeffrey** | Asteraceae | Hankolayta (K) | Herb | Leaf | 1 | 0.03 | 0.17 | 136 |
| 308 | *Lepidium sativum* L.* | Brassicaceae | shinafae (T) | Herb | Seed, Leaf | 1 | 0.03 | 0.33 | 104 |
| 309 | *Lepidotrichillia volkensii* (Gurke) Leroy** | Meliaceae | Xabbicho (S) | Tree | Fruit | 2 | 0.05 | 0.17 | 126, 131 |
| 310 | *Lepisanthes senegalensis* (Poir) Leenh.** | Sapindaceae | bekuda (Sh) | Shrub | Fruit | 1 | 0.03 | 0.17 | 94 |
| 311 | *Leptadenia hastata* Vatke** | Apocynaceae | Khayla/Xeyla (K) | Shrub | Leaf, Fruit, Other parts | 2 | 0.05 | 0.5 | 135, 136 |
| 312 | *Leptadenia lancifolia* (Schum. & Thonn.) Decne. * | Apocynaceae |  | Climber | Leaf | 1 | 0.03 | 0.17 | 134 |
| 313 | *Leucas glabrata* (Vahl) Sm.** | Lamiaceae |  | Shrub | Fruit | 1 | 0.03 | 0.17 | 91 |
| 314 | *Lippia adoensis* Hochst. ex Walp*. | Verbenaceae | kussayee (O) | Shrub | Leaf, Other parts | 2 | 0.05 | 0.33 | 42, 126 |
| 315 | *Lonchocarpus laxiflorus* Guill. &Perr* | Fabaceae | beewa (G) | Tree | Flower | 1 | 0.03 | 0.17 | 123 |
| 316 | *Luffa cylindrica* (L.) M. J. Roem.** | Cucurbitaceae | bedaha (G) | Herb | Leaf | 1 | 0.03 | 0.17 | 123 |
| **No** | **Scientific name** | **Family name** | **Local name** | **Habit** | **Parts used** | **FC** | **RFC** | **RUV** | **References** |
| 317 | *Lycopersicon esculentum* Mill. * | Solanaceae | TsebhiAbun (T), komidira (Sh), Abafinjale (A) | Herb | Fruit | 3 | 0.08 | 0.17 | 94, 104, 138 |
| 318 | *Maerua angolensis* DC. ** | Capparidaceae | DUNIBIAYITO/SEKILELI’A (Af) | Shrub | Fruit, Leaf | 2 | 0.05 | 0.33 | 92, 134 |
| 319 | *Maerua subcordata* (Gilg) De Wolf** | Capparidaceae | Pa-atasheka (K) | Shrub | Fruit | 2 | 0.05 | 0.17 | 134, 135 |
| 320 | *Maesa lanceolata* Forssk. * | Myrsinaceae | Gowacho, gecha (S), abayi (O) | Tree | Fruit | 3 | 0.08 | 0.17 | 93, 130, 131 |
| 321 | *Mangifera indica* L.* | Anacardiaceae |  | Tree | Fruit | 1 | 0.03 | 0.17 | 102 |
| 322 | *Manihot esculenta* Crantz. * | Euphorbiaceae | Kazawari (Sh) | Tree | Underground part | 1 | 0.03 | 0.17 | 95 |
| 323 | *Manilkara butugi* Chiov. ** | Sapotaceae | BUTUYE (Af); BUTUJI (O), koshimi (Ar) | Tree | Fruit | 2 | 0.05 | 0.17 | 114, 92 |
| 324 | *Maytenu sarbutifolia* (A.Rich.) Wilczek. * | Celastraceae |  | Shrub | Fruit | 1 | 0.03 | 0.17 | 102 |
| 325 | *Maytenus senegalensis* (Lam.) Exell** | Celastraceae | Firkuta/Yeregna-chat/Koshikosh (A), xasha/tisha (G), | Shrub/  Herb | Leaf, Fruit, Other parts | 6 | 0.16 | 0.5 | 94, 97, 102, 117, 123, 133 |
| 326 | *Meriandra bengalensis* (Koenig ex Roxb.) Benth. * | Lamiaceae | Hadha toke (O) | Shrub | Leaf | 1 | 0.03 | 0.17 | 137 |
| 327 | *Meyna tetraphylla* (Schweinf. ex Hiern) Robyns* | Rubiaceae | onakii (M) | Shrub | Fruit | 2 | 0.05 | 0.17 | 91, 114 |
| 328 | *Mimusops kummel* Bruce ex A. DC. ** | Sapotaceae | Tulukanta/Tuleta (K). Ishe/shiy/qoladi (A), gosho (M), ollatii/qolati (O), yelow eta (Af), kumel (T), shemiya (Sh) | Tree | Fruit | 15 | 0.39 | 0.17 | 94, 96, 104, 114, 115, 116, 117, 118, 119, 125, 127, 130, 133, 135, 138 |
| 329 | *Mimusops laurifolia* (Forssk.) Friis** | Sapotaceae | Yealelito (Af) | Tree | Fruit | 1 | 0.03 | 0.17 | 129 |
| 330 | *Momordica foetida* Schumach. ** | Cucurbitaceae | Ki’re (S), sarambaw/Suruphaa/nyataallatii (O), marqah (T), badha (Sh), YequraMechate/Ye jib medihanit/yamoramisa (A), shetebigno (G) | Climber | Fruit, Leaf, Underground part | 9 | 0.24 | 0.5 | 94, 95, 100, 104, 118, 119, 126, 128, 131 |
| 331 | *Momordica trifoliolata* Hook.f*. | Cucurbitaceae | Koricha Aja (O) | Climber | Fruit | 1 | 0.03 | 0.17 | 92 |
| 332 | *Moringa oleifera* Lam.* | Moringaceae | shiferaw (A) | Tree | Leaf | 1 | 0.03 | 0.17 | 116 |
| 333 | *Moringa stenopetala* (Baker f.) Cufod.** | Moringaceae | shiifaraa/shalqeeda (O), haleko (Ko), sheferwu (Sh), shiferaw (A) | Tree/  Shrub | Leaf | 8 | 0.21 | 0.17 | 92, 93, 94, 102, 116, 124, 127, 134 |
| 334 | *Morus alba* L.** | Moraceae | Boowiligo’ra (S), injor (Sh), gora (O), nechenjori (A) | Shrub/  Tree | Fruit | 4 | 0.11 | 0.17 | 94, 100, 116, 131 |
| 335 | *Morus mesozygia* Stapf** | Moraceae | Injori (A) | Climber | Fruit | 2 | 0.05 | 0.17 | 117, 133 |
| 336 | *Morus nigra* L.* | Moraceae | Tikurenjori (A) | Tree | Fruit | 1 | 0.03 | 0.17 | 116 |
| 337 | *Musa x paradisiaca* L.* | Musaceae | warke (O) | Herb | Fruit | 1 | 0.03 | 0.17 | 126 |
| 338 | *Mussaenda arcuate* Poir.** | Rubiaceae | murtsetse (M), sertsegela (Ar) | Shrub | Fruit | 1 | 0.03 | 0.17 | 114 |
| 339 | *Myrica salicifolia* Hochst. ex-A. Rich. * | Myricaceae | Biiqa (O), buddule (B) | Tree | Other parts | 2 | 0.05 | 0.17 | 98, 137 |
| 340 | *Myrsine africana* L.** | Myrsinaceae | qacaama (O), kechemo/eurche (A), chuchurina (B), eurch (Ko), qechemo (T) | Shrub | Fruit, Seed | 6 | 0.16 | 0.33 | 93, 98, 120, 126, 130, 132 |
| **No** | **Scientific name** | **Family name** | **Local name** | **Habit** | **Parts used** | **FC** | **RFC** | **RUV** | **References** |
| 341 | *Nasturtium officinale* W.T. Aiton* | Brassicaceae | guguble (A) | Herb | Seed, Leaf, Flower, Fruit, Other parts | 1 | 0.03 | 0.83 | 116 |
| 342 | *Nauclea latifolia* Smith* | Rubiaceae |  | Shrub/  Tree | Fruit | 1 | 0.03 | 0.17 | 117 |
| 343 | *Nicotiana tabacum* L.* | Solanaceae | tamboo (O) | Herb | Leaf | 1 | 0.03 | 0.17 | 42 |
| 344 | *Nymphaea nouchali* Burm.f.** | Nymphaeaceae |  | Herb | Seed | 1 | 0.03 | 0.17 | 134 |
| 345 | *Ochna leucophloeos* Hochst. ex A. Rich** | Ochnaceae | Amedoli (A) | Herb | Leaf | 1 | 0.03 | 0.17 | 95 |
| 346 | *Ocimum americanum* L.** | Lamiaceae | Dikito (Ko) | Herb | Leaf, Flower, Seed | 1 | 0.03 | 0.5 | 93 |
| 347 | *Ocimum forskolei* Benth. ** | Lamiaceae | Kurutatita/Kurittatita (K) | Herb | Leaf, Flower | 1 | 0.03 | 0.33 | 135 |
| 348 | *Ocimum lamiifolium* Hochst. ex-Benth. * | Lamiaceae | anchabii/Damaakasee (O) | Shrub | Leaf | 2 | 0.05 | 0.17 | 42, 126 |
| 349 | *Ocimum spicatum* Deflers* | Lamiaceae | MISE (Af); KORCHA MICHI (O) | Shrub | Seed | 1 | 0.03 | 0.17 | 92 |
| 350 | *Ocimum urticifolium* Roth* | Lamiaceae | anchabii/hancabii (O), ziqaqibe/checho (A) | Shrub | Leaf, Flower | 4 | 0.11 | 0.33 | 96, 100, 119, 126 |
| 351 | *Olea capensis* L.* | Oleaceae |  | Tree | Fruit | 1 | 0.03 | 0.17 | 117 |
| 352 | *Olea europaea L. subsp. cuspidata* (Wall. ex G.Don) Cif.** | Oleaceae | ejeerssa (O/S). Wagerto (Af), yeger (B), woira (A) | Tree | Fruit, Leaf, Underground part | 7 | 0.18 | 0.5 | 96, 98, 126, 127, 128, 129, 131 |
| 353 | *Olinia rochetiana* A. Juss. * | Oliniaceae | Adde (O/Af) | Shrub | Fruit | 2 | 0.05 | 0.17 | 99, 102 |
| 354 | *Oncoba spinosa* Forssk. ** | Flacourtiaceae | sewembulko (M), antsitsa (G), ula (Sh) | Shrub | Fruit, Other parts | 5 | 0.13 | 0.33 | 94, 102, 114, 117, 123 |
| 355 | *Opuntia ficus-indica* (L.) Miller** | Cactaceae | Hadamii, bushuki(O) Kombolta/ Papaldotta/Qulqualita (K), beles/qulqual/bahr qulqual/beles (A), qolqol (Af), Gambora (B), gamborraa (O), beles (T), akre (Ko) | Shrub/  Tree | Fruit, Seed | 23 | 0.61 | 0.33 | 3, 42, 91, 93, 96, 97, 98, 104, 115, 117, 118, 119, 120, 124, 127, 128, 129, 130, 132, 135, 136, 137, 138 |
| 356 | *Osyris quadripartita* Salzm. ex Decn. ** | Santalaceae | Wato (K), keret (A), tunka (B), wato (Ko) | Shrub | Fruit | 5 | 0.13 | 0.17 | 93, 97, 98, 132, 135 |
| 357 | *Oxalis corniculata* L.** | Oxalidaceae | Melgissa (K), Soddaare'ee (), yelam chew (A) | Herb | Flower, Seed, Leaf | 3 | 0.08 | 0.5 | 119, 127, 135 |
| 358 | *Oxalis latifolia* Kunth* | Oxalidaceae | Michamicho (A) | Herb | Leaf, Flower, Seed, Underground part, other parts | 1 | 0.03 | 0.83 | 138 |
| **No** | **Scientific name** | **Family name** | **Local name** | **Habit** | **Parts used** | **FC** | **RFC** | **RUV** | **References** |
| 359 | *Oxalis obliquifolia* A. Rich. * | Oxalidaceae | Lamcho (A) | Herb | Leaf | 1 | 0.03 | 0.17 | 115 |
| 360 | *Oxalis stricta* L.* | Oxalidaceae | yebere chew (A) | Herb | Leaf, Flower, Seed, Underground part | 1 | 0.03 | 0.67 | 116 |
| 361 | *Oxygonum sinuatum* (Hochst. & Steud. ex Meisn.) Dammer** | Polygonaceae | Moqorqorsa/Mororoqissa, kabeta (K), Yebere-chew (A) | Herb | Leaf, Seed | 3 | 0.08 | 0.33 | 97, 135, 136 |
| 362 | *Oxytenanthera abysinica* (A. Rich.) Munro** | Poaceae | Hojiya/enta/soha(G) | Herb | Other parts | 2 | 0.05 | 0.17 | 94, 123 |
| 363 | *Ozoroa pulcherrima* (Schweinf.) R. & A. Fernand* | Anacardiaceae | ejimbeya (G) | Herb | Other parts | 1 | 0.03 | 0.17 | 123 |
| 364 | *Pachycymbium laticoronum* (M.G. Gilbert) M.G. Gilbert* | Apocynaceae | Parapaqa/Pappaqa(K) | Herb | Leaf, Flower, Seed, Other parts | 2 | 0.05 | 0.67 | 135, 136 |
| 365 | *Pancratium tenuifolium* A. Rich. * | Amaryllidaceae | Liqaaqaa (O) | Shrub | Leaf | 1 | 0.03 | 0.17 | 126 |
| 366 | *Pappea capensis* Eckl. &Zeyh. ** | Sapindaceae | Biiqqaa (O), biqa (B) | Tree/  Shrub | Fruit | 4 | 0.11 | 0.17 | 98, 118, 127, 128 |
| 367 | *Parkinsonia aculeata* L.* | Fabaceae | Kunto-barbarie (K) | Tree/  Shrub | Seed, Fruit | 2 | 0.05 | 0.33 | 92, 135 |
| 368 | *Pavetta abyssinica* Fresen.** | Rubiaceae | Shamelcho (S) | Shrub | Fruit | 1 | 0.03 | 0.17 | 131 |
| 369 | *Pavetta crassipes* K.Schum.** | Rubiaceae | munqa (Sh) | Shrub | Fruit | 1 | 0.03 | 0.17 | 94 |
| 370 | *Pavetta oliveriana* Hiern* | Rubiaceae | Tolkokamo (Ko) | Shrub | Fruit | 1 | 0.03 | 0.17 | 93 |
| 371 | *Pentarrhinum insipidum* E. Mey. ** | Apocynaceae | Kokordota/Kordota (K), gumud (A) | Climber | Leaf, Fruit, Other parts | 3 |  | 0.5 | 116, 134, 135 |
| 372 | *Peponium vogelii* (Hook.f.) Engl.** | Cucurbitaceae | Bukeesexana (O), equmetsa (G) | Herb | Fruit | 2 | 0.05 | 0.17 | 123, 126 |
| 373 | *Persea americana* Mill. * | Lauraceae | Avokaadoo (O) | Tree | Fruit | 1 | 0.03 | 0.17 | 42 |
| 374 | *Persicaria nepalensis* (Meisn.) Miyabe* | Polygonaceae | lanbut (A) | Herb | Other parts | 1 | 0.03 | 0.17 | 96 |
| 375 | *Phoenix reclinata* Jacq. ** | Arecaceae | zembaba (Ar), meexii/metti (O), zembaba/senel/chifar (A), domaa (O/Af), Molowa/Metiya (G), ele (Ko), wola (Sh) | Tree | Fruit, Leaf, Other parts | 15 | 0.39 | 0.5 | 42, 93, 94, 95, 99, 100, 114, 115, 117, 118, 119, 123, 126, 132, 138 |
| 376 | *Phragmites karka* (Retz.) Steud. * | Poaceae | Meka (A) | Herb | Underground part | 1 | 0.03 | 0.17 | 97 |
| 377 | *Physalis lagascae* Roem. & Schult. * | Solanaceae | awet (O), Kurpakurpo (Ko) | Herb | Fruit | 2 | 0.05 | 0.17 | 93, 130 |
| 378 | *Physalis peruviana* L.** | Solanaceae | Hawteta/shewa/luketa/karma/Kumanata (k), awte/awat/awot/nechawet/Komeydero (A), Maree’ra (S), hawuxii/yeferenjetimatime/buqulii/subbaruufoo (O), tunaye (B), ejisiya (G), bosiya (Sh) | Herb | Fruit, Flower, Other parts | 17 | 0.45 | 0.5 | 42, 93, 94, 98, 100, 115, 116, 118, 119, 123, 127, 128, 131, 132, 135, 136, 138 |
| **No** | **Scientific name** | **Family name** | **Local name** | **Habit** | **Parts used** | **FC** | **RFC** | **RUV** | **References** |
| 379 | *Piliostigma thonningii* (Schum.) Milne-Redh. (Syno: *Bauhinia thonningii* Schumach.)** | Fabaceae | Kota karita (K), mecha/mijire (G), mac'a (Sh), yekolawanza (A), dawrake (M), lol (Ar) | Tree | Seed, Fruit, Other parts, Underground part | 7 | 0.18 | 0.67 | 91, 94, 95, 114, 117, 123, 135 |
| 380 | *Piper capense* L. f.* | Piperaceae | tunjo (O) | Herb | Fruit | 1 | 0.03 | 0.17 | 100 |
| 381 | *Pistacia lentiscus subsp. emarginata* (Engl.) Al-Saghir* | Anacardiaceae |  | Tree | Fruit | 1 | 0.03 | 0.17 | 117 |
| 382 | *Pittosporum viridiflorum* Sims* | Pittosporaceae | DengaySeber/galazabiya (A), roha (G), gaallo (O) | Shrub/  Tree | Fruit | 3 | 0.08 | 0.17 | 95, 118, 133 |
| 383 | *Plumbago zeylanica* L.* | Plumbaginaceae | Melekuya (G), Amera (A) | Herb | Leaf | 1 | 0.03 | 0.17 | 95 |
| 384 | *Podocarpus falcatus* (Thunb.) R. B. ex Mirb. ** | Podocarpaceae | zigiba (A) | Tree | Fruit | 1 | 0.03 | 0.17 | 119 |
| 385 | *Polypogon viridis* (Gouan) Breistr. * | Poaceae | Killaa (O/Af) | Shrub | Fruit | 1 | 0.03 | 0.17 | 99 |
| 386 | *Polysphaeria parvifolia* Hiern* | Rubiaceae | Miqqee (O) | Shrub | Fruit | 1 | 0.03 | 0.17 | 126 |
| 387 | *Portulaca oleracea* L.** | Portulacaceae | laha (K), bela/Rejila (G), Yenebosasega (A) | Herb/  Shrub | Leaf, Seed, Other parts | 5 | 0.13 | 0.5 | 95, 116, 122, 123, 135 |
| 388 | *Portulaca quadrifida* L.** | Portulacaceae | Maraeitta/Mecheritta (K), kiwa (Sh) | Herb | Leaf, Seed, Other parts | 4 | 0.11 | 0.5 | 94, 116, 134, 135 |
| 389 | *Pouzolzia parasitica* (Forssk.) Schweinf. * | Urticaceae | Qannatata (K) | Herb | Leaf | 1 | 0.03 | 0.17 | 136 |
| 390 | *Premna resinosa* (Hochest.) Schauer** | Lamiaceae | Dodoatteta (K), Urgecha (O), BOBA’O (Af) | Shrub | Fruit | 4 | 0.11 | 0.17 | 92, 134, 135, 137 |
| 391 | *Premna schimperi* Engl.* | Lamiaceae | Xaxessa (O), gellelo (Ko) | Shrub | Fruit, Leaf | 2 | 0.05 | 0.33 | 93, 128 |
| 392 | *Prosopis juliflora* (Sw.) DC. ** | Fabaceae | WEYANE (Af&O) | Shrub | Fruit, Seed | 2 | 0.05 | 0.33 | 92, 102 |
| 393 | *Prunus africana* (Hook.f.) ** | Rosaceae | Garbicho (S), komma (A), kookii (O) | Tree | Fruit | 3 | 0.08 | 0.17 | 42, 119, 131 |
| 394 | *Psophocarpus grandiflorus* R. Wilczek* | Fabaceae | qooqee/wokkallaa (O) | Herb | Fruit, Leaf | 1 | 0.03 | 0.33 | 127 |
| 395 | *Psydrax schimperiana* (A. Rich.) Bridson** | Rubiaceae | Kahelta (K), gali (M), gellelo (O) | Tree/Shrub | Fruit | 5 | 0.13 | 0.17 | 91, 93, 114, 135, 136 |
| 396 | *Psydrax schimperiana* (A. Rich.) Bridson subsp. Schimperiana* | Rubiaceae | gaalloo (O) | Tree | Fruit | 1 | 0.03 | 0.17 | 127 |
| 397 | *Pteridium aquilinum* (L.) Kuhn* | Dennstaedtiaceae | gixoo (O) | Herb | Leaf | 1 | 0.03 | 0.17 | 100 |
| 398 | *Pterolobium stellatum* (Forssk.) Brenan* | Fabaceae | kontir/Kentefa (A) | Shrub | Fruit, Other parts | 2 | 0.05 | 0.33 | 132, 138 |
| 399 | *Pupalia micrantha* Hauman** | Amaranthaceae |  | Herb | Leaf | 1 | 0.03 | 0.17 | 134 |
| 400 | *Pyrenacantha malvifolia* Engl.* | Icacinaceae | Burii (O) | Herb | Underground part | 1 | 0.03 | 0.17 | 118 |
| **No** | **Scientific name** | **Family name** | **Local name** | **Habit** | **Parts used** | **FC** | **RFC** | **RUV** | **References** |
| 401 | *Pyrostria phyllanthoidea* (BailL.) Bridson** | Rubiaceae | nandhalla (O) | Shrub | Fruit | 1 | 0.03 | 0.17 | 127 |
| 402 | *Raphanus raphanistrum* L.* | Brassicaceae | Bedhaka (B), yewefgomen (O) | Herb | Leaf, Fruit | 2 | 0.05 | 0.33 | 93, 98 |
| 403 | *Rhamnus prinoides* L’Herit. ** | Rhamnaceae | geshe/gesho (O), Xaddo (S) | Shrub/  Tree | Leaf, Fruit, Seed, Other parts | 4 | 0.11 | 0.67 | 93, 100, 126, 131 |
| 404 | *Rhamnus staddo* A.Rich.** | Rhamnaceae | Qadida (O) | Shrub | Leaf | 1 | 0.03 | 0.17 | 126 |
| 405 | *Rhoicissus tridentata* (L. f.) Wild & Drummond** | Vitaceae |  | Shrub | Fruit | 1 | 0.03 | 0.17 | 126 |
| 406 | *Rhus glutinosa* A. Rich. ** | Anacardiaceae | Zergete (Ko), Xaaxxessaa (O), Kamo/Imbis (A) | Shrub/  Tree | Fruit | 5 | 0.13 | 0.17 | 42, 93, 96, 115, 127 |
| 407 | *Rhus glutinosa subsp. Abyssinica* (Oliv.) M. Gilbert* | Anacardiaceae | Qamo (A), tetaelo (T) | Shrub | Fruit | 3 | 0.03 | 0.17 | 31, 102, 120 |
| 408 | *Rhus glutinosa subsp. glutinosa* Gilbert* | Anacardiaceae | Ashiqammo (A) | Shrub | Fruit | 1 | 0.03 | 0.17 | 119 |
| 409 | *Rhus glutinosa* Rich. *subsp. neoglutinosa* (M. Gilbert) M. Gilbert* | Anacardiaceae | embis (A) | Tree | Fruit | 1 | 0.03 | 0.17 | 97 |
| 410 | *Rhus longipes* Engl.** | Anacardiaceae | xaxeessa (O) | Shrub | Fruit, Other parts | 1 | 0.03 | 0.33 | 127 |
| 411 | *Rhus natalensis* Bernh. ex C.Krauss** | Anacardiaceae | Dabobecha/irqaaqammo/laboobessaa (O) Kabutayta (K), kubri (M), chakma (A), dewa/sofa (Af), tsisano (Ko), atami (T) | Shrub/  Tree | Fruit | 13 | 0.34 | 0.17 | 42, 93, 102, 114, 118, 120, 125, 127, 128, 132, 135, 136, 137 |
| 412 | *Rhus quartiniana* A. Rich. * | Anacardiaceae |  | Shrub | Seed | 1 | 0.03 | 0.17 | 91 |
| 413 | *Rhus retinorrhoea* Oliv. ** | Anacardiaceae | telem (A), kefijanga (Sh) | Shrub/  Tree | Fruit | 2 | 0.05 | 0.17 | 94, 132 |
| 414 | *Rhus ruspolii* Engl.** | Anacardiaceae | Pichioraya/Pichaoraya (K), shawshini (M), dodobay (B), qamo (Sh) | Shrub/  Tree | Fruit | 4 | 0.11 | 0.17 | 94, 98, 114, 135 |
| 415 | *Rhus tenuinervis* Engl.** | Anacardiaceae | kimosh/irqaaqammoo (O), qadhadhiya (B) | Shrub | Fruit | 3 | 0.08 | 0.17 | 98, 127, 130 |
| 416 | *Rhus vulgaris* Meikle** | Anacardiaceae | BichaOraya (K), dabobessaa/kimo/xaaxessaa (O), kimmo/qamo (A), qadhadhiya (B), bakitela (Sh) | Shrub/  Tree | Fruit, Leaf, Other parts | 12 | 0.32 | 0.5 | 92, 93, 94, 98, 115, 118, 126, 127, 132, 134, 135, 138 |
| 417 | *Rhynchosia alluaudi* Sack. ** | Fabaceae | Holla (K) | Shrub | Leaf | 1 | 0.03 | 0.17 | 135 |
| 418 | Rhynchosia minima (L.) DC. ** | Fabaceae |  | Herb | Leaf | 1 | 0.03 | 0.17 | 134 |
| 419 | Ricinus communis L.* | Euphorbiaceae | gulo (A), Qosha (G), tsema/guloo (O) | Shrub/  Tree | Seed, Fruit, Underground part | 4 | 0.11 | 0.5 | 93, 118, 123, 132 |
| 420 | Ritchieaalbersii Gilg** | Capparidaceae | dhakacabsi/gaqqo (O), Baxaraqicho (S), chomiye (A) | Shrub/  Tree | Fruit | 4 | 0.11 | 0.17 | 100, 119, 126, 131 |
| **No** | **Scientific name** | **Family name** | **Local name** | **Habit** | **Parts used** | **FC** | **RFC** | **RUV** | **References** |
| 421 | *Romulea fischeri* Pax. * | Iridaceae | Yeregna-kollo (A) | Herb | Seed | 1 | 0.03 | 0.17 | 97 |
| 422 | *Rosa abyssinica* R.Br.ex Lindley.** | Rosaceae | Qega (A), qaqawwee, gora (O), Otila (S), atim (Af), kega (T), gora (Ko), kega (A) | Shrub/  Climber | Fruit | 18 | 0.47 | 0.17 | 42, 93, 96, 97, 102, 104, 115, 116, 118, 119, 120, 125, 126, 130, 131, 132, 134, 138 |
| 423 | *Rubus apetalus* Poir.** | Rosaceae | sak “b” (Ar), gora/altufa (O), Enjori/Enzorgie (A), WorichuGo’ra (S), sinada (G), yedegaenjory (Ko), mongolil (T) | Shrub/  Climber | Fruit | 14 | 0.37 | 0.17 | 42, 93, 96, 97, 100, 102, 114, 119, 120, 122, 123, 126, 131, 138 |
| 424 | *Rubus Fruticosus* L.* | Moraceae | enjori (A) | Shrub | Fruit | 1 | 0.03 | 0.17 | 116 |
| 425 | *Rubus niveus* Thunb. * | Rosaceae | goragafarsa (O) | Climber | Fruit | 1 | 0.03 | 0.17 | 100 |
| 426 | *Rubus steudneri* Schweinf. ** | Rosaceae | sak “a” (Ar), gorra/goraaarbaa, hayena (O), injori (A), shaqar (B), waga (G), gorra (Ko) | Shrub | Fruit | 10 | 0.26 | 0.17 | 42, 93, 114, 119, 123, 126, 127, 130, 132, |
| 427 | *Rubus volkensii* Engl.** | Rosaceae | AllichchuGo’ra (S), aquqottadhangagoo (O) | Shrub | Fruit, Leaf | 3 | 0.08 | 0.33 | 93, 130, 131 |
| 428 | *Ruellia patula* Jacq. * | Acanthaceae | boboyta (Af) | Shrub | Fruit | 1 | 0.03 | 0.17 | 125 |
| 429 | *Rumex abyssinicus* Jacq. ** | Polygonaceae | mooqmoqii/Dhaangagoo/enbacho (O), Embari-kolla (A), meqmeqo (A, T), Shishoone (S), hopho (B), ambata (Sh) | Herb | Leaf, Other parts, Underground part | 15 | 0.39 | 0.5 | 93, 94, 96, 97, 98, 100, 104, 115, 116, 119, 126, 128, 131, 132, 138 |
| 430 | *Rumex nepalensis* Spreng. * | Polygonaceae | tult (A), timijjii (O) | Herb | Leaf, Other parts | 2 | 0.05 | 0.33 | 97, 100 |
| 431 | *Rumex nervosus* Vahl** | Polygonaceae | Dhaangagoo (O), Embacho/anbuatie (A), hehot/hakot (T) | Shrub/  Herb | Leaf, Fruit, Underground part, Other parts | 16 | 0.42 | 0.67 | 42, 93, 96, 97, 104, 115, 116, 117, 118, 119, 120, 124, 126, 132, 137, 138 |
| 432 | *Ruta chalepensis* L.* | Rutaceae | tsenaadam (T) | Herb | Leaf, Flower, Seed | 1 | 0.03 | 0.5 | 104 |
| 433 | *Rytigynia neglecta* (Hiern) Robyns** | Rubiaceae | Metagure/miqee (O) | Shrub | Fruit | 2 | 0.05 | 0.17 | 118, 130 |
| 434 | *Saba comorensis* (Boj.) Pichon** | Apocynaceae | Ashama (A), kalikedo (M), Huya (G), fuya (Sh) | Climber | Fruit, Seed | 6 | 0.16 | 0.33 | 94, 95, 114, 117, 123, 133 |
| 435 | *Sageretia thea* (Osbeck) M.C. Johnston** | Rhamnaceae | Qenchilegam (), kichilagam (A) | Shrub | Fruit | 2 | 0.05 | 0.17 | 116, 120 |
| **No** | **Scientific name** | **Family name** | **Local name** | **Habit** | **Parts used** | **FC** | **RFC** | **RUV** | **References** |
| 436 | *Salacia congolensis* De Wild & Th. Dur. * | Celastraceae | tsera (Sh) | Shrub | Other parts | 1 | 0.03 | 0.17 | 94 |
| 437 | *Salvadora persica* L.** | Salvadoraceae | Riga Ilkani (O), Ateta (K), Hadayito/Dadaho/Adayaito (Af); Ade (Or), Adey (So) | Shrub | Fruit, Leaf | 7 | 0.18 | 0.33 | 91, 92, 124, 129, 134, 135, 137 |
| 438 | *Salvia nilotica* Jacq. * | Lamiaceae | Tolellat (A) | Herb | Seed | 2 | 0.05 | 0.17 | 97, 126 |
| 439 | *Salvia schimperi* Benth. * | Lamiaceae | Gime-kitel (A) | Herb | Flower | 1 | 0.03 | 0.17 | 97 |
| 440 | *Sansevieria ehrenbergii* Schweinf. ex-Baker* | Asparagaceae | Algeti/chiret (A) | Herb | Other parts | 1 | 0.03 | 0.17 | 119 |
| 441 | *Sapium ellipticum* (Krauss) Pax* | Euphorbiaceae | Andirgago (Sh) | Shrub | Fruit | 1 | 0.03 | 0.17 | 94 |
| 442 | *Sarcocephalus latifolius* (Smith) E. A. Bruce* | Rubiaceae | bakeya (G) | Tree | Fruit | 1 | 0.03 | 0.17 | 123 |
| 443 | *Sarcostemma viminale* (L.) R.Br.* | Apocynaceae | hangayya (O) | Shrub | Fruit | 1 | 0.03 | 0.17 | 127 |
| 444 | *Satureja abyssinica* (Benth.) Briq. * | Lamiaceae | shusha (B) | Herb | Leaf | 1 | 0.03 | 0.17 | 98 |
| 445 | *Satureja punctata* (Benth.) Briq. ** | Lamiaceae | xossinyii/xossinyiijaldessaa (O) | Shrub | Leaf, Flower | 1 | 0.03 | 0.33 | 126 |
| 446 | *Sauromatum venosum* (Ait.) Kunth* | Araceae | Bukki-bu’re (S) | Herb | Underground part | 1 | 0.03 | 0.17 | 131 |
| 447 | *Schinus molle* L.** | Anacardiaceae | Qundo Berbere (A), Qundibarbaree (O) | Tree | Fruit | 2 | 0.05 | 0.17 | 42, 119 |
| 448 | *Schlechterella abyssinica* (Chiov.) Venter & R.L. Verh. ** | Apocynaceae |  | Climber | Underground part | 1 | 0.03 | 0.17 | 134 |
| 449 | *Sclerocarya birrea* (A. Rich.) Hochst. * | Anacardiaceae | Paata (Ye berehalomi) (K), tunkelo (M), hudhaa (O), boita (B) | Tree | Fruit | 9 | 0.24 | 0.17 | 91, 98, 114, 116, 118, 128, 134, 135, 136, |
| 450 | *Securidaca longepedunculata* Fresen. * | Polygalaceae |  | Tree | Leaf | 1 | 0.03 | 0.17 | 91 |
| 451 | *Sedum mooneyi* M.Gilbert* | Crassulaceae | Yelem-tut (A) | Herb | Underground part | 1 | 0.03 | 0.17 | 97 |
| 452 | *Senna obtusifolia* (L.) H.S. Irwin & Barneby** | Fabaceae | Bamdisa (G) | Shrub | Seed | 2 | 0.05 | 0.17 | 94, 135 |
| 453 | *Senna occidentalis* (L.) Link** | Fabaceae | FERAHIYITI (Af); SHESHEKISA (O), gelese (Ko) | Herb | Seed, Fruit | 2 | 0.05 | 0.33 | 92, 93 |
| 454 | *Senna petersiana* (Bolle) Lock* | Fabaceae | Zoyra (Ko), raamso (O), Yeleba mar (A) | Shrub | Fruit | 3 | 0.08 | 0.17 | 93, 100, 138 |
| 455 | *Senna singueana* (Del.) Lock** | Fabaceae | Hanqarara (K), karahaleko (M) | Shrub | Fruit, Leaf, Seed | 4 | 0.11 | 0.5 | 91, 114, 135, 136, |
| 456 | *Sida collina* Schltdl. ** | Malvaceae | Garuwyto (O/Af) | Herb | Fruit | 1 | 0.03 | 0.17 | 99 |
| 457 | *Sida ovata* Forssk. * | Malvaceae | Qirqixxe (S) | Herb | Leaf | 1 | 0.03 | 0.17 | 131 |
| 458 | *Sideroxylon oxyacanthum* Baill.** | Sapotaceae | Furakassa/bittee (O) | Tree | Fruit | 2 | 0.05 | 0.17 | 126, 130 |
| 459 | *Sisymbrium officinale* (L) Scop. * | Brassicaceae | Senafich (A) | Herb | Leaf | 1 | 0.03 | 0.17 | 116 |
| 460 | *Snowdenia polystachya* Fresen.) Pilg. * | Poaceae | muja (A) | Herb | Seed | 1 | 0.03 | 0.17 | 97 |
| 461 | *Solanum americanum* Mill.** | Solanaceae | Chankanchankota/Kahakaha (K), key awut (A) | Herb | Leaf, Fruit | 3 | 0.08 | 0.33 | 97, 134, 135 |
| **No** | **Scientific name** | **Family name** | **Local name** | **Habit** | **Parts used** | **FC** | **RFC** | **RUV** | **References** |
| 462 | *Solanum glabratum* Dunal* | Solanaceae | yeweshaberberie (A) | Shrub | Flower | 1 | 0.03 | 0.17 | 132 |
| 463 | *Solanum indicum* L.* | Solanaceae | samaree (O) | Herb | Fruit | 1 | 0.03 | 0.17 | 42 |
| 464 | *Solanum nigrum* L.** | Solanaceae | Qaqaha/Qahaqaha (K), SARA KORPO/hadhaa'aa/adua/anshu (O), tikurawut/awtign/keyeawat (A), Xu’naayye (S), tunaye (B), func'a (Sh), alamo (T) | Herb/  Shrub | Fruit, Leaf, Other parts | 18 | 0.47 | 0.5 | 92, 93, 94, 96, 97, 98, 100, 102, 115, 116, 119, 120, 122, 127, 131, 132, 136, 138 |
| 465 | *Solanum villosum* Mill.* | Solanaceae | tunaye (B) | Herb | Leaf | 1 | 0.03 | 0.17 | 98 |
| 466 | *Sorghum arundinaceum* (Desv.) Stapf* | Poaceae | Yetota (wild sorghum) (K) | Herb | Seed | 1 | 0.03 | 0.17 | 136 |
| 467 | *Sporobolus africanus* (Poir) Robyns and Tournay** | Poaceae | Muriye (A), murii (O) | Herb | Seed | 2 | 0.05 | 0.17 | 126, 133 |
| 468 | *Sporobolus pyramidalis* P. Beauv.** | Poaceae | Kurbata (K), mure (A) | Herb | Seed | 2 | 0.05 | 0.17 | 115, 135 |
| 469 | *Sterculia africana* (Lour.) Fiori** | Malvaceae | Qararu (O), Qawreta (K), kautsee (M), qereri (B) | Tree | Seed, Fruit, Other parts | 7 | 0.18 | 0.5 | 92, 98, 114, 134, 135, 136, 137 |
| 470 | *Stereospermum kunthianum* Cham. * | Bignoniaceae | andegila (G) | Shrub | Other parts | 1 | 0.03 | 0.17 | 123 |
| 471 | *Strychnos innocua* Delile** | Loganiaceae | jalijecho (M), Oola (Sh) | Tree | Fruit | 3 | 0.08 | 0.17 | 94, 114, 117 |
| 472 | *Strychnos spinosa* Lam.* | Loganiaceae | merenza (G) | Tree | Fruit | 2 | 0.05 | 0.17 | 94, 117 |
| 473 | *Syzygium guineense* (Willd.) DC.** | Myrtaceae | Tukuma (K), Dokima (A), ochi (M), shiringi (Ar), Duuwancho (S), bedessa, awajo/gootuu (O), liham (T) | Tree | Fruit | 15 | 0.39 | 0.17 | 95, 96, 104, 114, 115, 116, 118, 122, 124, 128, 130, 131, 133, 135, 138 |
| 474 | *Syzygium guineense supsp. afromontanum* F. White* | Myrtaceae | baddessaa (O), diwa (G), shawa (G) | Tree | Fruit | 4 | 0.11 | 0.17 | 93, 100, 126, 127 |
| 475 | *Syzygium guineense supsp. Guineense* (Willd.) DC.* | Myrtaceae | gossu/awajo/gumare (O), shelelay (B), oche (Ko), daguwa (G), bedessa (A) | Tree | Fruit | 11 | 0.29 | 0.17 | 93, 94, 98, 100, 102, 117, 119, 122, 123, 126, 127 |
| 476 | *Syzygium guineense subsp. macrocarpa* (Engl.) F. White* | Myrtaceae | Awajo/Goosu (O), Oche (Ko), Diwa (Sh) | Tree | Fruit | 4 | 0.11 | 0.17 | 94, 93, 100, 127 |
| 477 | *Tacazzea conferta* N.E. Br.* | Apocynaceae |  | Climber | Underground part | 1 | 0.03 | 0.17 | 126 |
| 478 | *Tamarindus indica* L.** | Fabaceae | Rooqa (O), Rokohta (K), SEGENTU/hura (Af), Kumer/roka/humer (A), rokee (M), humer (T), dogha (G,Sh), yebereha lomi (Ko) | Tree | Fruit, Flower, Seed | 21 | 0.55 | 0.5 | 91, 92, 93, 94, 95, 99, 102, 104, 114, 116, 117, 119, 123, 124, 125, 128, 133, 134, 135, 136, 137 |
| **No** | **Scientific name** | **Family name** | **Local name** | **Habit** | **Parts used** | **FC** | **RFC** | **RUV** | **References** |
| 479 | *Tarenna graveolens* (S. Moore) Bremek.** | Rubiaceae | hadaita (K) | Shrub | Fruit, Underground part | 1 | 0.03 | 0.33 | 135 |
| 480 | *Teclea nobilis* Del.** | Rutaceae | hadheessa (O, S), qoladie (A) | Tree | Fruit | 4 | 0.11 | 0.17 | 118, 119, 126, 131 |
| 481 | *Teclea salicifolia* Engl.* | Rutaceae | hadheessa (O) | Shrub | Fruit, Other parts | 1 | 0.03 | 0.33 | 127 |
| 482 | *Teclea simplicifolia* (Engl.) Verdoorn* | Rutaceae | Hadheesa (O) | Shrub | Fruit | 1 | 0.03 | 0.17 | 128 |
| 483 | *Terminalia brevipes* Pampanini* | Combretaceae | cigiddiida (O) | Shrub | Fruit | 1 | 0.03 | 0.17 | 127 |
| 484 | *Thymus schimperi* Ronniger* | Lamiaceae | xosinyii (O), tosign (A) | Herb | Leaf, Flower, Other parts | 4 | 0.11 | 0.5 | 42, 96, 97, 126 |
| 485 | *Thymus serrulatus* Hochst. Ex Benth. ** | Lamiaceae | Xooshine (S) | Herb | Leaf, Fruit, Flower, Seed, Other parts | 1 | 0.03 | 0.83 | 131 |
| 486 | *Toddalia asiatica* (L.) Lam.** | Rutaceae | Arbagube (O), Barbari-Burreed (So) | Shrub | Fruit | 2 | 0.05 | 0.17 | 124, 130 |
| 487 | *Tragia pungens* (Forssk.) Muell. Arg.* | Euphorbiaceae | doobii (O) | Shrub | Fruit | 1 | 0.03 | 0.17 | 118 |
| 488 | *Trichiliae metica* Vahl* | Meliaceae | xirdhoo | Tree | Fruit | 1 | 0.03 | 0.17 | 127 |
| 489 | *Trifolium schimperi* A. Rich. * | Fabaceae | grar (A) | Herb | Fruit | 1 | 0.03 | 0.17 | 96 |
| 490 | *Tristemma mauritianum* J.F.Gmel.** | Melastomataceae |  | Shrub | Fruit | 1 | 0.03 | 0.17 | 12 |
| 491 | *Turraea holstii* Gurke* | Meliaceae | Hirqaqamu (O) | Shrub/  Tree | Fruit | 1 | 0.03 | 0.17 | 118 |
| 492 | *Tylosema fassoglensis* (Kotschy ex Schweinf.) Torre &Hillc. ** | Fabaceae | Dankilo (M) | Climber | Seed | 2 | 0.05 | 0.17 | 114, 135 |
| 493 | *Typha angustifolia* L.* | Typhaceae |  | Herb | Flower | 1 | 0.03 | 0.17 | 102 |
| 494 | *Typha domingensis* Pers.* | Typhaceae | Kaylampa (K) | Herb | Flower, Underground part | 1 | 0.03 | 0.33 | 135 |
| 495 | *Urtica simensis* Hochst. Ex A.Rich.** | Urticaceae | Gurgubee/Laalesa/Doobbii (O), Sama (A), Laaleessa (S), Amae (T) | Herb | Leaf, Other parts | 11 | 0.29 | 0.33 | 42, 93, 96, 97, 104, 115, 116, 118, 126, 131, 132, |
| 496 | *Urtica urens* L.* | Urticaceae | Semsamo (A) | Herb | Leaf | 1 | 0.03 | 0.17 | 97 |
| 497 | *Uvaria leptocladon* Oliv.** | Annonaceae | Muralatse (M) | Shrub | Fruit | 1 | 0.03 | 0.17 | 114 |
| 498 | *Vangueria apiculata* K. Schum.** | Rubiaceae | Timpliqisha (K), Garo (M), Bururii (O), Hawa (Sh) | Shrub | Fruit | 4 | 0.11 | 0.17 | 94, 114, 127, 136 |
| **No** | **Scientific name** | **Family name** | **Local name** | **Habit** | **Parts used** | **FC** | **RFC** | **RUV** | **References** |
| 499 | *Vangueria madagascariensis* J.F. Gmel.** | Rubiaceae | Surangaro (M), Gara (Ar) | Shrub | Fruit | 2 | 0.05 | 0.17 | 114, 136 |
| 500 | *Vangueria madagascariensis* J.F. Gmel. Var. abyssinica (A.Rich.) Puff* | Rubiaceae | Mudukanta/Dimbliksha (K), | Shrub | Fruit | 1 | 0.03 | 0.17 | 135 |
| 501 | *Vangueria madagascariensis* J.F. Gmel. Var. madagascarensis* | Rubiaceae | Mudukanta/Dimbliksha (K), | Shrub | Fruit | 2 | 0.05 | 0.17 | 134, 135 |
| 502 | *Vatovaea pseudolablab* (Harms) J. B. Gillet** | Fabaceae | Kullayya/Kuliakurappo (K) | Climber | Underground part | 2 | 0.05 | 0.17 | 135, 136 |
| 503 | *Vepris dainellii* (Pichi-Serm.) Kokwaro* | Rutaceae | hadhessa (O), Lelcho (S), arabee | Tree | Fruit | 4 | 0.11 | 0.17 | 100, 118, 126, 131 |
| 504 | *Vernonia amygdalina* Del.* | Asteraceae | eebicha (O), Hechcho (S), banjaga (G), grawa (A) | Shrub | Leaf, Other parts | 4 | 0.11 | 0.33 | 94, 96, 126, 131 |
| 505 | *Vernonia auriculifera* Hiern* | Asteraceae | Igidimba (G), saraji (O) | Shrub | Fruit, Leaf | 2 | 0.05 | 0.33 | 118, 123 |
| 506 | *Vernonia theophrastifolia* schweinf. ex Oliv. &Hiern* | Asteraceae | agidema/anjidema (G) | Shrub | Leaf | 1 | 0.03 | 0.17 | 123 |
| 507 | *Vigna subterranea* (L.) Verdc. * | Fabaceae | akala (G) | Shrub | Seed | 1 | 0.03 | 0.17 | 123 |
| 508 | *Vigna vexillata* (L.) Rich. ** | Fabaceae | KafiAtsa (Sh) | Herb | Underground part | 1 | 0.03 | 0.17 | 121 |
| 509 | *Vitellaria paradoxa* C.F.Gaertn.** | Sapotaceae |  | Tree | Fruit, Seed, Other parts | 1 | 0.03 | 0.5 | 117 |
| 510 | *Vitex doniana* Sweet** | Lamiaceae | worogoroki (M), gorka (Ar), qoqora (G), kokor (Sh), plem (A) | Tree | Fruit | 5 | 0.13 | 0.17 | 94, 114, 116, 117, 123 |
| 511 | *Ximenia americana* L.** | Olacaceae | Hudha/akuku (O), Enkoy/kol (A), mukla (Ar), mullancho (B), helelea (Af), mlo (T), feya/meyo/heya(G) | Shrub/  Tree | Fruit | 26 | 0.68 | 0.17 | 42, 91, 92, 93, 94, 95, 96, 98, 99, 100, 104, 102, 114, 115, 116, 117, 119, 123, 125, 126, 130, 132, 133, 134, 137, 138 |
| 512 | *Ximenia caffra* Sond. ** | Olacaceae | Hinkiketa/Enkoy (K), mukalee (M), ingigita (B), hudhaa | Shrub | Fruit | 9 | 0.24 | 0.17 | 98, 102, 114, 116, 117, 127, 134, 135, 136 |
| 513 | *Xylopia aethiopica* (Dunal) A. Rich. * | Annonaceae | uda (O) | Tree | Fruit | 1 | 0.03 | 0.17 | 124 |
| 514 | *Zanthoxylum chalybeum* Engl.** | Rutaceae | Kettata (K) | Tree | Leaf, Fruit | 2 | 0.05 | 0.33 | 134, 135 |
| 515 | *Zehneria scabra* (Linn. f.) Sond. * | Cucurbitaceae | suruphaa (O) | Climber | Fruit | 1 | 0.03 | 0.17 | 127 |
| 516 | *Zingiber officinale* Roscoe* | Zingiberaceae | Ajanzibil (G) | Herb | Underground part | 1 | 0.03 | 0.17 | 123 |
| **No** | **Scientific name** | **Family name** | **Local name** | **Habit** | **Parts used** | **FC** | **RFC** | **RUV** | **References** |
| 517 | *Ziziphus abyssinica* Hochst. ex A. Rich. ** | Rhamnaceae | Kobtta (K), Abetere (A), huqunqura (O), hanguga (G) | Tree/  Shrub | Fruit | 8 | 0.21 | 0.17 | 94, 102, 116, 117, 123, 127, 133, 135 |
| 518 | *Ziziphus jujuba* Mill. * | Rhamnaceae | kurkura (A) | Tree | Fruit | 1 | 0.03 | 0.17 | 116 |
| 519 | *Ziziphus mucronata* Willd. ** | Rhamnaceae | kobta (K), KUSIR-A (-TO) (Af); KURKURA HADO, qurqura/buqunqura (O), yezenjerogeba/ado-qurqura (A), ado kurkura (A), wkerura (Ko), qunqura (T) | Shrub/Tree | Fruit | 14 | 0.37 | 0.17 | 91, 92, 93, 102, 116, 117, 118, 120, 124, 130, 132, 134, 135, 136, |
| 520 | *Ziziphus spina-christi* (L.) Desf. ** | Rhamnaceae | QurquraMi'o (O), KUSIR-A (-TO)/Qusura (Af); KURKURA (O), Arka/gaba/qurqura (A), gaudii (M), jujube (T), kurqura (Ko), sirah/serwie (G) | Tree | Fruit | 20 | 0.53 | 0.17 | 92, 93, 94, 95, 97, 99, 102, 104, 114, 116, 117, 119, 124, 125, 129, 132, 133, 134, 137, 138 |

**Supplementary file 2** Wild edible plants of Ethiopia documented before 2011 (Reproduced by obtaining permission from the corresponding author)

Abbreviations and Symbols **Local names**: Afa-Afar, Amh-Amharic, Anu-Anuak, Awi-Awi, Ben-Bena, Bench-Bench, Ber-Berta, D-Derashe, G-Gamo, Gum-Gumuz, Gur-Gurage, Had-Hadiya, Ham-HamarKKusume, KA-Kara, Kaf-Kafficho, Kon-Xonso, KW-Kwego, NM-Not mentioned, Maj-Majanjir, Mur-Mursi, Nue-Nuer, Oro-Affan Oromo, She-Sheko, Sid-Sidamo, Som-Somali, Tig-Tigray, Tse-Tsemay, Wel-Welaita, Zay-Zay. **Habit**: T=tree, S=shrub, H=herb, C=Climber

| **No** | **Scientific name** | **Family** | **Local name** | **Habit** | **Parts used** | **Where in**  **Ethiopia** | **References** |
| --- | --- | --- | --- | --- | --- | --- | --- |
| 1 | *Acalypha fruitcosa* Forssk. | Euphorbiaceae | Keryaya Hola (Mur) | T | Leaf | Mursi and Kaffa | 140 |
| 2 | *Acalypha ornate* A. Rich. | Euphorbiaceae | Atiyhomerpap (Anu) | S | Leaf | Anuak | 146 |
| 3 | *Albizia grandibracteate Taub.* | Fabaceae | Bamu (Anu) | T | Bark | Anuak, Majanjir | 146 |
| 4 | *Allophylus macrobotrys* Gilg | Sapindaceae | Athow (Anu) | T | Fruit | Anuak, Kara and Kwego | 139, 146 |
| 5 | *Amorphophallus abyssinicus* (A. Rich.) N. E. Br. | Araceae | Bagane (Kon) | H | Tuber | Xonso | 140 |
| 6 | *Ampelocissus bombycine* (Bak.) Planch | Vitaceae | Astigena (Gum) | H | Fruit | Benishangul Gumuz | 140 |
| 7 | *Aneilema beniniense* (P. Beauv.) Kunth | Commelinceae | Aretekodo (Anu) | H | Leaf | Gambella | 140 |
| 8 | *Antidesma venosum* Tul. | Euphorbiaceae | Huda (Oro) | H | Fruit | Metu | 140 |
| 9 | *Barleria acanthoides* Vahl | Acanthaceae | Boko (Ham) | S | Flower/nectar | Hamar and Xonso | 5 |
| 10 | *Bidens borianiana* (Sch. Bip. ex Schweinf.) Cufod. | Asteraceae | Ade (Gur) | H | Leaf | Cheha | 141 |
| 11 | *Bidens prestinaria (Sch. Bip.) Cufod.* | Asteraceae | Assegetsiya (Ber) | H | Leaf | Berta | 146 |
| 12 | *Boscia senegalensis Lam*. ex Poir. | Capparidaceae | Tubaqe (Tse) | S | Fruit | South Ethiopia | 140 |
| 13 | *Buddle japolystachya* Fresen. | Loganiaceae | Madera (Afa) | S | Fruit | Afar | 140 |
| 14 | *Butyrospermum paradoxum* (Gaertn. f.) Hepper | Sapotaceae | Wado (Anu) | T | Fruit | Anuak | 146 |
| 15 | *Canthium bogosense* (Martelli) Penzig | Rubiaceae | Ajarse (Som) | S | Fruit | Gursum | 143 |
| 16 | *Capparis decidua* (Forssk.) Edgew | Capparidaceae | Gumero (Amh) | S | Fruit | Wollo | 140 |
| **No** | **Scientific name** | **Family** | **Local name** | **Habit** | **Parts used** | **Where in**  **Ethiopia** | **References** |
| 17 | *Capparis erythrocarpos* Isert | Capparidaceae | Omono (Anu) | S | Fruit | Anuak | 146 |
| 18 | *Capparis fascicularis* DC. | Capparidaceae | Qawisa (Oro) | S | Fruit | Dheeraa | 145 |
| 19 | *Caralluma sprengeri* N. E. Br. | Asclepiadaceae | Baqibaqa (Kon) | S | Leaf | Xonso | 142 |
| 20 | *Catuna regamnilotica* (Stapf)Tirveng | Rubiaceae | Ondorko (Tse) | T | Fruit | Bena | 143 |
| 21 | *Cayratia ibuensis* (Hook.f.) Suesseng | Vitaceae | Daole (Mur) | H | Tuber | Gambella | 140 |
| 22 | *Celtis zenkeri* Engl. | Ulmaceae | Bado (Anu) | S | Fruit | Anuak | 146 |
| 23 | *Cephalopentandrae cirrhosa* (Cogn.) C. Jeffrey | Cucurbitaceae | NM | S | Fruit | Harar | 144 |
| 24 | *Cissus cornifolia* (Bak.) Planch | Vitaceae | Asinsidhi (Ber) | C | Fruit | Berta | 15 |
| 25 | *Cleome allamanii* Chiov. | Capparidaceae | Erreso (Kon) | C | Leaf | Hamar and Xonso | 5 |
| 26 | *Cleome gallaensis* Gilg and Bened | Capparidaceae | Armagussa (Amh) | S | Leaf | Goma | 141 |
| 27 | *Cleome hanburyana* Penz. | Capparidaceae | Kedhi (Ben) | H | Leaf | Humbo | 140 |
| 28 | *Coccinia adoensis* (HochstexA.Rich) Cogn. | Cucurbitaceae | Thong-diit (Nue) | H | Fruit | Nuer | 15, 146 |
| 29 | *Commelina erecta* L. | Commeliaceae | Surnate (Mur) | H | Leaf | Mursi | 140 |
| 30 | *Commelina petersii* Haask. | Commeliaceae | Korde (Ham) | H | Leaf | Hamar and Xonso | 5 |
| 31 | *Commelina zambesica* C.B.Clarke | Commeliaceae | Gnok (Nue) | H | Leaf | Gambella, Komo | 140,146 |
| 32 | *Commiphora boiviniana* Engl. | Burseraceae | Elawa (Kon) | S | Fruit | Sidamo | 140 |
| 33 | *Convolvulus glomeratus* Hochst ex Choisy | Convolvulaceae | Bolok (KW) | H | Leaf | Kara and Kwego | 139 |
| 34 | *Corchorus aestuans* L. | Tiliaceae | Awachuwaey (Anu) | H | Leaf | Anuak | 146 |
| 35 | *Corchorus fascicularis* Lam. | Tiliaceae | Awachuwaey (Anu) | H | Leaf | Gambella | 140 |
| 36 | *Cordia ovalis R.Br.ex DC.* | Boraginaceae | Luketa (D) | S | Fruit | Derashe and Kucha | 41 |
| 37 | *Crassocephalum montuosum* (S. Moore) Milne-Redh | Asteraceae | Miningi(Maj) | H | Leaf | Gambella | 140 |
| 38 | *Crassocephalum rubens* (Juss. ex Jacq.) S. Moore | Asteraceae | Shekaadona(Ber) | H | Leaf | Berta | 146 |
| 39 | *Crotalaria phillipsiae* Bak. | Fabaceae | Denqesha (Ham) | H | Leaf | Hamar and Xonso | 5 |
| 40 | *Cucumella kelleri* (Cogn.) C. Jeffrey | Cucurbitaceae | Uneexo(Som) | C | Fruit | Degahabur | 143 |
| **No** | **Scientific name** | **Family** | **Local name** | **Habit** | **Parts used** | **Where in**  **Ethiopia** | **References** |
| 41 | *Cucumis jeffreyanus* Thulin | Cucurbitaceae | Qalfon (Som) | S | Fruit | Somali | 143 |
| 42 | *Cucumis pustulatus* Naud. ex-Hook. f. | Cucurbitaceae | Qalfoon (Som) | C | Fruit | Degahabur | 143 |
| 43 | *Cymbopogon caesiu* (Hook. & Arn.) Stapf | Poaceae | GnieeraWoni (Ber) | H | Inflorescence | Berta | 146 |
| 44 | D*ioscorea dumetorum* (Kunth) Pax | Dioscoreaceae | NM | H | Root | Gambella | 15, 140 |
| 45 | *Dioscorea quartinana A*. Rich. | Dioscoreaceae | Kuba (Oro) | C | Tubers | Kafa | 140 |
| 46 | *Dombeya longibracteolata* Seyani | Sterculiaceae | Kamil (Ham) | S | Fruit | Hamar and Xonso | 5 |
| 47 | *Dombeya torrida* (G.F. Gmel.) P. Bamps | Sterculiaceae | Akota (K) | T | Fruit | Kusume, Derashe and Kucha | 140 |
| 48 | *Dracaena afromontana* Mildbr. | Dracaenaceae | Shuda (Kaf) | S | Young shoots | Sheko and Bench-Menit | 140 |
| 49 | *Elaeodendron buchananii* (Loes.) Loes | Celastraceae | Chogaey (Maj) | T | Fruit | Majanjir | 146 |
| 50 | *Eragrostis papposa* (Roem. & Schult.) Steud. | Poaceae | Qercha (Ham) | H | Seed | Hamar and Xonso | 5 |
| 51 | *Eragrostis tremula* Hochst. ex Steud | Poaceae | Buska (Ham) | H | Seed | Hamar and Xonso | 5 |
| 52 | E*riobotrya japonica* (Thunb.) Lindl. | Rosaceae | Woshimela (Amh) | T | Fruit | Goma | 141 |
| 53 | *Erucastrum arabicum* Fisch. & Mey. | Brassicaceae | Shimpa (Oro) | H | Leaf and stem | Many parts of Ethiopia | 140 |
| 54 | *Erythrina Brucei* Schweinf. | Fabaceae | Korch (Amh) | T | Root | Dheeraa | 145 |
| 55 | *Ethulia gracilis* Del. | Asteraceae | Apuda (Anu) | H | Whole part burned | Anuak, Nuer | 146 |
| 56 | *Ficus mucuso* Ficalho | Moraceae | Shola (Bench) | T | Fruit | Gambella and Bench Menit | 140 |
| 57 | *Garcinia ovalifolia* Oliver | Clusiaceae | Karawwayyuu (Maj) | T | Fruit | Gambella& Metu | 140 |
| 58 | *Gardenia fiorii* Chiov. | Rubiaceae | Himir (Som) | S | Fruit | Wardheer | 143 |
| 59 | *Giardinia diversifolia (Link) Friis* | Urticaceae | Doba (Tig) | H | Leaf | Darassa | 140 |
| **No** | **Scientific name** | **Family** | **Local name** | **Habit** | **Parts used** | **Where in**  **Ethiopia** | **References** |
| 60 | *Grewia kakothamnos* K. Schum. | Tiliaceae | Demak (KA) | S | Fruit | Kara and Kwego | 139 |
| 61 | *Heliotropium steudneri* Vatke | Boraginaceae | Gabo (KA) | T | Fruit | Kara and Kwego | 139 |
| 62 | *Hibiscus calyphyllus* Cavan | Malvaceae | Gnilorbey (Anu) | H | Leaf | Anuak | 146 |
| 63 | *Hydnora johannis* Becc. | Hydnoraceae | Likeh (Som) | H | Roots | Deghabour, Hamar and Xonso | 5, 140 |
| 64 | *Hygrophila schulli* (Hamilt.) M.R. & S.M. Almeida | Acanthaceae | Utiwaello (Anu) | H | Whole dried | Gambella | 140 |
| 65 | *Hygrophila spiciformis* Lindau | Acanthaceae | Utiwaello (Anu) | S | Leaf and wood ash | Gambella | 146 |
| 66 | *Hyphaene compressa H*. Wendl | Arecaceae | Annui (Mur) | T | Fruit | Mursi | 140 |
| 67 | *Hypoestes aristata* (Vahl) Roem. & Schult. | Acanthaceae | Hamshika (Oro) | H | Leaf | Metu | 140 |
| 68 | *Ipomoea aquatica* Forssk. | Convolvulaceae | Tach (Nue) | H | Leaf | Gambella, Komo | 15, 140 |
| 69 | *Ipomoea plebeian* R. Br. | Convolvulaceae | Boloko (KA) | S | Leaf | Kara and Kewego | 139 |
| 70 | *Lantana rhodesiensis* Mold. | Verbenaceae | Untaorayitate (D) | S | Seed and leaf | Derashe and Kucha | 41 |
| 71 | L*annea malifolia* (Chiov.) Sacl. | Anacardiaceae | Wuh-Andri (Som) | T | Fruit and seed | Somali | 140 |
| 72 | *Lannea schweinfurthii* (Engl.) Engl. | Anacardiaceae | Kiringenni (Mur) | T | Fruit | Mursi &Gambella | 140 |
| 73 | *Lannea welwitschia* (Hiern) Engl. | Anacardiaceae | Arim (Anu) | T | Fruit | Anuak, Komo | 146 |
| 74 | *Lantana ukambensis* (Vatke) Verdc. | Verbenaceae | Untaorayitate(Der) | S | Leaf | Derashe | 140 |
| 75 | *Leonotis nepetifolia* (L.) R. Br. | Lamiaceae | Angesho (Ber) | H | Nectar | Berta | 146 |
| 76 | *Lecaniodiscus fraxinifolius Bak.* | Sapindaceae | Choro (KA) | T | Fruit | Kara and Kwega | 139 |
| **No** | **Scientific name** | **Family** | **Local name** | **Habit** | **Parts used** | **Where in**  **Ethiopia** | **References** |
| 77 | *Limnophyton obtusifolium* (L.) Miq. | Alismataceae | Tuytuy (Anu) | H | Whole Ash | Anuak | 146 |
| 78 | *Lycium shawii* Roem. & Schult. | Solanaceae | Doreda(KA) | T | Leaf | Kara and Kwego | 139 |
| 79 | *Maerua oblongifolia* (Forssk.) A. Rich | Capparidaceae | Lecho (KA) | S | Leaf | Kara and Kwego, Mursi | 139,140 |
| 80 | *Maerua triphylla* A. Rich. | Capparidaceae | Anaedo (Anu) | S | Leaf | Anuak | 146 |
| 81 | *Momordica rostrata* A. Zimm. | Cucurbitaceae | Kulo (Ham) | C | Fruit and leaf | Hamar and Xonso | 5 |
| 82 | *Nicandra physaloides* (L.) Gaertn. | Solanaceae | Peet (Nue) | H | Fruit | Nuer | 146 |
| 83 | *Nymphaea lotus* L. | Nymphaeaceae | Kutako (KA) | H | Root | Kara and Kwego | 139 |
| 84 | *Olea capensis subsp. macrocarpa* (C.A. Wright.) Verdc. | Oleaceae | Bulumtsee (Ber) | T | Fruit | Berta | 146 |
| 85 | *Opuntia stricta* (Haworth) Haworth | Cactaceae |  | S | Fruit and leaf | Komo | 146 |
| 86 | *Ormocarpum trichocarpum* (Taub.) Engl. | Fabaceae | Shibde (Tse) | S | Flower | Tsemay | 140 |
| 87 | *Oryza barthii A. Chev.* | Poaceae | Alumo (Anu) | H | Seed | Anuak | 146 |
| 88 | *Oryza longistaminata A. Chev. & Roehr.* | Poaceae | Alumo (Anu) | H | Seed | Anuak, Nuer | 146 |
| 89 | *Pachycymbium sprengeri* (N.E.Br.) M.G. Gilbert | Asclepiadaceae | Baqibaqa (Kon) | H | Young shoot | Xonso | 140 |
| 90 | *Pavetta gardenifolia* A. Rich. | Rubiaceae | Shambulo (Ham) | S | Fruit | Hamar and Xonso | 5 |
| 91 | *Pentarrhinum somaliense* (N.E. Br.) Liede | Asclepiadacee | Guriso (Tig) | S | Fruit and seed | Alamata | 141 |
| 92 | *Pergularia daemia* (Forssk.) Chiov. | Asclepiadacee | Korroda (Kon) | C | Leaf | Xonso | 140 |
| **No** | **Scientific name** | **Family** | **Local name** | **Habit** | **Parts used** | **Where in**  **Ethiopia** | **References** |
| 93 | *Phyllantus boehmii Pax* | Euphorbiceae | Butbot (Nue) | H | Leaves and young shoots | Nuer | 146 |
| 94 | *Phyllanthus limmuensis* Cufod. | Euphorbiceae | Karacho (Mur) | S | Fruit | Mursi | 140 |
| 95 | *Physalis micrantha* Link | Solanaceae | Yefereng Awit (Amh) | S | Fruit | Wonji | 143 |
| 96 | *Phytolaca dodecandra* L. H´erit. | Phytolacaceae | Indod (Amh) | S | Leaf | Goma | 141 |
| 97 | *Plectranthus edulis* (Vatke) Agnew | Lamiaceae | Ajo (Kaf) | H | Rhizome and leaves | Kafa | 140 |
| 98 | *Pouteria altissima* (A. Chev.) Baehni | Sapotaceae | Gomu (Maj) | T | Fruit | Gambella, Majanjir | 140, 146 |
| 99 | *Pycnostachys abyssinica* Fresen. | Lamiaceae | Fanfua (Gur) | S | Leaf | Cheha | 141 |
| 100 | *Pyrenacantha kaurabassana* Baill. | Icacinaceae | Appel (Anu) | C | Tubers | Anuak, Komo | 15 |
| 101 | *Rhoicissus revoilii* Planch. | Vitaceae | Daga-Cebsa (Oro) | C | Fruit | Gambella | 140 |
| 102 | *Rubus aethiopicus* R. A. Grah. | Rosaceae | Hinjaro (Had) | S | Fruit | Many parts of Ethiopia | 140 |
| 103 | *Rubus erlangeri* Engl. | Rosaceae | Henjoriya (Wel) | S | Fruit | Many parts of Ethiopia | 140 |
| 104 | *Saccharum spontaneum* L. | Poaceae | Maqesha (D) | H | Stem | Derashe and Kucha | 41 |
| 105 | *Sacrocephalus latifolius* (Smith) N. E. Bruce | Rubiaceae | Moyo (Anu) | S | Fruit | Komo | 15 |
| 106 | *Satyrium aethiopicum* Summerh. | Orchidaceae | Aziburt (Gur) | H | Tuber | Cheha | 141 |
| 107 | *Sclerocarya birrea subsp.birrea* (A.Rich.) Hochst. | Anacardiaceae | Pasha (D) | T | Fruit and seed | Derashe, Gamo, Kusue, Mursi, Zeyise, Komo, Majanjir, Nuer | 15, 41 |
| **No** | **Scientific name** | **Family** | **Local name** | **Habit** | **Parts used** | **Where in**  **Ethiopia** | **References** |
| 108 | *Scolopia theifolia* Gilg | Flacourtiaceae | Kokofla (Oro) | T | Fruit | Menagesha | 140 |
| 109 | *Scutia myrtina* (Burm. f.) Kurz | Rhamnaceae | Haraang (Oro) | T | Fruit | Dheeraa | 145 |
| 110 | *Solanum memphiticum* Gmel. | Solanaceae | NM | S | Fruit | Shashamane | 143 |
| 111 | *Solanum tarderemotum* Bitter | Solanaceae | NM | S | Fruit and leaf | Dilla | 143 |
| 112 | *Sparmannia ricinocarpa* (Eckl. and Zeyh.) O. Ktze. | Tiliaceae | Wulkifa (Amh) | S | Bark | Alamata | 141 |
| 113 | *Sphenostylissteno carp*a (Hochst.exA. Rich.) Harms | Fabaceae | AdagoraBarracha (Tig) | H | Seed and root | Tigray | 140 |
| 114 | *Sporobolus indicus* (L.) R. Br. | Poaceae | Harataa (Oro) | H | Seed | SeqaChoqorsa | 142 |
| 115 | *Sterculia rhynchocarpa* K. Schum | Sterculiaceae | Qeytso (Ben) | S | Seed | Bena, Tsemay | 140 |
| 116 | *Strychnos mitis* S. Moore | Loganiaceae | Chattto (She) | T | Fruit | Bale | 140 |
| 117 | *Tagetes minuta* L. | Asteraceae | Zwdearem (Amh) | H | Leaf | Dheeraa | 145 |
| 118 | *Talinum portulacifolium* (Forssk.) Aschers. Ex Schwein | Portulacaceae | Dongdongi (Mur) | H | Leaf | Mursi | 140 |
| 119 | *Tribulus terrestris* L. | Zygophyllaceae | Qumputia (Wel) | H | Leaf | North Omo | 140 |
| 120 | *Trichilia dregeana* Sond. | Meliaceae | Gereche (Anu) | T | Seed | Gambella | 140 |
| 121 | *Trilepisium madagascariensis* DC. | Moraceae | Gabo (She) | T | Fruit | Majanjir and Sheko | 140,146 |
| 122 | *Triumfettar homboidea* Jacq. | Tiliaceae | Weeo (Anu) | H | Leaf | Gambella | 140 |
| 123 | *Tropaeolum majus* L. | Tropaeolaceae | NM | H | Fruit | Cheha | 141 |
| 124 | *Uvaria angolensis* Oliv. | Annonaceae | Boyinya (Wel) | S | Fruit | North Omo | 140 |
| 125 | *Vepris eugenifolia* (Engl.) Verdoorn | Rutaceae | Tsaki (Ham) | S | Fruit | Hamar and Xonso | 5 |
| 126 | *Vepris glomerata* (F. Hoffm.) Engl. | Rutaceae | Kena (Ham) | C | Fruit | Hamar and Xonso | 5 |
| **No** | **Scientific name** | **Family** | **Local name** | **Habit** | **Parts used** | **Where in**  **Ethiopia** | **References** |
| 127 | *Vigna membranacea* A. Rich. | Fabaceae | Bog Ajowm (Anu) | H | Leaf | Anuak, Berta, Komo | 146 |
| 128 | *Vigna unguiculat*a (L.) Walp. | Fabaceae | Shirshira (Kon) | H | Tuber | Xonso | 142 |
| 129 | *Whitfieldiae longata* (P. De Beauv.) De Wild. &T. Durand | Acanthaceae | Adibuch (G) | S | Nectar | Berta | 146 |
| 130 | *Ziziphus hamur* Engl. | Rhamnaceae | Haamud (Som) | S | Fruit | Harar | 41,140 |
| 131 | *Ziziphus mauritiana* Lam. | Rhamnaceae | Gusura (Afa) | T | Fruit | Derashe and Kucha, Afar and Gamo | 41,140 |

**Supplementary file 3 Wild edible plants sold in local markets in the reviewed articles**

| **No** | **Name of the WEP** | **Frequency of citations in the reviewed articles** |
| --- | --- | --- |
| 1 | *Balanites aegyptiaca* (L.) Delile | 11 |
| 2 | *Ximenia americana* L. | 10 |
| 3 | *Balanites rotundifolia* (Tiegh.) Blatt. | 7 |
| 4 | *Tamarindus indica* L. | 7 |
| 5 | *Ziziphus spina-christi* (L.) Desf. | 7 |
| 6 | *Mimusops kummel* Bruce ex A.DC. | 6 |
| 7 | *Opuntia ficus-indica* (L.) Miller | 5 |
| 8 | *Syzygium guineense* (Willd.) DC. | 5 |
| 9 | *Berchemia discolor* (Klotzsch) Hemsl. | 4 |
| 10 | *Carissa spinarum* L*.* | 4 |
| 11 | *Syzygium guineense supsp. guineense* (Willd.) DC. | 4 |
| 12 | *Cordia africana* Lam. | 3 |
| 13 | *Dobera glabra* (Forssk.) Juss. ex Poir. | 3 |
| 14 | *Corchorus olitorius* L. | 2 |
| 15 | *Diospyros abyssinica* (Hiern) F. White | 2 |
| 16 | *Diospyros mespiliformis* Hochst. ex A. DC. | 2 |
| 17 | *Ficus sur* Forssk. | 2 |
| 18 | *Ficus vasta* Forssk. | 2 |
| 19 | *Grewia tenax* (Forssk.) Fiorii | 2 |
| 20 | *Grewia villosa* Willd. | 2 |
| 21 | *Leptadenia hastata* Vatke | 2 |
| 22 | *Manilkara butugi* Chiov. | 2 |
| 23 | *Olea europaea* L. *subsp. cuspidata* | 2 |
| 24 | *Rhamnus prinoides* L’Herit. | 2 |
| 25 | *Saba comorensis* (Boj.) Pichon | 2 |
| 26 | *Solanum nigrum* L. | 2 |
| 27 | *Ximenia caffra* Sond. | 2 |
| 28 | *Ziziphus mucronata* Willd. | 2 |
| 29 | *Acacia etbaica* Schweinf. | 1 |
| 30 | *Acacia senegal* (L.) Willd. | 1 |
| 31 | *Acacia seyal* Delile | 1 |
| 32 | *Adansonia digitata* L. | 1 |
| 33 | *Aframomum corrorima* (Braun) Jansen | 1 |
| 34 | *Amaranthus caudatus* L. | 1 |
| **No** | **Name of the WEP** | **Frequency of citations in the reviewed articles** |
| 35 | *Amaranthus hybridus* L. | 1 |
| 36 | *Arisaema schimperianum* Schott | 1 |
| 37 | *Blyttia fruticulosa* (Decne.) D.V. Field | 1 |
| 38 | *Boswellia neglecta* S.Moore | 1 |
| 39 | *Brassica rapa* L. | 1 |
| 40 | *Cleome monophylla* L. | 1 |
| 41 | *Colocasia esculenta* (L.) Schott | 1 |
| 42 | *Commiphora africana* Engl. | 1 |
| 43 | *Commiphora baluensis* Engl. | 1 |
| 44 | *Cordia monoica* Roxb. | 1 |
| 45 | *Cordia sinensis* Lam. | 1 |
| 46 | *Cucurbita pepo* L. | 1 |
| 47 | *Digera muricata* (L.) Mart. | 1 |
| 48 | *Ehretia cymosa* Thonn. | 1 |
| 49 | *Ensete ventricosum* (Welw.) Cheesman | 1 |
| 50 | *Ficus ovata* Vahl | 1 |
| 51 | *Ficus platyphylla* Del. | 1 |
| 52 | *Ficus sycomorus* L. | 1 |
| 53 | *Grewia bicolor* Juss. | 1 |
| 54 | *Grewia flavescens* Juss. | 1 |
| 55 | *Grewia mollis* Juss. | 1 |
| 56 | *Grewia schweinfurthii* Burret | 1 |
| 57 | *Hibiscus cannabinus* L. | 1 |
| 58 | *Lantana camara* L. | 1 |
| 59 | *Meyna tetraphylla* (Schweinf. ex Hiern) Robyns | 1 |
| 60 | *Moringa stenopetala* (Baker f.) Cufod. | 1 |
| 61 | *Morus alba* L. | 1 |
| 62 | *Ocimum urticifolium* Roth | 1 |
| 63 | *Piper capense* L. f. | 1 |
| 64 | *Portulaca quadrifida* L. | 1 |
| 65 | *Prunus africana* (Hook.f.) KalkMAN | 1 |
| 66 | *Rosa abyssinica* R.Br.ex Lindley. | 1 |
| 67 | *Rubus volkensii* Engl. | 1 |
| 68 | *Rumex nervosus* Vahl | 1 |
| 69 | *Salvadora persica* L. | 1 |
| **No** | **Name of the WEP** | **Frequency of citations in the reviewed articles** |
| 70 | *Schinus molle* L. | 1 |
| 71 | *Sclerocarya birrea* (A. Rich.) Hochst. | 1 |
| 72 | *Sterculia africana* (Lour.) Fiori | 1 |
| 73 | *Syzygium guineense supsp. afromontanum* F. White | 1 |
| 74 | *Syzygium guineense subsp. macrocarpa* (Engl.) F. White | 1 |
| 75 | *Thymus serrulatus* Hochst. Ex Benth. | 1 |
| 76 | *Toddalia asiatica* (L.) Lam. | 1 |
| 77 | *Urtica simensis* Hochst. Ex A.Rich. | 1 |
| 78 | *Vangueria madagascariensis* J.F. Gmel. | 1 |
| 79 | *Vangueria madagascariensis* J.F. Gmel. *Var. madagascarensis* | 1 |
| 80 | *Vepris dainellii* (Pichi-Serm.) Kokwaro | 1 |
| 81 | *Vitex doniana* Sweet | 1 |
| 82 | *Xylopia aethiopica* (Dunal) A.Rich. | 1 |
